# Alkoxyamines Designed as Potential Drugs against *Plasmodium* and *Schistosoma* Parasites

**DOI:** 10.3390/molecules25173838

**Published:** 2020-08-24

**Authors:** Thibaud Reyser, Tung H. To, Chinedu Egwu, Lucie Paloque, Michel Nguyen, Alexandre Hamouy, Jean-Luc Stigliani, Christian Bijani, Jean-Michel Augereau, Jean-Patrick Joly, Julien Portela, Jeffrey Havot, Sylvain R. A. Marque, Jérôme Boissier, Anne Robert, Françoise Benoit-Vical, Gérard Audran

**Affiliations:** 1 Laboratoire de Chimie de Coordination du CNRS, LCC-CNRS, Université de Toulouse, CNRS, 31555 Toulouse, France; thibaud.reyser@lcc-toulouse.fr (T.R.); chinedu.egwu@lcc-toulouse.fr (C.E.); lucie.paloque@lcc-toulouse.fr (L.P.); michel.nguyen@lcc-toulouse.fr (M.N.); alexandre.hamouy@gmail.com (A.H.); jean-luc.stigliani@lcc-toulouse.fr (J.-L.S.); bijani@lcc-toulouse.fr (C.B.); jean-michel.augereau@lcc-toulouse.fr (J.-M.A.); 2 Institut de Pharmacologie et de Biologie Structurale, IPBS, Université de Toulouse, CNRS, UPS, 31077 Toulouse, France; 3 Aix Marseille University, CNRS, ICR, UMR 7273, Case 551, Avenue Escadrille Normandie-Niemen, 13397 Marseille CEDEX 20, France; haitung.chem@gmail.com (T.H.T.); jp.joly@live.fr (J.-P.J.); jeff94lb@gmail.com (J.H.); 4 S.A.S ParaDev, 52 Avenue Paul Alduy, 66860 Perpignan, France; julien.portela@paradev.fr; 5 Laboratoire Interactions Hôtes-Pathogènes-Environnements (IHPE), UMR 5244 CNRS, University of Perpignan, IFREMER, Univ. Montpellier, F-66860 Perpignan, France; 6 INSERM, Institut National de la Santé et de la Recherche Médicale, 31024 Toulouse, France

**Keywords:** alkoxyamine, alkylation, heme, malaria, radical chemistry, schistosomiasis

## Abstract

Malaria and schistosomiasis are major infectious causes of morbidity and mortality in the tropical and sub-tropical areas. Due to the widespread drug resistance of the parasites, the availability of new efficient and affordable drugs for these endemic pathologies is now a critical public health issue. In this study, we report the design, the synthesis and the preliminary biological evaluation of a series of alkoxyamine derivatives as potential drugs against *Plasmodium* and *Schistosoma* parasites. The compounds (*RS/SR*)-**2F**, (*RR/SS*)-**2F**, and **8F**, having IC_50_ values in nanomolar range against drug-resistant *P. falciparum* strains, but also five other alkoxyamines, inducing the death of all adult worms of *S. mansoni* in only 1 h, can be considered as interesting chemical starting points of the series for improvement of the activity, and further structure activity, relationship studies. Moreover, investigation of the mode of action and the rate constants *k*_d_ for C-ON bond homolysis of new alkoxyamines is reported, showing a possible alkyl radical mediated biological activity. A theoretical chemistry study allowed us to design new structures of alkoxyamines in order to improve the selectivity index of these drugs.

## 1. Introduction

Malaria and schistosomiasis (=bilharziasis) are two human blood parasitic diseases that are widespread in tropical countries and often associated with coinfections. Current malaria treatments are based on artemisinin combination therapies (ACT) consisting of a semisynthetic derivative of the peroxide drug, artemisinin, in association with an antimalarial drug belonging to another therapeutic class. However, resistance of parasites to ACT is widespread in Southeast Asia [1,2], and artemisinin-resistant parasites display multi-resistant phenotypes [2,3,4,5]. In spite of this worrying situation, artemisinin and its derivatives are still the most efficient and rapidly acting antimalarial drugs, and the cornerstone of the therapy. Against schistosomiasis, praziquantel has been the mainstay of therapy for 40 years, but low sensitivity of parasites in many places have been reported for at least two decades [6]. We recently reported that the synthetic peroxide-based drugs, trioxaquines, are active against all the parasitic stages of both *Plasmodium falciparum* and *Schistosoma mansoni*, and have a synergistic effect when combined with praziquantel [6,7].

Human hemoglobin is digested by the blood parasites, *Plasmodium* and *Schistosoma*, to provide the amino acids required for the synthesis of parasite proteins. This metabolism results in the release of the hemoglobin cofactor, Fe(II)-heme, which is toxic for the parasites, due to its capacity to induce the catalytic reduction in dioxygen leading to a fatal oxidative stress via Fenton chemistry. Both *Plasmodium* and *Schistosoma* share the same vital detoxification pathway, namely, the Fe(II)-heme polymerization, into an insoluble Fe(III) polymer, called hemozoin, which is unable to reduce dioxygen and, thus, is devoid of toxicity. The antimalarial activity of artemisinin involves the reductive activation of its peroxide function by Fe(II)-heme, generating a C-centered radical that alkylates heme to form covalent heme-drug adducts, which are unable to polymerize and, consequently, retain a redox activity that is toxic for the parasites [8,9,10]. Fe(II)-heme is therefore acting as both the trigger and the target of the drug, through a controlled radical process. Such a mechanism of action has been reported for the other peroxide-based drugs [11], trioxaquines [12,13,14,15]. Alkylation of heme by trioxaquines has been evidenced in vivo, both in malaria parasite [14] and in *Schistosoma* [16].

**Figure 1:**
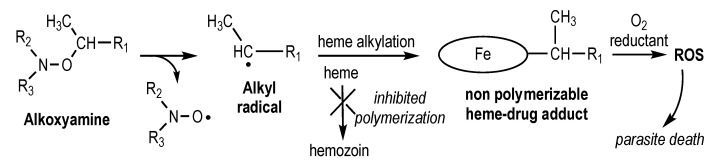
Expected alkylation of heme after homolysis of alkoxyamines, leading to a lethal oxidative stress within blood parasites (the oval stands for the porphyrin macrocycle).

With the aim to develop a series of drugs expected to be active against both malaria and schistosomiasis, we prepared stable alkoxyamine derivatives. Homolysis of the NO-C bond of alkoxyamines, which is the reverse reaction of the well-known trapping of alkyl radical by stable nitroxides, is supposed to occur in vivo, and generate alkyl radicals that are expected to alkylate heme, prevent its polymerization, and cause oxidative lethal damage to the parasites, in a similar fashion to artemisinin and synthetic peroxides (Figure 1). However, due to the absence of the peroxide function, alkoxyamines may maintain their activity against artemisinin-resistant parasites. On this exploratory topic, we report here the synthesis and the in vitro activities of a series of alkoxyamines against *P. falciparum* and *S. mansoni*. As a preliminary investigation of the mechanism of action of these drugs, their reactivity toward heme is reported, as well as a new hypothesis on their antimalarial mechanism of action.

## 2. Results and Discussion

### 2.1. Synthesis of Alkoxyamines

**Figure 2:**
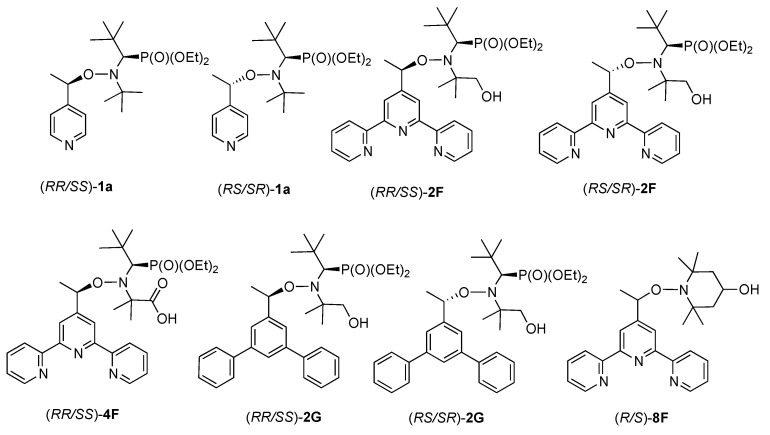
Structures of alkoxyamines **1a**, **2F**, **4F**, **2G** and **8F**.

**Scheme 1:**
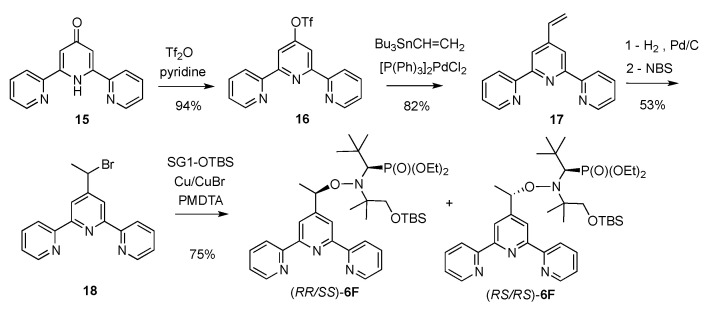
Synthesis of alkoxyamines (RR/SS)-**6F** and (*RS*/*SR*)-**6F**.

**Scheme 2:**
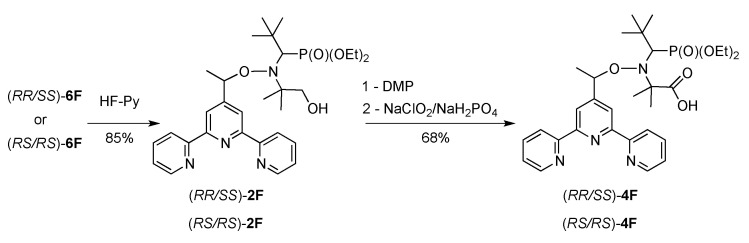
Synthesis of alkoxyamines (*RR*/*SS*)-**2F**, (*RS*/*SR*)-**2F**, (*RR*/*SS*)-**4F** and (*RS*/*SR*)-**4F**.

**Scheme 3:**
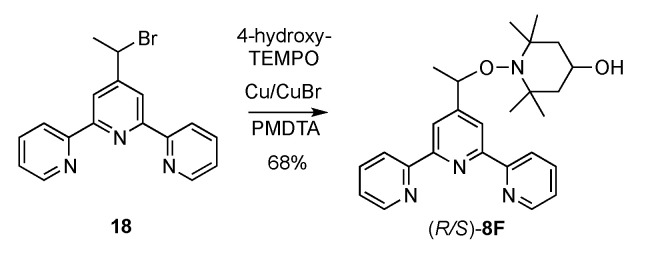
Synthesis of alkoxyamines (R/S)-**8F**.

Among more than a hundred of prepared alkoxyamines (see Figure S1), the derivatives whose synthesis, reactivity and biological activity will be discussed in detail are depicted in Figure 2. Most of the alkoxyamines were prepared as previously reported [17,18,19,20,21,22,23,24,25,26,27]. The syntheses of new compounds are depicted in Scheme 1 (compound **6F**), Scheme 2 (compound **2F** and **4F**), and Scheme 3 (compound **8F**). First, as reported [28], the 4-piperidone **15** was treated with triflic anhydride to yield triflate **16** (94% yield) (Scheme 1). A Stille coupling of vinyltributyltin with triflate **16** produced the arylvinyl derivative **17** (82% yield). Attempts to synthetize the alkoxyamines, **6F** from arylvinyl **17** using metal complexes based on manganese [29], failed, leading us to investigate a new pathway. Thus, hydrogenolysis of arylvinyl **17**, followed by allylic bromination using NBS in CCl_4_, provided the bromide derivative **18**. Furthermore, the coupling of the alkyl radical, generated in situ by the action of copper catalysts to the bromide derivative with the nitroxide SG1-OTBS [23], was successful, producing the alkoxyamines **6F** as a 2:1 mixture of diastereomers (75% yield).

**Figure 3:**
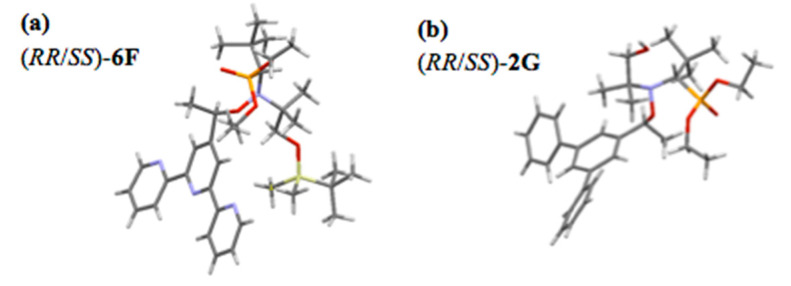
Ortep drawing from X-ray analysis of (**a**) (*RR*/*SS*)-**6F** (Figure 3a) and (**b**) (*RR*/*SS*)-**2G**.

At this stage, the two stereoisomers were easily separated by column chromatography to produce 1.10 g of (*RS*/*SR*)-**6F** and 510 mg of (RR/SS)-**6F,** respectively. Recrystallization of (RR/SS)-**6F** [30] in diethyl ether yielded white single crystals which were X-ray analyzed (Figure 3a).

The following steps were performed on each diastereomer (Scheme 2). Firstly, HF/pyridine [31] deprotection of (*RS*/*SR*)-**6F** yielded the primary alcohol derivative (*RS*/*SR*)-**2F**. Finally, the oxidation of the alcohol into aldehyde under mild conditions using Dess-Martin periodinane (DMP) [32], followed by a Pinnick oxidation procedure [33], provided the carboxylic acid (*RS*/*SR*)-**4F**. Using (*RR*/*SS*)-**6F**, the alcohol (*RR*/*SS*)-**2F**, aldehyde and carboxylic acid (*RR*/*SS*)-**4F** were also prepared using the same methodology as described for (*RS*/*SR*)-**6F**.

The synthesis of the alkoxyamine (*R*/*S*)-**8F** (68% yield) was accomplished by coupling the alkyl radical, generated in situ by the action of copper catalysts to the bromide **18**, with 4-hydroxy-TEMPO.

**Scheme 4:**
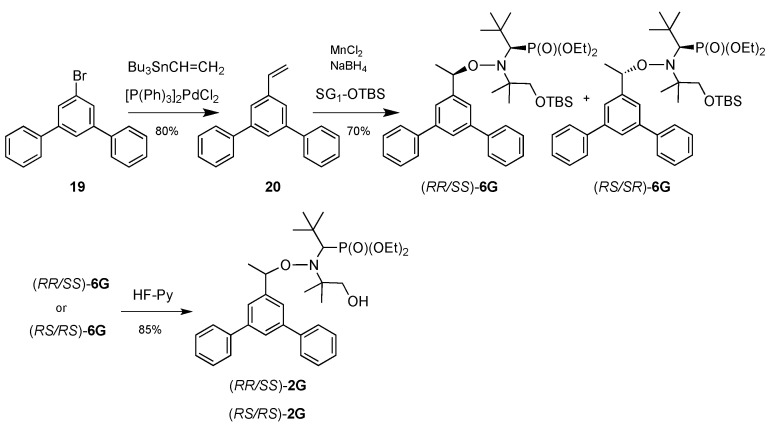
Synthesis of alkoxyamines (*RR*/*SS*)-**2G** and (*RS*/*SR*)-**2G**.

The alkoxyamine **2G** was prepared from commercially available 5′-bromo-**m**-terphenyl **19** (Scheme 4) as follows. Firstly, a Stille coupling using vinyl tributyltin afforded the 5′-vinyl-m-terphenyl **20** in 80% yield. The alkoxyamines (*RR*/*SS*)-**6G** and (*RS*/*SR*)-**6G** were obtained in 70% yield by the use of metal complexes based on manganese [29], and easily separated by column chromatography. The deprotection of (*RS*/*SR*)-**6G** and (*RR*/*SS*)-**6G** using HF/pyridine [32] yielded the alcohols, (*RS*/*SR*)-**2G** and (*RR*/*SS*)-**2G**, respectively, in 85% yield. After purification, recrystallization of (*RR*/*SS*)-**2G** [30] in diethyl ether yielded white single crystals which were X-ray analyzed (Figure 3b).

### 2.2. Antimalarial Activity

We first screened 105 alkoxyamines and moieties on the reference *P. falciparum* strain FcB1-Columbia, twice at 10 µM. Most of the drugs did not induce antiplasmodial activities at 10 µM except 22 molecules which demonstrated a significant parasite growth inhibition at this concentration. These selected molecules were therefore subsequently tested twice in a standard chemosensitivity assay to determine their IC_50_ values. Out of these 22 molecules, 15 exhibited IC_50_ values largely lower than 10 µM with (*RS/SR*)-**2F**, (*RR*/*SS*)-**2F**, and *(R/S)-***8F** as the most active ones with IC_50_ values in the 140–300 nM range (see Supporting Information, Table S1). Due to the large artemisinin resistance challenge in the field, we further evaluated the activity of the 18 drugs exhibiting the lowest IC_50_ values, on both artemisinin-resistant parasites, F32-ART5 and artemisinin-sensitive parasites, F32-TEM. The activities were close to that previously found on the chloroquine-resistant strain FcB1-Columbia. Therefore, we confirmed high antiplasmodial activities of seven molecules, especially (*RS/SR*)-**2F**, (*RR*/*SS*)-**2F**, and *(R/S)-***8F** which were considered as the chemical starting points of the series (Table 1).

Nevertheless, artemisinin resistance is a quiescence-based phenomenon and thus not identifiable by standard assays based on parasite proliferation [35]. In fact, as reported in Table 1, no IC_50_ difference was observed for artemisinin between artemisinin-sensitive and artemisinin-resistant strains. That is why, in order to evaluate whether or not alkoxyamine may exhibit cross resistance with artemisinin, we further tested the compound (*RS/SR*)-**2F** in a recrudescence assay designed to evaluate a putative cross-resistance between artemisinin and (*RS/SR*)-**2F** on artemisinin-resistant F32-ART5 parasites (Table 2).

We found a significant delay in parasite recrudescence between both strains after the (*RS/SR*)-**2F** treatment since the F32-ART5 (artemisinin-resistant) parasites reached the t_0_ initial parasitemia approximately 14 days before the F32-TEM (artemisinin-sensitive) parasites in a similar manner to artemisinin, thus demonstrating a cross-resistance between artemisinin and (*RS/SR*)-**2F**. This result evidenced that alkoxyamine (*RS/SR*)-**2F** is less active against artemisinin-resistant parasites than the artemisinin-sensitive ones (Table 2). To explain this experimental in vitro cross-resistance, we hypothesize that the (*RS/SR*)-**2F** mediated stress induction is likely able to activate the metabolic pathways involved in the quiescence entrance of the artemisinin-resistant parasites.

### 2.3. Antischistosomal Activity

Table 1 summarizes the molecules with the best antischistosomal activities referenced in the Supplementary Table S1. In vitro activity against adult *S. mansoni* was assessed with a set of 74 alkoxyamine derivatives, at 100 µg/mL, corresponding to 150–320 µM. Out of these drugs, 37 induced the death of all the parasites in 1 to 8 h after the drug addition to the parasite culture media (Table S1). Five molecules, (*RR*/*SS*)-**1k**, (*RR*/*SS*)-**1p**, (*RS/SR*)-**1A**, (*RR*/*SS*)-**1A** and **2J**, exhibited the highest antischistosomal activities by killing all the parasites after 1 h of incubation. Furthermore, nine molecules exhibited antischistosomal effects between 1 and 2 h, while nine killed all worms between 2 to 3 h. Finally, eight and five molecules showed antischistosomal activities between 3–4 h and 4–6 h, respectively.

Notably, *(R/S)*-**8F**, (*RS/SR*)-**2F** and (*RR*/*SS*)-**2F** exhibited the highest activity against *P. falciparum*. However, these drugs were not the most active against *S. mansoni*. Indeed, they induced the death of all adult worms in only 5, 2.1 and 2.5 h, respectively. Conversely, (*RR*/*SS*)-**1k**, (*RR*/*SS*)-**1p**, (*RS*/*SR*)-**1A**, and (*RR*/*SS*)-**1A**, displaying the highest activities towards *Schistosoma*, were devoid of significant activity against the FcB1-Columbia *Plasmodium* strain (IC_50_ > 10 μM, Table S1). In the reported alkoxyamine series, compound **2F** was found to be the best compromise for antimalarial and antischistosomal activities, with antiplasmodial IC_50_ values below 1 μM (0.2–0.3 μM on the FcB1 strain, Table S1, and 0.4–0.6 μM on the F32 strains, Table 1), and a mean survival time of adult *S. mansoni* in the 2.1–2.5 h range.

### 2.4. Cytotoxicity

For 20 molecules showing the highest antiparasitic activity, their cytotoxicity was evaluated against the non-cancerous mammalian Vero cell line, in order to determine their selectivity indexes (Table 1). Unfortunately, the whole series of molecules with promising antiplasmodial activity presented weak selectivity indexes with poor cytotoxic/antiparasitic ratios, suggesting that the observed activity against *P. falciparum* is not due to a specific antiparasitic process.

### 2.5. Thermodynamic and Kinetic Data for the Homolysis of the NO–C Bond

Homolysis rate constants *k*_d_’ were measured by EPR and estimated using Equation (1) as reported many times in the literature. Further, *E*_a_ and *k*_d_ were estimated using Equation (2). Results are reported in Table 3.

(1) $$\ln\left(\frac{{\left[nitroxide\right]}_{\infty }-{\left[nitroxide\right]}_{t}}{{\left[nitroxide\right]}_{\infty }}\right)=-k_{d}\cdot t$$

(2) $$E_{a}=-\mathrm{RT}\;\ln\left(\frac{E_{a}}{k_{d}}or\;k_{d}^{\prime }\;\right)$$

The difference in *E*_a_ for diastereoisomers is less than 4 kJ/mol as already reported for molecules in the same family. pH was determined by standard procedure [36]. This difference is due to small differences in steric strain between diastereoisomers, as previously observed, and does not deserve more comment. As expected for *meta*-substituted regioisomer, *E*_a_ of **6G** is very close to the models **1z** and **6z**. Due to the intramolecular hydrogen bonding (IHB) in **2G**, *E*_a_ of **2G** is ca. 4 kJ/mol lower than for **6G** as expected from the models **2Z** and **6Z**. As expected from the IHB-suppressing effect of the MeOH/water solvent mixture, *E*_a_ of **2G** is slightly larger than the one of **2G** (given in *t*-BuPh). As observed for **1a** and **1z**, *E*_a_ of **16** is slightly lower than the one of **6G** due to the slightly more polar alkyl fragment in **6F** than in **6G**. As observed for **6G** and **2G**, *E*_a_ of **2F** is lower by 2–3 kJ/mol according to **6F**. In contrast to **1a**, protonation of **2F** produces a small decrease of 2 kJ/mol in *E*_a_. This unexpected weak effect is ascribed to the distribution of the positive charge on the two heteroaromatic rings in position *meta*, leading to a weaker charge on the *ipso* carbon atom in the alkyl fragment of **2FH^+^**. On the other hand, in water under strong acidic conditions, the expected decrease in *E*_a_ by 7–8 kJ/mol is observed from **2F** to **2FH^+^**. Upon in situ coordination of **2F** with FeCl_2_, a decrease by 1 kJ/mol was observed as expected from the coordination of **1a** with Zn(hfac)^2^, meaning that the partial positive charge is mainly located on the central aryl moiety of **2FFe^2+^**.

Surprisingly, coordination of **2F** by FeCl_3_ produces only a decrease by 2–3 kJ/mol in **2FFe^3+^** in contrast to **2FFe^2+^**. At this time, no rationale is available.

The differences observed between **9F** and **9FH^+^** are similar to those reported for models **9a** and **9z**, and the comments for **1** derivatives hold (for the structure of **9F**, see Supporting Information, Figure S1).

Noteworthy, for the alkoxyamine **2F** containing a terpyridine moiety, protonation of the terpyridine residue resulted in a drastic activation of the homolysis: in an aqueous medium, t_1/2_ were 2.7 and 3.5 days at pH 7.4 for (*RS*/*SR*)-**2F** and (*RR*/*SS*)-**2F**, respectively, compared to 5.6 h and 1 h at pH 1 for (*RS*/*SR*)-**2FH^+^** and (*RR*/*SS*)-**2FH^+^**, respectively. Due to the low pKa of terpyridine, this activation is not expected to be biologically relevant. However, complexation of the terpyridine by a Lewis acid such as Fe^2+^, also provided a significant decrease in the alkoxyamine t_1/2_ value (2 h and 1.8 h at pH 7.4 for (*RS*/*SR*)-**2FFe^2+^** and (*RR*/*SS*)-**2FFe^2+^**, respectively). The homolysis can therefore be activated in the complex in vivo medium, by actually unidentified mechanisms.

### 2.6. Reactivity of Alkoxyamines Toward Heme

Since alkoxyamines were designed to generate radicals with putative alkylating ability toward heme, the homolysis of the drugs, and the fate of the generated alkyl radicals were investigated in the presence of heme, with the aim to correlate heme alkylation with antiplasmodial activity. We first compared the reactivity with heme of two couples of diastereomer racemates: alkoxyamines (*RR*/*SS*)-**1a** and (*RS/SR*)-**1a** on the one hand, which exhibit a very low activity in vitro (IC_50_ > 3 μM on FcB1-columbia *P. falciparum*), and (*RS/SR*)-**2F** and (*RR*/*SS*)-**2F** on the other hand, which exhibit a significantly higher activity (IC_50_ = 0.3 and 0.2 μM, respectively) (see Figure 2 for the structures). The reactivity of **8F** was then compared to that of **2F** made of the same alkyl fragment, but with a different nitroxide moiety. T_1/2_ values of alkoxyamines (*RR*/*SS*)-**1a**/(*RS/SR*)-**1a [17,18]** and (*RR*/*SS*)-**2F**/(*RS/SR*)-**2F** (Table 3) in vitro at 37 °C are in the 2–4 day range in aqueous medium and 17 days and 3–5 days, respectively, in *tert*-butylbenzene, while the t_1/2_ of **8F** in *tert*-butylbenzene is as long as 2.5 years (Table 3). Homolysis of the NO–C bond in vivo may be activated by several factors, namely, protonation or coordination of pyridyl nitrogens; however, t_1/2_ values in these complex conditions are unknown. In the simple in vitro system, the reactions were therefore carried out between 70–100 °C for 3–10 h, to activate the homolysis. Due to the poor solubility of heme in aqueous medium, dimethylsulfoxide was used as solvent. The reactions were monitored by UV-visible spectroscopy and LC-MS. After the given reaction time, NMR analysis of the crude reaction mixtures of alkoxyamines with heme was carried out, after the addition of 2–4 mole equivalents of KCN in order to generate a low spin Fe(III)-heme derivative.

Reaction of **1a**, **2F** or **8F** in the presence of heme. In preliminary assays, the reaction of (*RS/SR*)-**1a** or (*RR*/*SS*)-**1a** with heme was carried out for 6 h at 80 °C, under argon, and exhibited the same results with both starting diastereomers. Monitoring by LC-MS indicated that heme was not modified (t_R_ = 10.3 min, *λ*_max_ = 405 nm, *m/z* = 616 amu), suggesting the absence of reaction on the heme macrocycle. Conversely, (*RR*/*SS*)-**1a**/(*RS*/*SR*)-**1a** (t_R_ = 11.7 and 11.8 min for (*RR*/*SS*)-**1a** and (*RS/SR*)-**1a**, respectively) completely disappeared upon reaction for 6 h at 80 °C, while a new chromatographic peak was detected at t_R_ = 13.7 min, with *m/z* values of 295 and 317 amu, which was assigned to the nitroxide radical (M + H^+^ and M + Na^+^, respectively) derived from (*RR*/*SS*)-**1a**/(*RS*/*SR*)-**1a**. Detection of the nitroxyl radical indicated that homolysis of the alkoxy bond occurred, and that the corresponding alkyl radical (Figure 1) was generated in the medium. However, this radical was unable to react with heme to generate covalent heme-drug adducts.

**Scheme 5:**
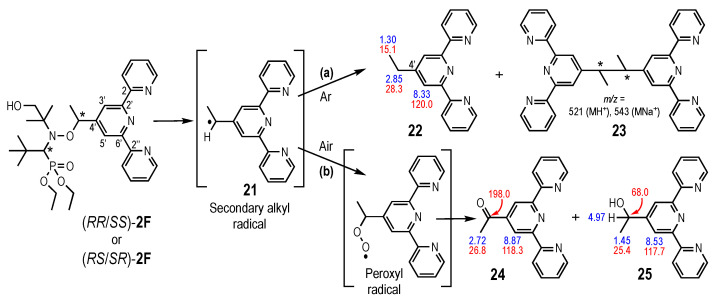
Reaction of (*RR/SS*)-2F or (*RS/SR*)-2F with heme under inert atmosphere (route a) or in the presence of air (route b). The blue and red labels stand for the ^1^H and ^13^C-NMR chemical shifts, respectively.

When (*RS/SR*)-**2F** or (*RR*/*SS*)-**2F** reacted with heme under argon in DMSO-*d*_6_, for 6 h at 95 °C, NMR analysis of the crude reaction mixture allowed characterization of 4′-ethyl terpyridine (compound **22**, Scheme 5, route a), as the main product derived from the drug (>85–90 mol% with respect to the terpyridine fragment). The ^1^H and ^13^C resonances of the ethyl-CH_3_ were detected at 1.30 and 15.1 ppm, respectively, while those of the ethyl-CH_2_ were detected at 2.85 and 28.3 ppm, respectively. The structure was confirmed by the HMBC correlation between the methylene protons (2.85 ppm) and C3′/C5′ detected at 120.0 ppm, and correlation between the H3′/H5′ proton (8.33 ppm) and the methylene *C*H_2_ (28.3 ppm). The origin of the H^•^ abstracted by **21** (maybe from the R_1_R_2_NO^•^ fragment) was not investigated further. Beside 4′-ethyl terpyridine, all Fe(III)-heme protons were detected and assigned, similarly to the resonances of the starting heme, and with expected integral values. In particular, the four *meso* protons were detected as expected at 2.82, 1.21, 0.39, and −0.09 ppm, highly shielded due to the current cycle of this paramagnetic complex. The β-pyrrolic methyl substituents were detected as four singlets at 16.70, 16.27, 12.58, and 10.79 ppm; the vinyl C*H* were at 11.24 and 10.42 ppm, and the vinyl C*H*_2_ were at −1.72, −2.25, −2.35, and −2.82 ppm (4 × 1H); the propionate methylene were detected at 6.35, 5.97, −0.37, −1.70 ppm (4 × 2H). Thus, no modified porphyrin macrocycle could be detected. As in the case of (*RR*/*SS*)-**1a** and (*RS/SR*)-**1a**, no coupling product between heme and the drug could be detected.

Analysis of this reaction mixture by LC-MS indicated that (*RR*/*SS*)-**2F** and (*RS*/*SR*)-**2F** completely disappeared in these reaction conditions (t_R_ = 13.5 min). The major drug derived product was detected at 4.7 min, with *m/z* values of 262.3 amu (M + H^+^) and 284.3 (M + Na^+^), confirming the characterization of 4′-ethyl terpyridine **22** as the major product. Two other peaks were detected at 8.0 and 10.0 min, each of them with *m/z* = 521.3 (M + H^+^) and 543.3 amu (M + Na^+^). These *m/z* values, and the isotopic profiles were consistent with the dimerization of the alkyl radical generated by homolysis of the NO-C bond (Compound **23**, Scheme 5). The detection of two chromatographic peaks is consistent with the existence of two diastereomeric racemates ((*RR*/*SS*) and (*RS*/*SR*)) expected for compound **23**. These results clearly indicated that, upon homolysis of these alkoxyamines under argon, the generated alkyl radical **21** abstracted H^•^ to produce **22**, or alternatively dimerized to produce **23**, rather than to react with the porphyrin macrocycle.

When (*RS/SR*)-**2F** or (*RR*/*SS*)-**2F** was homolyzed in the presence of heme under an air atmosphere (Scheme 5, route b), NMR analysis of the total reaction mixture allowed the detection of two terpyridine derivatives substituted at C4′, **24** and **25**. The structure of the secondary alcohol **25** was assessed by the detection of CH_3_-*CH*(OH)- at 4.97 ppm in the proton, and 68.0 ppm in ^13^C-NMR, respectively. The C*H*_3_-CH(OH)- was detected at 1.45 ppm in the proton, and 25.4 ppm in ^13^C-NMR, respectively. HMBC correlations between the C*H*(OH) signal (4.97 ppm) and C3′/C5′ (117.7 ppm) on the one hand, and between the H3′/H5′ (8.53 ppm) and the *C*H(OH) (68.0 ppm) on the other hand, confirmed the structure. The 4′-acetyl terpyridine, **24**, was characterized by the carbonyl detected at 198.0 ppm, the methyl detected at 2.72 ppm and 26.8 ppm in ^1^H and ^13^C, respectively, and confirmed by the HMBC correlation of the carbonyl-C with H3′ and H5′ (8.87 ppm) and with C*H*_3_ (2.72 ppm). The other protons and carbons of compounds, **24** and **25**, were all detected, and were rather similar for both compounds. The alcohol **25** and ketone **24** derivatives are likely generated by the trapping of dioxygen by the secondary alkyl radical **21** to yield a peroxyl radical, followed by the dimerization of the peroxyl, and subsequent cleavage to the corresponding alkoxyl radical (Figure S16) [38]. The two products, **24** and **25**, were also generated by thermal homolysis of the alkoxyamine in the same conditions, but in the absence of heme.

The alkoxyamine **8F** contains the 4-hydroxy-tetramethylpiperidine-*N*-oxyl (**8**, Figure S1A) instead of the phosphonate derivative, **2**, associated with the 1-(4′-terpyridyl) radical **F**. Its antimalarial activity is the highest in the tested series, with an IC_50_ value of 0.14 µM on the *P. falciparum* line FcB1-Columbia, (~1 µM on the F32 strains) but a weak selectivity index of 0.5 (Table 1, Table S1). The reactivity of **8F** with heme was evaluated under aerobic conditions in DMSO-*d*_6_, in similar conditions as reported above for **2F**. Monitoring of the reaction for 3 h by LC-MS indicated that heme was not significatively modified, while 85–90% of the starting amount of **8F** disappeared. After the addition of KCN in the crude reaction mixture, NMR analysis allowed for the characterization of unchanged low spin Fe(III)-heme along with 4′-acetyl terpyridine (**24**, Scheme 5, chemical shifts of *CH*_3_-CO- at 2.78 and 27.7 ppm in ^1^H and ^13^C, respectively, *C*O at 198.4 ppm, HC3′/HC5′ at 8.83 and 118.2 ppm, 82% of the products containing the terpyridine moiety). Notably, the 4′-acetyl terpyridine was partly deuterated on the methyl group, giving rise to a -CH_2_D residue. This was assessed by the detection of the -C*H*_2_D at 2.76 ppm, as a triplet with ^2^*J*_HD_ = 1.8 Hz, that was correlated in HSQC with the resonance of CH_2_D at 27.7 ppm. The mole ratio **24**/**24*-d*** was 45/55. The mechanism of introduction of a deuterium atom from DMSO-*d*_6_ within 4′-acetyl terpyridine was not investigated further; however, this reaction clearly indicates that the alkyl radical resulting from the homolysis of the alkoxyamine NO-C bond does not efficiently target heme.

Two minor products containing the terpyridine residue were also identified: unreacted starting material **8F** (8%), and a product that was tentatively identified as **25**, Scheme 5 (chemical shifts of *CH*(OH) at 4.94 ppm and 67.8 ppm in ^1^H and ^13^C, respectively, C*H*_3_-CH(OH)- at 1.42 ppm, HC3′/HC5′ at 8.45 and 118.2 ppm, 10%). These results evidence that the alkoxyamine **8F** did not react with heme to produce heme-alkyl covalent adducts. This was confirmed by a diffusion ordered spectroscopy (DOSY) NMR analysis: the diffusion coefficients D were found to be (1.9 ± 0.1) × 10^−10^ m^2^/s, (2.6 ± 0.1) × 10^−10^ m^2^/s, and (2.1 ± 0.1) × 10^−10^ m^2^/s for **8F**, **24** and **25**, respectively, significantly higher than the (1.4 ± 0.1) × 10^−10^ m^2^/s value of D for heme, indicating that the reaction products of **8F** in the presence of heme did not contain the heme moiety.

**Scheme 6:**
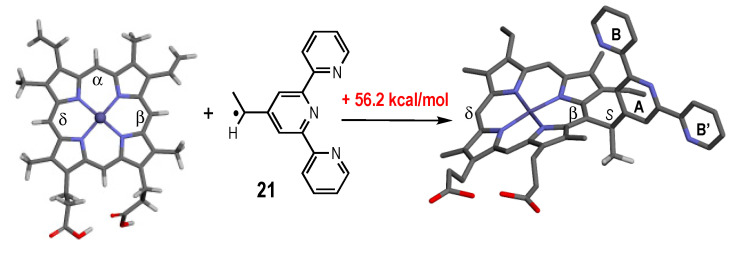
Computed free energy (B3lyp/3-21G*) of alkylation of heme at the β-*meso* position, by the alkyl radical derived from the homolysis of (*RR*/*SS*)-2F or (*RS/SR*)-2F.

Consequently, in aerobic conditions, the reaction of the alkyl radical **21** with dioxygen is expected to occur more quickly than with the porphyrin macrocycle of heme, a reaction that was not detected in vitro. In fact, the steric hindrance of the secondary alkyl radical **21** may prevent it from accessing the *meso* carbon of the heme cycle. Calculation by the B3lyp/3-21G* method of the alkylation free energy ΔG for the alkylation of heme by **21** was found to be +56.2 kcal/mol and +58.4 kcal/mol at Cβ and Cδ, respectively, indicating that the coupling between the two moieties is unfavorable (Scheme 5). In addition, in the computed heme-drug adduct, the heme residue is strongly distorted from planarity, which is the preferred geometry of the highly aromatic heme macrocycle, and the dihedral angle between two *trans* pyrrole cycles is between 147–149°, instead of ~180°, the normal value in a planar heme residue (Scheme 6).

Alkylation of heme within the parasite can therefore not be considered to be responsible for the antimalarial activity of alkoxyamines containing the alkyl radical **F**, namely (*RS/SR*)-**2F**, (*RR*/*SS*)-**2F**, or **8F**. Conversely, production of the alkyl radical in an oxygen rich biological medium may result in the generation of the peroxyl radical (Scheme 5, route b) which is able to induce damaging radical chain peroxidations. These non-selective peroxidations may be responsible for both antimalarial activity and cytotoxicity of these alkoxyamines, explaining a moderate selectivity index of these drugs (ratio Vero cells/*P. falciparum* = 1 or 0.5 for **2F** and **8F**, respectively, Table 1).

**Scheme 7:**
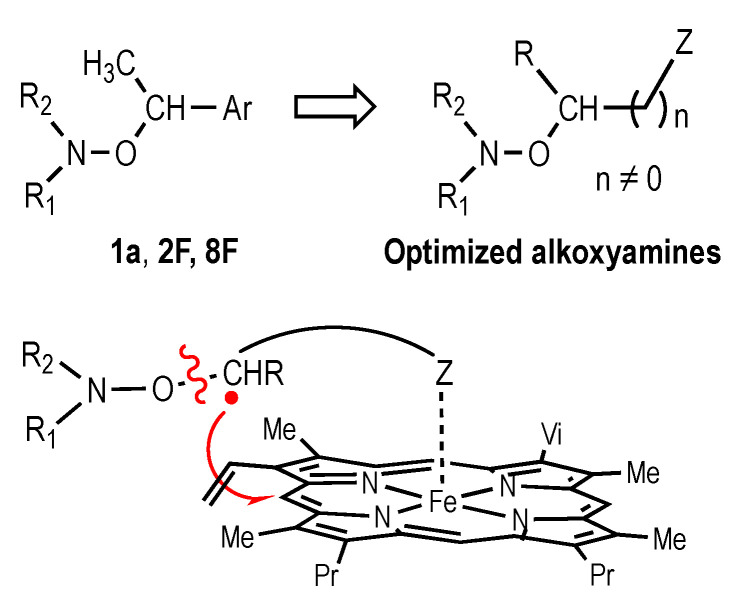
Optimized structure of alkoxyamines.

Moreover, the computational study indicates that the main steric hindrance preventing heme alkylation by the radical **21** was generated by cycle A of terpyridine (Scheme 6), while cycles B and B’ do not take a significant part in the steric hindrance. This suggests that the result should be similar with any alkoxyamine having an aryl substituent on the C (structure (R_1_,R_2_)-NO-C-Ar). The linker between the putative alkylating C^•^ **21** and the pyridine, terpyridine, or any aromatic group should be long enough (Scheme 7), to enable the radical to insert between the β-pyrrolic substituents and alkylate heme at *meso* carbons. In addition, if the Z substituent of alkoxyamine is able to behave as a ligand of the iron heme, it is expected to improve the selectivity of alkoxyamines for *Plasmodium*, and thus improve the selectivity index of the drugs. Such drugs are currently under investigation.

Notably, we focused here on the reactivity of the secondary alkyl radical generated by homolysis of the NO–C bond of alkoxyamines. This homolysis also produces the (R_1_,R_2_)-NO^•^ nitroxyl radical (=aminoxyl radical), which is expected to interfere with redox metabolism in vivo [39,40]. For instance, 4-Hydroxy-2,2,6,6-tetramethylpiperidine-N-oxyl (tempol) is reported to limit the formation of toxic hydroxyl radicals produced by Fenton reactions in cell and animal studies. When administered intravenously, tempol caused rapid and reversible dose-dependent reductions in blood pressure in rodent models of hypertension [41]. However, this issue is beyond the scope of the present report.

## 3. Conclusions

Among the series of alkoxyamines which have been evaluated against *P. falciparum* and *S. mansoni*, several of them exhibit significant activities (IC_50_ less than 500 nM against *Plasmodium* and mean time to kill the *S. mansoni* worms of 1 h). The antiparasitic activity of these drugs is probably not related to a hypothetical heme alkylation since such a pathway has not been evidenced in vitro. Even with the large set of data obtained with these alkoxyamines, it has not been possible to evidence a straightforward relationship between the structure and the activity of the drugs. However, both the desired antiparasitic activity and the cytotoxicity are likely to be due to similar radical pathways, thus leading to rather poor selectivity index values.

In order to separate antiparasitic activity and cytotoxicity, and consequently improve the selectivity of the drugs, it will be important to develop a new series of heme-targeting alkoxyamines that possess a chemical function able to activate the homolysis of the NO-C bond upon coordination on the heme iron. In addition, while coordinating onto the iron, these molecules are sterically designed to generate the C-centered radical in close vicinity of the alkylable heme C_meso_ positions (Scheme 6).

## 4. Materials and Methods

### 4.1. Synthesis of Alkoxyamines

#### 4.1.1. Methods

Analytical thin layer chromatographies (TLC) were carried out using Merck Kieselgel 60 F254 plates; spots were visualized upon exposure to UV light or stained with phosphomolybdic acid solution in EtOH and heat revealed. Purifications were performed on a Reveleris^®^ X2 flash chromatography system (BUCHI, Flawil, Switzerland) on Merck Kieselgel 60 (230–400 mesh). All experiments were performed in anhydrous conditions and under an inert atmosphere using argon and, except where stated, using dried apparatus and employing standard techniques for handling air-sensitive materials. ^1^H, ^13^C, and ^31^P-NMR spectra were recorded in CDCl_3_ on an Avance 300 Bruker spectrometer working at 300.13 MHz in proton. Chemical shifts (*δ*, ppm) were reported using residual non-deuterated solvents as the internal reference for ^1^H and ^13^C-NMR spectra, and using an internal capillary filled with 85% H_3_PO_4_ for ^31^P-NMR spectra.

The purity of final alkoxyamines (*RS/SR*)- and (*RR*/*SS*)-**2F**, -**4F**, -**6F**, -**2G**, -**6G**, and (*R/S*)-**8F**, was evaluated to be higher than 97–98 mol% because there was no other significant peak than those of the targeted compound in the ^1^H-NMR spectra recorded in the following conditions. Acquisition: 5–7 mg of alkoxyamine was dissolved in 0.5 mL of CDCl_3_; pulse program: zg30; sample temperature: 300 K ± 0.1 K; data points (acquired) = 32,768; zero-filling SI = 65,536; dummy scans: 2–4; scans NS = 16–32; pulse width P1 = 14.0 µs; relaxation delay D1 = 1 s; acquisition time AQ = 5.45 s; spectral window SW = 6009.6 Hz; transmitter offset = 300.13 MHz; pre-acquisition delay DE = 10.0 µs. Processing: line broadening LB = 0–0.3 Hz; automatic phasing; 5th order polynomial baseline correction. The spectra are provided as Supporting Information.

High-resolution mass spectra (HRMS) were recorded on a SYNAPT G2 HDMS (Waters) spectrometer equipped with a pneumatically assisted atmospheric pressure ionization source (API). Positive mode electrospray ionization was used on samples: electrospray voltage (ISV): 2800 V; opening voltage (OR): 20 V; nebulizer gas pressure (nitrogen): 800 L h^−1^.

#### 4.1.2. Syntheses and Characterization

*4′-(1-Bromoethyl)-2,2′:6′,2′′-terpyridine* (**18**): A mixture of 4′-vinylterpyridine (1.40 g, 5.40 mmol) and palladium on activated carbon (60 mg, 0.57 mmol, 0.1 eq.) in ethyl acetate (20 mL) was stirred at room temperature for 4 h under hydrogen atmosphere. The reaction mixture was filtered and concentrated under reduced pressure to give compound 4′-ethyl-2,2′:6′,2′′-terpyridine, which was used in the next step without any further purification (1.36 g, 96%). ^1^H-NMR (300 MHz, CDCl_3_): *δ* 8.69 (d, *J* = 4.7 Hz, 2H); 8.61 (d, *J* = 8.0 Hz, 2H); 8.32 (s, 2H); 7.83 (td, *J* = 7.7, 1.6 Hz, 2H); 7.32–7.28 (m, 2H); 2.83 (q, *J* = 7.6 Hz, 2H); 1.37 (t, *J* = 7.6 Hz, 3H).^13^C-NMR (75 MHz, CDCl_3_): *δ* 156.5; 155.3; 155.1; 149.0; 136.7; 123.6; 121.3; 120.6; 28.7; 14.5.

4′-Ethyl-2,2′:6′,2′′-terpyridine (1.00 g, 3.83 mmol), *N*-bromosuccinimide (0.75 g, 4.21 mmol, 1.1 eq.) and benzoyl peroxide (93 mg, 0.38 mmol, 0.1 eq.) were dissolved in CCl_4_ (20 mL) and refluxed for 6 h. After cooling, the reaction mixture was subjected to filtration. The filtrate was washed with saturated aqueous solution of sodium bicarbonate and saturated aqueous solution of sodium chloride, dried over magnesium sulfate and concentrated under reduced pressure. The crude product was chromatographed on silica gel deactivated by 10% TEA using a 9/1: petroleum ether/ethyl acetate mixture as the eluting solvent. Removal of the solvent under reduced pressure yielded 4′-(1-bromoethyl)-2,2′:6′,2′′-terpyridine **18** as the white solid (0.71 g, 55%). ^1^H-NMR (300 MHz, CDCl_3_): *δ* 8.60 (d, *J* = 4.1 Hz, 2H); 8.49 (d, *J* = 8.0 Hz, 2H); 8.42 (s, 2H); 7.73 (td, *J* = 7.8, 1.7 Hz, 2H); 7.24–7.19 (m, 2H); 5.17 (q, *J* = 6.9 Hz, 1H); 2.05 (d, *J* = 6.9 Hz, 2H). ^13^C-NMR (101 MHz, CDCl_3_): *δ* 156.1; 155.8; 153.7; 149.1; 136.9; 124.0; 121.4; 119.0; 46.8; 26.1. HRMS (ESI) cal. for C_17_H_15_N_3_Br^+^: 340.0444 [M + H]^+^; found: 340.0441.

*Diethyl(1-((1-([2,2′:6′,2′′-terpyridin]-4′-yl)ethoxy)(1-((tert-butyldimethylsilyl)oxy)-2-methylpropan-2-yl)amino)-2,2-dimethylpropyl)phosphonate* ((*RR*/*SS*)-**6F** *and* (*RS*/*SR*)-**6F**)): To a stirred suspension of CuBr (0.43 g, 2.94 mmol, 1.0 eq.) and Cu (0.37 g, 5.88 mmol, 2.0 eq.) in degassed benzene (10 mL) was added N,N,N’,N”,N”-pentamethyldiethylenetriamine (0.61 mL, 2.94 mmol, 1.0 eq.). The resulting mixture was stirred under argon at room temperature for 10 min, then a solution of the bromide **18** (1.00 g, 2.94 mmol) and SG1-OTBS (1.37 g, 3.23 mmol, 1.1 eq.) in degassed benzene (10 mL) was slowly added. The mixture was stirred overnight under argon (15 h). It was then diluted with DCM, quenched and washed several times with saturated aqueous ammonia solution, water and brine. After drying with MgSO_4_, filtration and concentration, the crude product was chromatographed on silica gel deactivated by 10% TEA using a 15/85: petroleum ether/diethyl ether mixture as the eluting solvent to give the major product (*RS*/*SR*)-**6F** (1.10 g) and the minor product (*RR*/*SS*)-**6F** (510 mg) as a white solid (75%). (*RS*/*SR*)-**6F**: ^31^P-NMR (162 MHz, CDCl_3_): δ 24.67. ^1^H-NMR (300 MHz, CDCl_3_): δ 8.68 (dd, *J* = 4.8, 0.8 Hz, 2H); 8.58 (d, *J* = 8.0 Hz, 2H); 8.49 (s, 2H); 7.83 (td, *J* = 7.8, 1.8 Hz, 2H); 7.30 (ddd, *J* = 7.5, 4.8, 1.1 Hz, 2H); 5.42 (q, *J* = 6.5 Hz, 1H); 4.00–3.85 (m, 2H); 3.79 (d, *J* = 26.8 Hz, 3H); 3.72–3.61 (m, 2H); 1.66 (d, *J* = 6.6 Hz, 3H); 1.26 (s, 6H); 1.24 (s, 9H), 1.21 (d, *J* = 7.1 Hz, 3H); 0.95–0.90 (m, 12H); 0.11 (s, 6H). ^13^C-NMR (75 MHz, CDCl_3_): δ 156.3; 155.4; 154.7; 149.1; 136.6; 123.5; 121.1; 119.3; 69.6 (d, *J* = 138.8 Hz); 69.0; 65.5; 61.8 (d, *J* = 6.7 Hz); 59.2 (d, *J* = 7.6 Hz); 35.5 (d, *J* = 5.1 Hz); 30.6 (d, *J* = 5.9 Hz); 25.9; 23.8; 22.5; 21.6; 18.3; 16.3 (d, *J* = 5.8 Hz); 16.1 (d, *J* = 7.0 Hz); −5.4; −5.5. HRMS (ESI) cal. for C_36_H_58_N_4_O_5_PSi^+^: 685.3909 [M + H]^+^; found: 685.3909. (*RR*/*SS*)-**6F**: ^31^P-NMR (162 MHz, CDCl_3_): δ 25.99. ^1^H-NMR (300 MHz, CDCl_3_): δ 8.69 (dd, *J* = 4.6, 0.7 Hz, 2H); 8.62 (d, *J* = 8.0 Hz, 2H); 8.44 (s, 2H); 7.85 (td, *J* = 7.7, 1.8 Hz, 2H); 7.32 (ddd, *J* = 7.4, 4.8, 1.0 Hz, 2H); 5.20 (q, *J* = 6.6 Hz, 1H); 4.53–4.33 (m, 2H); 4.16–3.97 (m, 2H); 3.73 (d, *J* = 26.2 Hz, 1H); 3.22 (q, *J* = 9.9 Hz, 2H); 1.70 (d, *J* = 6.8 Hz, 3H); 1.47 (t, *J* = 7.1 Hz, 3H); 1.33 (t, *J* = 7.1 Hz, 3H); 1.26 (s, 9H); 0.95 (s, 3H); 0.93 (s, 3H), 0.73 (s, 9H); −0.28 (s, 3H); −0.30 (s, 3H). ^13^C-NMR (75 MHz, CDCl_3_): δ 156.6; 156.2; 155.6; 149.1; 136.7; 123.6; 121.1; 118.8; 70.6; 69.7 (d, *J* = 139.0 Hz); 65.6; 62.0 (d, *J* = 6.3 Hz); 58.9 (d, *J* = 7.5 Hz); 35.8 (d, *J* = 6.0 Hz); 29.8 (d, *J* = 5.8 Hz); 25.7; 24.8; 23.9; 22.7; 18.0; 16.8 (d, *J* = 5.5 Hz); 16.2 (d, *J* = 6.9 Hz); −5.8; −5.9. HRMS (ESI) calculated for C_36_H_58_N_4_O_5_PSi^+^: 685.3909 [M + H]^+^; found: 685.3910.

*Diethyl(1-((1-([2,2′:6′,2′′-terpyridin]-4′-yl)ethoxy)(1-hydroxy-2-methylpropan-2-yl)amino)-2,2-dimethylpropyl)phosphonate* (*RS*/*SR*)-**2F**): To a solution of compound (*RS*/*SR*)-**6F** (0.50 g, 0.73 mmol) in dry THF (10 mL) was added HF/Py (0.50 mL) at 0 ^°^C, and the mixture was stirred at room temperature for 12 h. The reaction was quenched by the addition of a saturated solution of NaHCO_3_ (20 mL) to pH 8, extracted with DCM and washed with water and brine. The combined organic phase was dried with MgSO_4_ and then concentrated under reduced pressure. The crude product was chromatographed on silica gel deactivated by 10% TEA using a 10% ethyl acetate in a diethyl ether mixture as the eluting solvent. Removal of the solvent under reduced pressure yielded compound (*RS*/*SR*)-**2F** as the white solid (360 mg, 85%). (*RS*/*SR*)-**2F**: ^31^P-NMR (162 MHz, CDCl_3_): δ 27.09. ^1^H-NMR (300 MHz, CDCl_3_): δ 8.66 (d, *J* = 4.1 Hz, 2H); 8.54 (d, *J* = 7.9 Hz, 2H); 8.41 (s, 2H); 7.78 (t, *J* = 7.3 Hz, 2H); 7.30–7.22 (m, 2H); 5.23 (q, *J* = 6.5 Hz, 1H); 4.06–3.80 (m, 5H); 3.63–3.42 (m, 2H); 1.62 (d, *J* = 6.5 Hz, 3H); 1.30 (s, 3H); 1.18–1.12 (m, 15H); 0.95 (t, *J* = 7.0 Hz, 3H). ^13^C-NMR (75 MHz, CDCl_3_): δ 156.2; 155.4; 154.6; 149.2; 136.7; 123.6; 121.3; 118.6; 69.3 (d, *J* = 134.8 Hz); 67.4; 64.7; 62.1 (d, *J* = 6.5 Hz); 61.1 (d, *J* = 8.0 Hz); 35.3 (d, *J* = 3.7 Hz); 30.6 (d, *J* = 5.5 Hz); 26.6; 24.7; 21.9; 16.3 (d, *J* = 5.6 Hz); 15.9 (d, *J* = 6.4 Hz). HRMS (ESI) calculated for C_30_H_44_N_4_O_5_P^+^: 571.3044 [M + H]^+^; found: 571.3047.

Starting from (*RR*/*SS*)-**6F** (0.51 g, 0.25 mmol), derivative (*RR*/*SS*)-**2F** (366 mg, 85%) was prepared according to the same procedure used for the preparation of (*RS*/*SR*)-**2F**. (*RR*/*SS*)-**2F**: ^31^P-NMR (162 MHz, CDCl_3_): *δ* 27.20. ^1^H-NMR (300 MHz, CDCl_3_): *δ* 8.64 (d, *J* = 3.9 Hz, 2H); 8.55 (d, *J* = 7.9 Hz, 2H); 8.37 (s, 2H); 7.78 (t, *J* = 7.5 Hz, 2H); 7.29 – 7.23 (m, 2H); 5.10 (q, *J* = 6.4 Hz, 1H); 4.26–4.10 (m, 4H); 3.74–3.61 (m, 2H); 3.03 (d, *J* = 12.3 Hz, 1H); 1.60 (d, *J* = 6.6 Hz, 3H); 1.35 (t, *J* = 7.2 Hz, 3H); 1.32 (t, *J* = 7.1 Hz, 3H); 1.19 (s, 3H); 1.16 (s, 9H); 1.01 (s, 3H). ^13^C-NMR (75 MHz, CDCl_3_): *δ* 156.2; 156.1; 155.6; 149.2; 136.7; 123.7; 121.2; 118.7; 85.5; 69.4 (d, *J* = 137.8 Hz); 67.0; 65.1; 62.2 (d, *J* = 6.4 Hz); 60.6 (d, *J* = 8.0 Hz); 35.7 (d, *J* = 4.7 Hz); 30.0 (d, *J* = 5.5 Hz); 27.3; 24.5; 24.1; 16.5 (d, *J* = 5.6 Hz); 16.3 (d, *J* = 6.6 Hz). HRMS (ESI) calculated for C_30_H_44_O_5_P^+^: 571.3044 [M + H]^+^; found: 571.3044.

*Diethyl(1-((1-([2,2′:6′,2′′-terpyridin]-4′-yl)ethoxy)(2-methyl-1-oxopropan-2-yl)amino)-2,2-dimethylpropyl)phosphonate* (*RS*/*SR*)-**7F**): A mixture of compound (*RS*/*SR*)-**2F** (200 mg, 0.35 mmol), Dess–Martin reagent (230 mg, 0.53 mmol, 1.5 eq.) and sodium bicarbonate (0.15 g, 1.75 mmol, 5.0 eq.) in dry CH_2_Cl_2_ (20 mL) was stirred at 0 °C for 4 h. The reaction mixture was then diluted with 10% Na_2_S_2_O_3_ solution (10 mL) and then DCM-extracted. The combined organic layers were washed with water and brine, dried by MgSO_4_ and concentrated under reduced pressure. The crude product was chromatographed on silica gel deactivated by 10% TEA using a 1/5 ethyl acetate/diethyl ether mixture as the eluting solvent. Removal of the solvent under reduced pressure yielded the corresponding aldehyde as a white solid (170 mg, 85%). (*RS*/*SR*)-**7F**): ^31^P-NMR (162 MHz, CDCl_3_): δ 23.14. ^1^H-NMR (300 MHz, CDCl_3_): δ 9.90 (s, 1H); 8.65 (d, *J* = 4.2 Hz, 2H); 8.52 (d, *J* = 7.9 Hz, 2H); 8.38 (s, 2H); 7.78 (td, *J* = 7.8, 1.5 Hz, 2H); 7.30–7.21 (m, 2H); 5.38 (q, *J* = 6.4 Hz, 1H); 3.94–3.85 (m, 2H); 3.71–3.63 (m, 2H); 3.41 (d, *J* = 26.5 Hz, 1H); 1.64 (d, *J* = 6.5 Hz, 3H); 1.33 (s, 3H); 1.31 (s, 3H); 1.18–1.13 (m, 12H); 0.92 (t, *J* = 7.1 Hz, 3H). ^13^C-NMR (75 MHz, CDCl_3_): δ 202.6; 156.6; 155.5; 153.7; 149.1; 136.7; 123.6; 121.2; 119.1; 78.4; 71.0; 69.5 (d, *J* = 140.3 Hz); 62.0 (d, *J* = 6.9 Hz); 60.1 (d, *J* = 7.6 Hz); 35.4 (d, *J* = 4.8 Hz); 30.4 (d, *J* = 5.8 Hz); 22.8; 21.8; 20.1; 16.2 (d, *J* = 5.4 Hz); 16.1 (d, *J* = 6.3 Hz). HRMS (ESI) calculated for C_30_H_42_N_4_O_5_P^+^: 569.2887 [M + H]^+^; found: 569.2891.

Starting from (*RR*/*SS*)-**2F** (201 mg, 0.25 mmol), (*RR*/*SS*)-**7F** aldehyde derivative (171 mg, 85%) was prepared according to the same procedure used for the preparation of (*RS/SR*)-**7F**. ^31^P-NMR (162 MHz, CDCl_3_): *δ* 24.22. ^1^H-NMR (300 MHz, CDCl_3_): *δ* 9.36 (s, 1H), 8.69 (d, *J* = 4.7 Hz, 2H), 8.63 (d, *J* = 8.0 Hz, 2H), 8.39 (s, 2H), 7.84 (td, *J* = 7.8, 1.7 Hz, 2H), 7.34–7.29 (m, 2H), 5.30 (q, *J* = 6.7 Hz, 1H), 4.49–4.21 (m, 2H), 4.20–3.99 (m, 2H), 3.34 (d, *J* = 25.5 Hz, 1H), 1.68 (d, *J* = 6.8 Hz, 3H), 1.45 (t, *J* = 7.1 Hz, 3H), 1.33 (t, *J* = 7.1 Hz, 3H), 1.27 (s, 9H), 1.17 (s, 3H), 0.93 (s, 3H). ^13^C-NMR (75 MHz, CDCl_3_): *δ* 202.5; 156.1; 155.7; 155.0; 149.1; 136.8; 123.7; 121.3; 118.9; 84.9; 71.5; 69.4 (d, *J* = 140.8 Hz); 62.2 (d, *J* = 6.5 Hz); 59.6 (d, *J* = 7.4 Hz); 35.7 (d, *J* = 5.5 Hz); 29.7 (d, *J* = 5.7 Hz); 23.8; 23.1; 19.6; 16.7 (d, *J* = 5.5 Hz); 16.3 (d, *J* = 6.6 Hz). HRMS (ESI) calculated for C_30_H_42_N_4_O_5_P^+^: 569.2887 [M + H]^+^; found: 569.2888.

*2-((1-([2,2′:6′,2′′-Terpyridin]-4′-yl)ethoxy)(1-(diethoxyphosphoryl)-2,2-dimethylpropyl)amino)-2-methylpropanoic acid* (*RS*/*SR*)-**4F**: A mixture of the aldehyde (*RS*/*SR*)-**7F** (150 mg, 0.26 mmol), sodium chlorite (0.12 g, 5.0 eq.), sodium dihydrogen phosphate (250 mg, 8.0 eq.) and 2-methylbut-2-ene (0.10 mL) in *t*-BuOH (10 mL) was stirred at room temperature for 2 h. The reaction mixture was then diluted with water and then extracted by DCM. The combined organic layers were washed with water and brine, dried by MgSO_4_ and concentrated under reduced pressure. The crude product was chromatographed on silica gel deactivated by 10% TEA using DCM with 10% MeOH as the eluting solvent. After removing the solvent, the TEA salt form was obtained. Then, using 1 eq of trifluoroacetic acid (TFA), the salt was transferred to its acid form (*RS*/*SR*)-4F as a white solid (131 mg, 80%). (*RS*/*SR*)-**4F**: ^31^P-NMR (162 MHz, CDCl_3_): δ 27.28. ^1^H-NMR (300 MHz, CDCl_3_): δ 8.70 (d, *J* = 4.2 Hz, 2H); 8.59 (d, *J* = 7.9 Hz, 2H); 8.40 (s, 1H); 7.84 (t, *J* = 7.7 Hz, 2H); 7.34 – 7.28 (m, 2H); 5.22 (q, *J* = 6.4 Hz, 1H); 4.19–3.92 (m, 4H); 3.44 (d, *J* = 25.1 Hz, 1H); 1.65 (d, *J* = 6.6 Hz, 3H); 1.60 (s, 3H); 1.50 (s, 3H); 1.28 (t, *J* = 7.0 Hz, 3H); 1.10 (s, 9H); 1.05 (t, *J* = 7.0 Hz, 3H). ^13^C-NMR (75 MHz, CDCl_3_): δ 175.6; 156.1; 155.4; 153.9; 149.1; 136.8; 123.7; 121.4; 119.1; 81.1; 70.1; 69.0 (d, *J* = 139.7 Hz); 62.4 (d, *J* = 7.0 Hz); 61.5 (d, *J* = 7.9 Hz); 35.2 (d, *J* = 5.4 Hz); 30.0 (d, *J* = 5.8 Hz); 26.2; 25.9; 22.6; 16.3 (d, *J* = 5.6 Hz); 16.0 (d, *J* = 6.4 Hz). HRMS (ESI) calculated for C_30_H_42_N_4_O_6_P^+^: 585.2837 [M + H]^+^; found: 585.2836.

Starting from (*RR*/*SS*)-**2F** (149 mg, 0.26 mmol), derivative (*RR*/*SS*)-4F (132 mg, 80%) was prepared according to the same procedure used for the preparation of (*RS/SR*)-**4F**. ^31^P-NMR (162 MHz, CDCl_3_): *δ* 26.25. ^1^H-NMR (300 MHz, CDCl_3_): *δ* 8.67 (d, *J* = 4.5 Hz, 2H); 8.54 (d, *J* = 7.9 Hz, 2H); 8.50 (s, 2H); 7.79 (t, *J* = 7.7 Hz, 2H); 7.29–7.23 (m, 2H); 5.65 (q, *J* = 6.7 Hz, 1H); 4.60–4.44 (m, 2H); 4.27–4.09 (m, 2H); 3.47 (d, *J* = 24.8 Hz, 1H); 1.82 (d, *J* = 6.7 Hz, 3H); 1.58 (t, *J* = 7.0 Hz, 3H); 1.55 (s, 3H); 1.42 (t, *J* = 7.1 Hz, 3H); 1.37–1.32 (m, 12H). ^13^C-NMR (75 MHz, CDCl_3_): *δ* 176.6, 155.9, 155.8, 154.8, 148.8, 136.9, 123.6, 121.5, 119.0, 84.6, 69.4, 68.7 (d, *J* = 139.7 Hz), 62.3 (d, *J* = 6.5 Hz), 59.8 (d, *J* = 7.5 Hz), 35.9 (d, *J* = 5.8 Hz), 29.7 (d, *J* = 5.7 Hz), 27.2, 25.0, 21.1, 16.7 (d, *J* = 5.6 Hz), 16.3 (d, *J* = 6.5 Hz). HRMS (ESI) calculated for C_30_H_42_N_4_O_6_P^+^: 585.2837 [M + H]^+^; found: 585.2838.

*1-(1-([2,2′:6′,2′′-Terpyridin]-4′-yl)ethoxy)-2,2,6,6-tetramethylpiperidin-4-ol* (*R/S*)-**8F**: To a stirred suspension of CuBr (0.21 g, 1.47 mmol, 1.0 eq.) and Cu (0.19 g, 2.94 mmol, 2.0 eq.) in degassed benzene (10 mL) was added *N,N,N’,N”,N”-*pentamethyldiethylenetriamine (0.3 mL, 1.47 mmol, 1.0 eq.). The resulting mixture was stirred under argon atmosphere at room temperature for 10 min, then a solution of the bromide compound **18** (0.50 g, 1.47 mmol) and 4-Hydroxy-TEMPO (0.30 g, 1.76 mmol, 1.2 eq.) in degassed benzene (10 mL) was slowly added. The mixture was stirred overnight under argon atmosphere (15 h). The reaction mixture was then diluted with DCM, quenched and washed several times with saturated aqueous ammonia solution, water and brine. After drying with MgSO_4_, filtration and concentration, the crude product was chromatographed on silica gel deactivated by 10% TEA using a 40/60: petroleum ether/diethyl ether mixture as the eluting solvent to give (*R/S*)-**8F** as a white solid (0.43 g, 68%). ^1^H-NMR (300 MHz, CDCl_3_): *δ* 8.71 (ddd, *J* = 4.8, 1.7, 0.8 Hz, 2H); 8.63 (d, *J* = 8.0 Hz, 2H); 8.42 (s, 2H); 7.84 (td, *J* = 7.8, 1.8 Hz, 2H); 7.32 (ddd, *J* = 7.5, 4.8, 1.1 Hz, 2H); 4.98 (q, *J* = 6.7 Hz, 1H); 3.97–3.89 (m, 1H); 1.84 (dt, *J* = 12.3, 3.6 Hz, 1H); 1.68 (dt, *J* = 12.4, 3.9 Hz, 1H); 1.59 (d, *J* = 6.7 Hz, 3H); 1.53–1.45 (m, 2H); 1.36 (s, 3H); 1.26 (s, 3H); 1.12 (s, 3H); 0.76 (s, 3H). ^13^C-NMR (75 MHz, CDCl_3_): *δ* 156.4; 156.3; 155.4; 149.1; 136.8; 123.7; 121.3; 118.9; 83.1; 63.2; 60.3; 60.1; 48.8; 34.6; 34.5; 30.3; 23.3; 21.4. HRMS (ESI) calculated for C_26_H_33_N_4_O_2_^+^: 433.2598 [M + H]^+^; found: 433.2593.

*5′-Vinyl-1,1′:3′,1′′-terphenyl***20**: A mixture of 5′-bromo-*m*-terphenyl 19 (1.00 g, 3.23 mmol), tributyl(vinyl)tin (1.23 mL, 4.2 mmol, 1.3 eq.), TEA (1.23 mL, 9.7 mmol, 3.0 eq.), and bis(triphenylphosphine)palladium dichloride (0.11 g, 0.16 mmol, 0.05 eq.) in DMF (20 mL) was stirred at 90 °C for 12 h under argon. The reaction mixture was then diluted with ice water (100 mL), stirred for 1h and then extracted by ethyl acetate. The combined organic layers were washed with water, dried by MgSO_4_ and concentrated under reduced pressure. The crude product was chromatographed on silica gel using a 5% ethyl acetate in petroleum ether as the eluting solvent. Removal of the solvent under reduced pressure yielded vinyl derivative **20** as a white solid (670 mg, 80%). ^1^H-NMR (300 MHz, CDCl_3_): *δ* 7.74–7.59 (m, 7H); 7.52–7.44 (m, 4H); 7.43–7.35 (m, 2H); 6.87 (dd, *J* = 17.6, 10.9 Hz, 1H); 5.90 (d, *J* = 17.6 Hz, 1H); 5.36 (d, *J* = 10.9 Hz, 1H). ^13^C-NMR (75 MHz, CDCl_3_): *δ* 142.14; 141.11; 138.53; 136.79; 128.82; 127.52; 127.31; 125.80; 124.13; 114.59. HRMS (ESI) calculated for C_20_H_26_Ag^+^: 363.0297 [M + Ag]^+^; found: 363.0298.

*Diethyl(1-((1-([1,1′:3′,1′′-terphenyl]-5′-yl)ethoxy)(1-((tert-butyldimethylsilyl)oxy)-2-methylpropan-2-yl)amino)-2,2-dimethylpropyl)phosphonate* (*RR*/*SS*)-**6G** and (*RS*/*SR*)-**6G**: To a stirred solution of Salen ligand (63 mg, 0.23 mmol, 0.1 eq. mol) in EtOH (10 mL) was added MnCl_2_·4H_2_O (46 mg, 0.23 mmol, 0.1 eq.) in an opened flask. After 30 min of stirring at room temperature, a solution of SG1-OTBS (2.00 g, 4.68 mmol, 2.0 eq.) and 5′-vinyl-1,1′:3′,1′′-terphenyl 20 (0.60 g, 2.34 mmol) in EtOH (20 mL) was added, followed by solid NaBH_4_ (0.18 g, 4.68 mmol, 2.0 eq.) added in small portions. The resulting suspension was stirred at room temperature overnight (15 h). After checking the reaction for completion by TLC (0.5 eq. of NaBH_4_ can be added in case it does not reach completion), EtOH was removed and ice water was added. The reaction mixture was then DCM-extracted 3 times. The layers were separated, and the organic phase was washed with water, brine and dried with MgSO_4_. After concentration under reduced pressure, the residue was purified by column chromatography with 10% ethyl acetate in petroleum ether as the eluting solvent to afford 1.12 g of 2 diastereoisomer as a colorless oil: 0.78 g of (*RS*/*SR*)-**6G** and 0.34 g of the (*RR*/*SS*)-**6G**. (*RS*/*SR*)-**6G**: ^31^P-NMR (162 MHz, CDCl_3_): δ 24.99. ^1^H-NMR (300 MHz, CDCl_3_): δ 7.71–7.66 (m, 7H); 7.48–7.43 (m, 4H); 7.38–7.34 (m, 2H); 5.42 (q, *J* = 6.4 Hz, 1H); 4.04–3.86 (m, 2H); 3.80 (d, *J* = 26.2 Hz, 1H); 3.73–3.32 (m, 4H); 1.66 (d, *J* = 6.5 Hz, 3H); 1.26 (s, 9H); 1.24–1.21 (m, 9H); 0.94–0.91 (m, 12H); 0.07 (s, 6H). ^13^C-NMR (75 MHz, CDCl_3_): δ 144.4; 141.4; 141.3; 128.8; 127.3; 127.3; 125.6; 125.2; 78.5; 70.1 (d, *J* = 139.0 Hz); 69.1; 65.3; 61.8 (d, *J* = 6.5 Hz); 58.8 (d, *J* = 7.6 Hz); 35.5 (d, *J* = 5.3 Hz); 30.5 (d, *J* = 6.0 Hz); 25.9; 23.6; 22.6; 21.3; 18.3; 16.4 (d, *J* = 5.6 Hz); 16.2 (d, *J* = 7.1 Hz); −5.3; −5.4. HRMS (ESI) calculated for C_39_H_61_NO_5_PSi^+^: 682.4051 [M + H]^+^; found: 682.4050. (*RR*/*SS*)-**6G**: ^31^P-NMR (162 MHz, CDCl_3_): δ 26.15. ^1^H-NMR (300 MHz, CDCl_3_): δ 7.70–7.64 (m, 5H); 7.51–7.44 (m, 6H); 7.40–7.35 (m, 2H); 5.12 (q, *J* = 6.5 Hz, 1H); 4.48–4.36 (m, 1H); 4.28–3.95 (m, 3H); 3.76 (d, *J* = 26.4 Hz, 1H); 3.21 (br.s, 2H); 1.69 (d, *J* = 6.7 Hz, 1H); 1.37 (t, *J* = 7.0 Hz, 3H); 1.32 (t, *J* = 7.1 Hz, 3H); 1.28 (s, 9H); 0.98 (s, 3H); 0.97 (s, 3H); 0.78 (s, 9H); -0.23 (s, 3H); –0.24 (s, 3H). ^13^C-NMR (75 MHz, CDCl_3_): δ 146.9; 141.7; 141.2; 128.8; 127.4; 127.2; 124.9; 124.5; 86.0; 69.8; 69.8 (d, *J* = 138.5 Hz); 65.5; 61.8 (d, *J* = 6.3 Hz); 58.9 (d, *J* = 7.0 Hz); 35.8 (d, *J* = 6.1 Hz); 30.0 (d, *J* = 5.8 Hz); 25.8; 25.1; 23.8; 22.3; 18.0; 16.8 (d, *J* = 5.6 Hz); 16.3 (d, *J* = 6.8 Hz); −5.6; −5.7. HRMS (ESI) calculated for C_39_H_61_NO_5_PSi^+^: 682.4051 [M + H]^+^; found: 682.4047.

*Diethyl(1-((1-([1,1′:3′,1′′-terphenyl]-5′-yl)ethoxy)(1-hydroxy-2-methylpropan-2-yl)amino)-2,2-dimethylpropyl)phosphonate* (*RS*/SR)-**2G**: To a solution of compound (*RS*/SR)-**6G** (0.50 g, 0.73 mmol) in dry THF (10 mL) was added HF/Py (0.5 mL) at 0 °C, and the mixture was stirred at room temperature for 12 h. The reaction was quenched by the addition of a saturated solution of NaHCO_3_ (20 mL) to pH 8, extracted with DCM and washed with water, 1M HCl solution and brine. The combined organic phase was dried with MgSO_4_ and then concentrated under reduced pressure. The crude product was chromatographed on silica gel using a 30% ethyl acetate in petroleum ether mixture as the eluting solvent. Removal of the solvent under reduced pressure yielded compound (*RS*/SR)-**2G** as a white solid (0.35 g, 85%). (*RS*/SR)-**2G**: ^31^P-NMR (162 MHz, CDCl_3_): δ 26.99. ^1^H-NMR (300 MHz, CDCl_3_): δ 7.69–7.64 (m, 7H); 7.48–7.43 (m, 4H); 7.38–7.33 (m, 2H); 5.25 (q, *J* = 6.4 Hz, 1H); 4.07–3.79 (m, 5H); 3.68–3.54 (m, 2H); 1.65 (d, *J* = 6.5 Hz, 3H); 1.33 (s, 3H); 1.23–1.15 (m, 15H); 1.01 (t, *J* = 7.0 Hz, 3H). ^13^C-NMR (75 MHz, CDCl_3_): δ 144.8; 141.5; 141.2; 128.8; 127.4; 127.2; 124.8; 124.2; 78.3; 69.7 (d, *J* = 135.7 Hz); 67.8; 64.8; 62.1 (d, *J* = 6.5 Hz); 60.8 (d, *J* = 7.9 Hz); 35.4 (d, *J* = 3.9 Hz); 30.7 (d, *J* = 5.6 Hz); 26.5; 24.6; 22.3; 16.7 (d, *J* = 5.6 Hz); 15.9 (d, *J* = 6.7 Hz). HRMS (ESI) calculated for C_33_H_47_NO_5_P^+^: 568.3186 [M + H]^+^; found: 568.3186.

Starting from (*RR*/*SS*)-**6G** (0,51 g, 0.25 mmol), derivative (*RR*/*SS*)-**2G** (366 mg, 85%) was prepared according to the same procedure used in the preparation of (*RS/SR*)-**2G**. ^31^P-NMR (162 MHz, CDCl_3_): *δ* 27.16. ^1^H-NMR (300 MHz, CDCl_3_): *δ* 7.72–7.63 (m, 5H); 7.55 (s, 2H); 7.49–7.44 (m, 4H); 7.39–7.34 (m, 2H); 5.09 (q, *J* = 6.4 Hz, 1H); 4.36–4.08 (m, 5H); 3.70 (d, *J* = 26.1 Hz, 1H); 3.64 (d, *J* = 9.4 Hz, 1H); 3.16 (d, *J* = 12.1 Hz, 1H); 1.64 (d, *J* = 6.6 Hz, 3H); 1.37 (t, *J* = 7.1 Hz, 6H); 1.25 (s, 9H); 1.08 (s, 3H); 0.83 (br.s, 3H). ^13^C-NMR (75 MHz, CDCl_3_): *δ* 146.1; 141.7; 141.1; 128.8; 127.4; 127.3; 125.1; 124.6; 85.3; 69.5 (d, *J* = 135.8 Hz); 67.3; 65.0; 61.9 (d, *J* = 6.5 Hz); 60.5 (d, *J* = 7.7 Hz); 35.7 (d, *J* = 4.6 Hz); 30.2 (d, *J* = 5.2 Hz); 27.1; 24.4; 23.7; 16.6 (d, *J* = 5.7 Hz); 16.3 (d, *J* = 6.6 Hz). HRMS (ESI) calculated for C_33_H_47_NO_5_P^+^: 568.3186 [M + H]^+^; found: 568.3184.

### 4.2. Evaluation against P. falciparum

#### 4.2.1. Parasite Culture

F32-ART5, an artemisinin-resistant strain and F32-TEM, an artemisinin-sensitive strain, were obtained from the parental clone F32-Tanzania, respectively under regular drug pressure cycles of increasing artemisinin concentrations for 5 years and without any artemisinin pressure [42]. After these drug pressure cycles allowing selection of the F32-ART5 strain, regular drug pressures were performed to maintain its phenotype. ART-resistant and ART-sensitive strains, as well as the chloroquine-resistant FcB1-Columbia strain were cultured according to the Trager and Jensen protocol with modifications [43]. Briefly, parasites were cultured in RPMI 1640 (Fisher Scientific, Waltham, Illkirch-Graffenstaden, France) supplemented with 5% type AB human serum (Etablissement français du Sang, Toulouse, France) and adjusted at a parasitemia of 1% and 2% hematocrit with type O human at 37 °C and 5% CO_2_. Parasite development was daily monitored by Giemsa staining.

#### 4.2.2. In Vitro Antimalarial Activities

The antiplasmodial activities of alkoxyamines were first evaluated on the chloroquine-resistant FcB1-Columbia strain at the dose of 10 µM, in triplicate and on 2 independent experiments using the SYBR Green I method [44]. Results are reported in Table S1. Drugs inducing around or more than 50% parasite growth inhibition at 10 µM were then also tested at least twice in a standard chemosensitivity assay to determine their exact IC_50_ values (values in bold-face type in Table S1, Supporting Information). The molecules with the best IC_50_ values (a total of 18) were then also evaluated on both F32-ART5 and F32-TEM strains (Table 1). Artemisinin (TCI Europe N.V., Eschborn, Germany) and chloroquine (Sigma-Aldrich, Saint-Quentin-Fallavier, France) were used as antimalarial reference compounds. Briefly, for the determination of IC_50_ values, the chemosensitivity assay was carried out in 96-well culture plates on D-sorbitol ring-stage synchronized parasites [45] and antiplasmodial activities were evaluated using the SYBR Green I method [44]. Drug testing was performed in triplicates in a dose range of 5 to 7 concentrations. All the compounds were received as powders and dissolved in DMSO to a stock solution of 10 mg/mL, except for chloroquine dissolved in RPMI 1640. Serial dilutions were carried out in RPMI 1640 to yield a final DMSO concentration of 1% in the well, a concentration that was verified as not affecting parasite growth. Parasites were left to proliferate with the given compounds for 48 h at 37 °C and 5% CO_2_. After this period, pellets were washed 3 times in sterile PBS prior to putting the plates at −20 °C overnight to lyse all red blood cells. The following day, the plates were thawed at room temperature, 100 µL of each well was transferred into a black 96-well plate to which another 100 µL of 2× PBS-diluted SYBR Green I (Sigma-Aldrich) was added and left to incubate for 2 h at room temperature prior to reading the plates on BioTek FL× 800 Microplate Fluorescence Reader (*λ*_excitation_ = 485 nm, *λ*_emission_ = 528 nm). Control parasite culture (i.e., RPMI with 1% DMSO) was referred to as 100% growth, and the percentage of growth inhibition was determined as follows:

$$\%\;inhibition=100-100\;\times \;\frac{Signal\left(molecule\right)-Signal\left(background\;noise\right)}{Signal\left(DMSO\right)-Signal\left(background\;noise\right)}$$

IC_50_ values were determined using GraphPad Prism by drawing the curve % inhibition vs. [Drug] and using a four-parameter dose–response curve with the following equation:

$$Y=Bottom+\;\frac{Top-Bottom}{1+\frac{x^{Hill\;Slope}}{IC_{50}^{Hill\;Slope}}}$$

#### 4.2.3. Recrudescence Assay

The lead compound, (*RS/SR*)-**2F**, was tested in a recrudescence assay, to assess cross-resistance with artemisinin, according to Ménard et al. [5]. Briefly, F32-TEM and F32-ART5 parasites were synchronized at ring-stage by D-sorbitol treatment prior to treating them with the drug of interest for 48 h, then washed and allowed to grow in drug-free medium. Parasitemia was monitored every other day for 30 days to determine the day when parasite cultures reached the t_0_ initial parasitemia. The drug concentration of 5 µM tested for *(RS/SR)*-**2F** was determined with preliminary tests and 10-fold of the IC_50_ was retained. Artemisinin was used as control to confirm differences in recrudescence times between both strains [42]. Three independent experiments were performed for (*RS/SR*)-**2F** and artemisinin tested in parallel. Results are displayed in Table 2.

### 4.3. Evaluation against S. mansoni

#### 4.3.1. Parasite Culture

The NMRI strain of *Schistosoma mansoni* was used for the experimentations. The parasite was maintained on an albino variety of *Biomphalaria glabrata* from Brazil (intermediate host) and in Golden Hamsters, *Mesocricetus auratus* (Janvier Labs, Le Genest-Saint-Isle, France). Detailed methods, for mollusk and hamster infections and for parasite recovery, were previously described [46].

#### 4.3.2. In Vitro Antischistosomal Activities

Adult worms were recovered between 42 and 47 days after infection by hepatic perfusion technique. Twenty to fifty adult worms were disposed in 12-well Falcon^®^ plate containing 2 mL of RPMI 1640 (supplemented with L-glutamine and Hepes 25 mM) and stored in an incubator chamber (37 °C; 5% CO_2_). Drug preparation started with dissolution of the drug in DMSO to give a 100 mg/mL stock solution. The dilution was complemented with Tween 80 and RPMI in order to obtain the following final ratio dilution: RPMI 1640/Tween 80/DMSO, 1000/0.95/3.8, *v*/*v*/*v*. The drug solution was added to Falcon plates that contained adult worms. The *S. mansoni* cultures were then incubated with each drug at a final concentration of 100 µg/mL. Positive control was carried out with a solution of praziquantel (Merck KGaA, Darmstadt, Germany) at a concentration of 100 µg/mL. Negative control was carried out with the same RPMI 1640/Tween 80/DMSO solution, but without any drug. Each test was performed in duplicate. Every hour, moving worms were counted in order to define the percentage of survivors. Parasites showing no body contractions during a 30 s observation were considered as dead. Observations were extended to 8 h. Mean survival times (± standard deviation) were calculated for each drug using Kaplan–Meier survival analyses.

Housing, feeding and animal care followed the national ethical standards established in the writ of February 1st, 2013 (NOR: AGRG1238753A). The IHPE lab possesses the permit A66040 for the animal experiments and certificate for the experimenters (authorization 007083, decree 87–848).

### 4.4. Cytotoxicity Assays

Cytotoxicity of the compounds was evaluated against Vero cells, a non-cancerous mammalian line, with a similar dilution protocol as for antiplasmodial evaluations (the final DMSO concentration was 0.5% vol). Vero cells were cultured in MEM (Dutscher, Brumath, France) supplemented with 10% fetal bovine serum, 1× non-essential amino acids, 100 U/mL, 100 µg/mL penicillin/streptomycin, and 2 mM L-glutamine at 37 °C in a humidified 5% CO_2_ atmosphere [47]. Vero cells (100 µL of 10^5^ cells/mL per well) were plated in 96-well plates for 24 h, before treating them with 100 µL of drug dilutions. Vero cells were then left to incubate for 48 h with the drugs. At this time, each well was microscope-examined for detection of possible precipitate formation prior to remove the supernatant by flicking the plate. 100 µL of a 0.5 mg/mL PBS-dissolved MTT tetrazolium (stock solution at 5 mg/mL) was then added to each well [48]. The plates were then incubated for 1 h at 37 °C and 5% CO_2_ prior to removing the supernatant and adding 100 µL of DMSO. Plates were gently shaken to dissolve formazan crystals resulting from the MTT tetrazolium reduction by living cells and read at 570 nm with BioTek µQuant Microplate Spectrophotometer. IC_50_ values were determined using GraphPad Prism, in a similar fashion to what was carried out on *P. falciparum* (% inhibition vs. [Drug]) and the percentage of growth inhibition was determined as follows:

$$\%\;inhibition=100-100\times \frac{Signal\left(molecule\right)}{Signal\left(DMSO\right)}$$

Results are displayed in Table 1.

### 4.5. Computational Studies

The geometry of the structures was optimized at the B3LYP/3-21G* model chemistry with the GAUSSIAN 09 program [49]. The bulk solvent effects were described with the integral equation formalism polarizable continuum Model (IEFPCM) with water as solvent [50]. Vibrational frequencies were computed to confirm the convergence to local minima and to calculate the unscaled zero-point-energy (ZPE) and the Gibb’s free energy at 298 K.

### 4.6. Reactivity of Alkoxyamines toward Heme

#### 4.6.1. Materials and Analytical Conditions

Hemin (Fe^III^(PPIX)Cl) and other commercially available reagents (analytical grade) were purchased from usual suppliers, and used without further purification.

^1^H and ^13^C-NMR spectra were recorded at 293 K on Bruker Avance 400 and Bruker Ascend 600 spectrometers. All chemical shifts for ^1^H were relative to TMS using ^1^H residual chemical shifts of the deuterated solvent as a secondary standard. Diffusion ordered spectroscopy (DOSY) NMR was used to measure the translational diffusion coefficient D. The DOSY spectra were acquired at 293 K with the stebpgp1s pulse program from Bruker topspin software. All spectra were recorded with 16 K time domain data points in the t2 dimension and 16 t1 increments. The gradient strength was linearly incremented in 16 steps from 2% up to 95% of the maximum gradient strength. All measurements were performed with a compromise diffusion delay Δ of 160 ms and a gradient pulse length δ of 2.8 ms. After cooling the reaction mixtures to room temperature, 0.5 mL of each mixture was withdrawn. To this aliquot, a solution of KCN in methanol-*d*_4_ was added [320 mM, 10 μL, 3 mole equivalent of CN^–^ with respect to Fe(III)]. Final DMSO/methanol ratio = 98/2, *v*/*v*.

LC-MS analyses were carried out on an LCQ Fleet ThermoFisher equipment, with a Waters X-Bridge C18 column (5 µm or 3.5 µm, 4.6 × 100 mm). The samples were prepared as follows: an aliquot (5 µL) of the reaction mixture was diluted in DMSO (1.0 mL). For analysis of the reaction of **2F** with heme, eluents were (A) water/methanol/formic acid, 40/60/1, *v*/*v*/*v*, and (B) methanol/formic acid, 100/1, *v*/*v*. The gradient was the following: from A/B = 100/0 to A/B = 20/80 in 15 min, then A/B = 20/80 until 30 min. Flow rate was 0.4 mL/min. UV-vis detection was at 254 nm and 406 nm. For analysis of the reaction of **8F** with heme, eluents were (A) water/formic acid, 100/0.1, *v/v* and (B) acetonitrile/formic acid, 100/0.1, *v*/*v*. The gradient was the following: from A/B = 65/35 until 1 min, then from A/B = 65/35 to A/B = 30/70 in 11 min; A/B was then kept at 30/70 for 1 min, then increased to 5/95 in 3 min, and kept at 5/95 for 4 min. Flow rate was 0.5 mL/min. UV-vis detection was at 260 nm, 279 nm and 400 nm.

#### 4.6.2. Reaction of (*RR*/*SS*)-1a or (*RS*/*SR*)-1a with Fe(III)-heme

In a schlenk tube flushed with Ar, Fe^III^(PPIX)Cl (4.9 mg) and **1a** (3.0 mg) were added. Degassed DMSO was then added (0.8 mL, [heme] = [**1a**] = 9.4 mM), and the mixture was heated at 80 °C for 6 h, under magnetic stirring. The reaction was monitored as follows: an aliquot (5 μL) of the reaction mixture was then diluted in DMSO (250 μL) for LC-MS analyses. t_R_ [heme) = 9.6 min, *m/z* = 616.6 (M^+^); t_R_ [**1a**] = 10.4 min, *m/z* = 401.2 (MH^+^); t_R_ [(R_1_,R_2_–NO) = 10.5 min, *m/z* = 295.2 (M^+^), 317.2 (M + Na^+^).

#### 4.6.3. Reaction of (*RS*/*SR*)-2F and (*RR*/*SS*)-2F with Fe(III)-heme

For reactions carried out under air, Fe^III^(PPIX)Cl (3.7 mg) and **2F** (3.3 mg) were heated at 95 °C for 6 h, in DMSO-*d*_6_ (0.8 mL) under magnetic stirring ([heme] = [**2F**] = 6.7 mM). When the reaction was carried out under argon, the schlenk tube was flushed with Ar prior to the addition of Fe^III^(PPIX)Cl and **2F**. Degassed DMSO-*d*_6_ (0.8 mL) was then added, and the mixture was heated at 95 °C for 6 h, under magnetic stirring. After cooling the reaction mixture to room temperature, LC-MS and NMR analyses were carried out as described above. ^1^H-NMR for compound **22** (600 MHz): *δ*, ppm: 8.72 (2H, H6 and H6′′), 8.62 (2H, H3 and H3′′), 8.33 (2H, H3′ and H5′), 8.00 (2H, H4 and H4′′), 7.50 (2H, H5 and H5′′), 2.85 (2H, C4′-C*H*_2_), 1.30 (3H, C4′-CH_2_-C*H*_3_). ^13^C-NMR for compound **22** (151 MHz, partial data): *δ*, ppm: 120.0 (C3′ and C5′), 28.3 (C4′-*C*H_2_), 15.1 (C4′-CH_2_-*C*H_3_). ^1^H-NMR for compound **24** (600 MHz): *δ*, ppm: 8.87 (2H, H3′ and H5′), 8.76 (2H, H6 and H6′′), 8.66 (2H, H3 and H3′′), 8.01 (2H, H4 and H4′′), 7.50 (2H, H5 and H5′′), 2.72 (3H, C4′-CO-C*H*_3_). ^13^C-NMR for compound **24** (151 MHz): *δ*, ppm: 198.0 (*C*O), 156.2 and 154.6 (C2, C2′, C6′ and C2′′), 140.7 (C6 and C6′′), 137.7 (C4 and C4′′), 124.9 (C5 and C5′′), 121.2 (C3 and C3′′), 118.3 (C3′ and C5′), 26.8 (C4′-CO-*C*H_3_). ^1^H-NMR for compound **25** (600 MHz): *δ*, ppm: 8.53 (2H, H3′ and H5′), 8.72 (2H, H6 and H6′′), 8.64 (2H, H3 and H3′′), 7.97 (2H, H4 and H4′′), 7.44 (2H, H5 and H5′′), 4.97 (1H, C4′-C*H*(OH)-CH_3_), 1.45 (3H, C4′-CH(OH)-C*H*_3_). ^13^C-NMR for compound **25** (151 MHz): *δ*, ppm: 155.6 and 154.9 (C2, C2′, C6′ and C2′′), 149.2 (C6 and C6′′), 137.4 (C4 and C4′′), 124.3 (C5 and C5′′), 121.0 (C3 and C3′′), 117.7 (C3′ and C5′), 68.0 (C4′-*C*H(OH)-CH_3_), 25.4 (C4′-CH(OH)-*C*H_3_).

LC-MS: t_R_ [(*RR*/*SS*)-**2F** or (*RS/SR*)-**2F**] = 13.4 min, *m/z* = 571.3 (M + H^+^); t_R_ [22] = 4.7 min, *m/z* = 262.3 (M + H^+^), 284.3 (M + Na^+^); t_R_ [24] = 8.0 and 10.0 min, *m/z* = 521.4 (M + H^+^), 543.4 (M + Na^+^); t_R_ [25] = 5.8 min, *m/z* = 276.3 (M + H^+^), 298.3 (M + Na^+^).

#### 4.6.4. Reaction of (*R*/*S*)-8F with Fe(III)-heme

Fe^III^(PPIX)Cl (40 µL of a solution containing 4.1 mg of Fe^III^(PPIX)Cl in 250 µL of DMSO-*d*_6_, 1.0 µmol) and **8F** (10 µL of a solution containing 16.7 mg of **8F** in 400 µL of DMSO-*d*_6_, 0.97 µmol) were added in a round-bottom flask containing 450 µL of DMSO-*d*_6_. The solution was then heated at 100 °C for 3 h under air, with magnetic stirring ([heme] = [**8F**] = 1.9–2.0 mM). After cooling the reaction mixture to room temperature, LC-MS and NMR analyses were carried out as described above. ^1^H-NMR for compound **24** and **24-*d*** (82% with respect to the terpyridine fragment) (600 MHz): *δ*, ppm: 8.83 (2H, H3′ and H5′), 8.77 (2H, H6 and H6′′), 8.67 (2H, H3 and H3′′), 8.06 (2H, H4 and H4′′), 7.56 (2H, H5 and H5′′), 2.78 (s, 1.35H = 3H × 45 mol% of 24, C4′-CO-C*H*_3_), 2.76 (tr, ^2^J_HD_ = 1.8 Hz, 1.1H = 2H × 55 mol% of 24*-d*, C4′-CO-C*H*_2_D). ^13^C-NMR for compound **24** and **24**-***d*** (151 MHz): *δ*, ppm: 198.1 (C4′-*C*O-CH_3_), 156.7 (C2′ and C6′), 155.0 (C2 and C2′′), 150.0 (C6 and C6′′), 138.0 (C4 and C4′′), 125.5 (C5 and C5′′), 121.5 (C3 and C3′′), 118.2 (C3′ and C5′), 27.7 (*C*H_3_-CO-C4′). D = (2.6 ± 0.1) × 10^−10^ m^2^/s. ^1^H-NMR for compound **25** (10% with respect to the terpyridine fragment) (600 MHz): *δ*, ppm: 8.73 (2H, H6 and H6′′), 8.62 (2H, H3 and H3′′), 8.45 (2H, H3′ and H5′), 8.00 (2H, H4 and H4′′), 7.49 (2H, H5 and H5′′), 4.94 [1H, C4′-C*H*(*-*OH)-CH_3_], 1.42 [3H, C*H*_3_-CH(-OH)-C4′]. ^13^C-NMR for compound **25** (151 MHz): *δ*, ppm: 149.7 (C6 and C6′′), 138.1 (C4 and C4′′), 124.8 (C5 and C5′′), 121.4 (C3 and C3′′), 118.2 (C3′ and C5′), 67.8 [C4′-*C*H(-OH)-CH_3_]. D = (2.1 ± 0.1) × 10^−10^ m^2^/s. ^1^H-NMR for unreacted **8F** (8% with respect to the terpyridine fragment) (600 MHz): *δ*, ppm: 8.73 (2H, H6 and H6′′), 8.62 (2H, H3 and H3′′), 8.42 (2H, H3′ and H5′), 8.00 (2H, H4 and H4′′), 7.49 (2H, H5 and H5′′), 4.99 [1H, C4′-C*H*(*-*OH)-CH_3_], 1.52 [3H, C*H*_3_-CH(-OH)-C4′). ^13^C-NMR for unreacted **8F** (151 MHz): *δ*, ppm: 149.7 (C6 and C6′′), 138.1 (C4 and C4′′), 124.8 (C5 and C5′′), 121.4 (C3 and C3′′), 119.1 (C3′ and C5′). D = (2.1 ± 0.1) × 10^−10^ m^2^/s. For comparison, D [low spin Fe(III)–heme] = (1.4 ± 0.1) × 10^−10^ m^2^/s.

LC-MS: t_R_ [**8F**] = 8.0 ± 0.1 min, *m/z* = 432.9 (M + H^+^); t_R_ [23] = 6.7 ± 0.1 min, *m/z* = 276.1 (M + H^+^); t_R_ [heme] = 10.5 min, *m/z* = 616.3 (M^+^), 693.7 (M^+^ + DMSO).

## Figures and Tables

**Table 1:** Antiplasmodial and antischistosomal activities detailed for the best alkoxyamines and cytotoxicity on Vero cell lines.

	*S. mansoni* Mean Survival Time (h)	*P. falciparum* Strains Mean IC_50_ (µM)		Cytotoxicity Mean IC_50_ (µM)	Selectivity Index ^4^
	Adult *S. mansoni*	F32-TEM	F32-ART5	Vero cell lines	
(*RR*/*SS*)-**1a**	>8	15 ^2^	16 ^2^	17 ± 7.0	1
(*RS*/*SR*)-**1a**	1.2 ± 0.1	7.6 ^2^	11 ^2^	28 ± 29	3
(*RS*/*SR*)-**2F**	2.1 ± 0.1	0.6 ± 0.1	0.5 ± 0.1	0.8 ± 0.3	1
(*RR*/*SS*)-**2F**	2.5 ± 0.2	0.5 ± 0.1	0.4 ± 0.1	0.7 ± 0.3	1
(*RS*/*SR*)-**4F** (*RR*/*SS*)-**4F**	5.2 ± 0.2 >8	2.9 ^2^ 4.6 ^1^	4.7 ^2^ 4.6 ^1^	15 ± 5.0 8.1 ± 3.3	4 2
(*R*/*S*)-**8F**	5.0 ± 0.0	1.3 ^2^	1.1 ^2^	0.7 ± 0.4	0.5
(*RS*/*SR*)-**7F**	2.7 ± 0.1	1.3 ^2^	1.4 ^2^	0.9 ± 0.4	0.6
(*RR*/*SS*)-**7F**	2.5 ± 0.1	0.94 ^2^	0.85 ^2^	1.2 ± 1.3	1
**1J**	1.1 ± 0.1	2.9 ^1^	2.3 ^1^	6.2 ± 1.2	2
**1K**	1.1 ± 0.0	1.1 ^1^	1.6 ^1^	1.1 ± 0.7	1
**2J**	1.0 ± 0.0	5.0 ^1^	4.6 ^1^	17 ± 6.3	3
(*RS*/*SR*)-**1L**	ND ^3^	11 ^2^	18 ^2^	ND	ND
(*RR*/*SS*)-**1L**	ND	20 ^2^	26 ^2^	28	1
(*RS*/*SR*)-**1M**	ND	5.8 ^2^	4.9 ^2^	13 ± 1.5	2
(*RR*/*SS*)-**1M**	ND	4.8 ^2^	6 ^2^	6.5 ± 0.3	1
**22**	>8	0.24 ^1^	0.23 ^1^	5.3 ± 4.5	22
**9a**	3.0 ± 0.0	7.5 ^1^	13 ^1^	40 ± 13	3
(*RS*/*SR*)-**1A**	1.0 ± 0.05	ND	ND	21	
(*RR*/*SS*)-**1A**	1.0 ± 0.05	ND	ND	>100	
(*RR*/*SS*)-**1p**	1.0 ± 0.05	ND	ND	>100	
(*RR*/*SS*)-**1k**	1.0 ± 0.05	ND	ND		
Praziquantel	1.0 ± 0.05	ND	ND	>>100 ^5^	>>100
Artemisinin	ND	0.02 ± 0.001 ^6^	0.02 ± 0.002 ^6^	160 ± 12	8000
Chloroquine	ND	0.06 ± 0.02	0.06 ± 0.03	190 ± 56	3000

^1^*n* = 1; ^2^ *n* = 2; ^3^ ND = not determined; ^4^ selectivity indexes are calculated as the ratio-cytotoxicity/activity on *Plasmodium*. As the activity on *Schistosoma* was based on a single dose treatment at 100 µg/mL, results are reported by a mean survival time, hence the selectivity index on *Schistosoma* cannot be calculated; ^5^ data from Reference [34]; ^6^ F32-ART5 is artemisinin-resistant and F32-TEM is artemisinin-sensitive. However, as artemisinin resistance is a quiescence-based phenomenon [35], these strains cannot be segregated by standard assays based on parasite proliferation. F32-ART and F32-TEM are chloroquine-sensitive.

**Table 2:** Recrudescence assay on artemisinin-resistant (F32-ART5) and artemisinin-sensitive (F32-TEM) strains.

Drug (Dose)	Median (Range) Recrudescence Time (Days)		Mean ± SEM Difference of Recrudescence Time (Days)	*p*-value
	F32-ART5	F32-TEM		
Artemisinin (18 µM) (*RS/SR*)-**2F** (5 µM)	9 (7–11) 16 (15–17)	>30 (16→30) ^1^ >30 ^1^	>16.3 ± 3.7 >14 ± 0.6	0.048 0.0017

^1^ If no parasites were observed at the end of the experiment (i.e., after 30 days), the culture was classified as showing no recrudescence and the recrudescence day was noted as >30 days. For statistical purposes, the values corresponding to the cultures showing no recrudescence were censored and their values aligned to the last day of monitoring.

**Table 3:** Experimental homolysis rate constant *k*_d_’ for alkoxyamines **16**, **2F**, **8F**, **9F**, **2G** and **6G** using the EPR technique and Equation (2), in various conditions, corresponding activation energies *E*_a_, re-estimated homolysis rate constant *k*_d_ at 120 °C, and half-lifetime t_1/2_ at 37 °C.

	Solvent	*T* (°C) *^a^*	*k*_d_′ (10^−4^s^−1^) *^b^*		*E*_a_ (kJ/mol) *^c,d^*		*k*_d_ (10^−3^ s^−1^) *^d^*		*t*_1/2_ (day) *^e^*	
			*RS*/*SR ^f^*	*RR*/*SS ^f^*	*RS*/*SR ^f^*	*RR*/*SS ^f^*	*RS*/*SR ^f^*	*RR*/*SS ^f^*	*RS*/*SR ^f^*	*RR*/*SS ^f^*
**6F**	*t*-BuPh	81	2.7	1.4	121.7	123.8	16.3	8.5	10	24
**2F**	*t*-BuPh	81	5.3	7.0	119.7	118.9	29.9	38.4	4.8	3.5
**2FH^+^**	*t*-BuPh + 2eq TFA *^g^*	80	8.7	6.7	117.9	118.2	51.8	40.9	2.4	3.3
**2F**	MeOH/water 1:1 *p*H = 7.4 *^h^*	81	8.7	7.1	118.2	118.8	46.7	38.9	2.7	3.5
**2FH^+^**	MeOH/water 1:1 *p*H = 1.0 *^h^*	51	2.1	12.0	111.9	107.4	310.1	1305	5.6 h	1.0 h
**2FFe^2+^**	MeOH/water 1:1 *p*H = 7.4 *^h^* FeCl_2_	60	17.8	-*^i^*	109.2	-*^j^*	732.0	-*^j^*	2 h	-*^j^*
**2FFe^2+^**	MeOH/water 1:1 *p*H = 7.4 *^h^* FeCl_2_	51	-*^i^*	6.5	-*^j^*	109.0	-*^j^*	787.0	-*^j^*	1.8 h
**2FFe^3+^**	MeOH/water 1:1 *p*H = 7.4 *^h^* FeCl_3_	51	3.9	-*^i^*	116.4	-*^j^*	83.4	-*^j^*	1.3	-*^j^*
**2FFe^3+^**	MeOH/water 1:1 *p*H = 7.4 *^h^* FeCl_3_	61	-*^i^*	2.0	-*^j^*	115.1	-*^j^*	120.6	-*^j^*	8.1
**9F**	*t*-BuPh	111	3.7 *^k^*		131.0 *^k^*		0.95 *^k^*		385 *^k^*	
**9FH^+^**	*t*-BuPh + 2 eq. TFA *^g^*	101	3.0 *^k^*		128.2 *^k^*		2.2 *^k^*		385 *^k^*	
**9F**	MeOH/water 1:1 *p*H = 7.4 *^h^*	90	0.25 *^k^*		131.9 *^k^*		0.7 *^k^*		385 *^k^*	
**8F**	*t*-BuPh	111	1.9		133.3 *^k^*		0.46		941*^k^*	
**6G**	*t*-BuPh	92	3.5	2.7	124.7	125.5	6.50	5.10	45.3	33.4
**2G**	*t*-BuPh	81	3.7	3.8	120.8	120.8	21.6	21.1	7.5	7.5
**2G**	MeOH/water 2:1 *p*H = 7.0 *^h^*	81	2.3	3.3	122.1	121.1	14.4	19.5	12.7	12.3

*^a^* Error ± 1 °C. *^b^* Statistical error less than 5%. *^c^* Currently accepted error ± 1 kJ/mol. *^d^* Estimated using Equation (1) and assuming an averaged frequency factor A = 2.4 × 10^14^ s^−1^. *^e^* Unless otherwise mentioned. *^f^* Diastereoisomers were separated unless otherwise mentioned. *^g^* TFA: trifluoroacetic acid. Protonation was checked by ^1^H-NMR. *^h^ p*H was corrected as reported in [37]. *^i^* Not measured. *^j^* Not estimated. *^k^* No diastereoisomers. *^l^* Mixture of diastereoisomers afforded only monoexponential growth of nitroxide meaning that the two diastereoisomers exhibit the same values of *k*_d_.

## Supplementary Materials

The following are available online. All structures of alkoxyamines whose biological evaluation is reported in Table S1: Antiplasmodial activities of alkoxyamines against chloroquine resistant FcB1-Columbia strain, and antischistosomal activities on adult *S. mansoni* are reported. Preparation and characterization of **18**, (*RS/SR*)-**2F**, (*RS/SR*)-**4F**, (*RS/SR*)-**6F**, (*RR*/*SS*)-**2F**, (*RR*/*SS*)-**4F**, (*RR*/*SS*)-**6F**, (*R*/*S*)*-***8F,** (*RS*/*SR*)-**2G**, (*RR*/*SS*)-**2G**, (*RS*/*SR*)-**6G**, (*RR*/*SS*)-**6G**, and XRD data of (*RR*/*SS)*-**6F** and (*RR*/*SS)*-**6G** as well as ^1^H-NMR spectrum (*RS/SR*)-**2FH^+^** and (*RR*/*SS*)-**2FH^+^** in *t*-BuPh, are reported, see supplementary materials.
