# Supplementary material for: Alkoxyamines Designed as Potential Drugs against Plasmodium and Schistosoma Parasites
Source: Molecules. 2020 Aug 24;25(17):3838. doi: 10.3390/molecules25173838 (PMC7503767; doi:10.3390/molecules25173838)

Alkoxyamines designed as potential drugs against *Plasmodium* and *Schistosoma* parasites

T. Reyser *et al*.

Supporting Information

Figure S1. Structures of alkoxyamines whose biological evaluation is reported in Table S1. **(A)** Structures of nitroxide radicals R_1,R_2–NO•; **(B)** Structures of alkyl radicals R_3,R_4,R_5–C•.

Table S1. Antiplasmodial activities of alkoxyamines against chloroquine resistant FcB1-Columbia strain, and antischistosomal activities on adult *S. mansoni*. The numbering of alkoxyamines is made of the number **1-14**, corresponding to the nitroxide moiety, and the letter **a-z** or **A-O**, corresponding to the alkyl moiety (for the structures, see Figure S1, panels A and B, respectively). The IC_{50} values of artemisinin and chloroquine are provided for comparison. ND = not determined.

Figure S2. NMR spectra of 4'-ethyl-2,2':6',2"-terpyridine

Figure S3. NMR spectra of 4'-(1-bromoethyl)-2,2':6',2"-terpyridine **18**.

Figure S4. NMR spectra of (*RS/SR*)-**6F**.

Figure S5. NMR spectra of (*RR/SS*)-**6F**.

Figure S6. NMR spectra of (*RS/SR*)-**2F**.

Figure S7. NMR spectra of (*RR/SS)*-**2F**.

Figure S8. NMR spectrum of (*RS/SR*)-aldehyde derivative.

Figure S9. NMR spectrum of (*RR/SS*)-aldehyde derivative.

Figure S10. NMR spectrum of (*RS/SR*)-**4F**.

Figure S11. NMR spectrum of (*RR/SS*)-**4F**.

Figure S12. NMR spectrum of (*R/S*)-**8F**

Figure S13. NMR spectrum of (*RS*/*SR*)-**6G**.

Figure S14. NMR spectrum of (*RR*/*SS*)-**6G**.

Figure S15. NMR spectrum of (*RS*/*SR*)-**2G**.

Figure S16. NMR spectrum of (*RR*/*SS*)-**2G**.

Table S2. XRD data for (*RR*/*SS)*-**6F**.

Table S3. XRD data for (*RR*/*SS)*-**6G**.

Figure S17. Proposed mechanism for the formation of products **23** and **24**.

Figure S18. ^{1}H NMR spectrum of (*RS/SR*)-**2F** and (*RS/SR*)-**2FH$^+$** in *t*-BuPh.

Figure S19. ^{1}H NMR spectrum of (*RR/SS*)-**2F** and (*RR/SS*)-**2FH$^+$** in *t*-BuPh.

Figure S1. Structures of alkoxyamines whose biological evaluation is reported in Table S1.

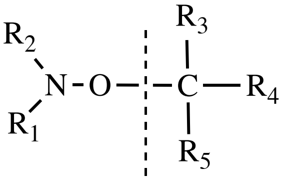

(**A**) Nitroxyl radical **1-13** | (**B**) Alkyl radical **a-z** and **A-I**

(A) Structures of nitroxide radicals R_1,R_2–NO•.

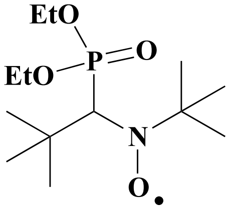

1

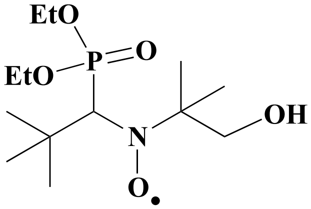

2

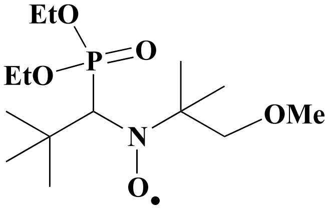

3

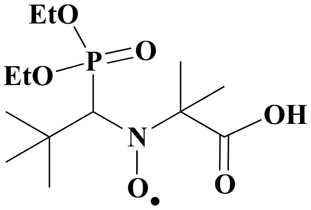

4

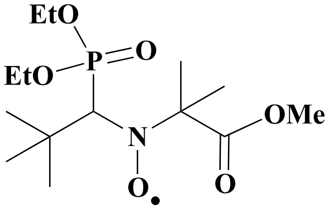

5

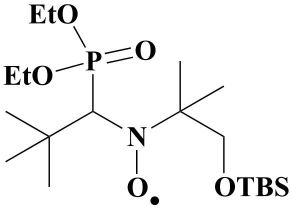

6

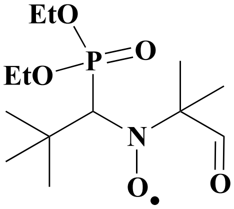

7

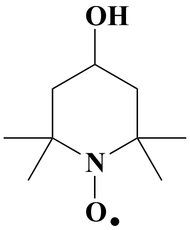

8

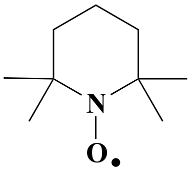

9

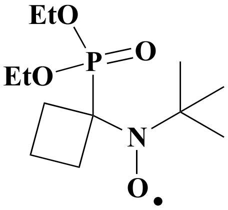

10

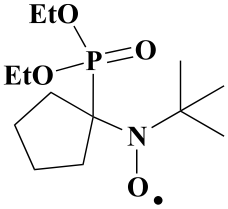

11

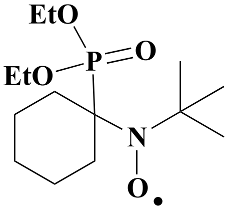

12

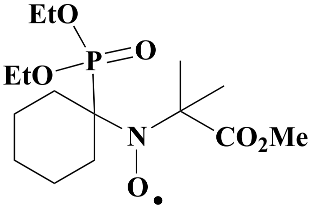

13

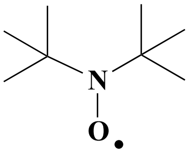

14

(B) Structures of alkyl radicals R_3,R_4,R_5–C.

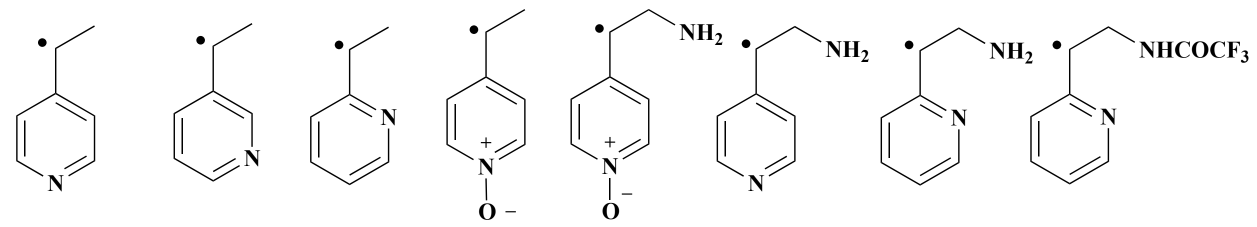

a **b** **c** **d** **e** **f** **g** **h**

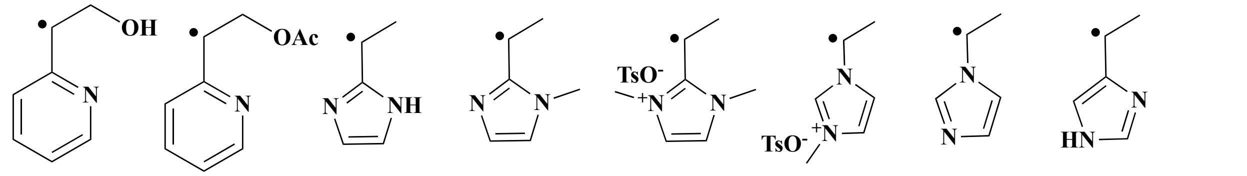

i **j** **k** **l** **m** **n** **o** **p**

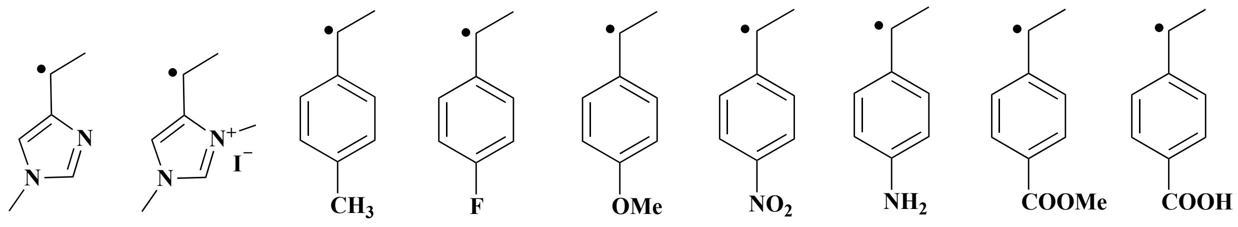

q **r** **s** **t** **u** **v** **w** **x** **y**

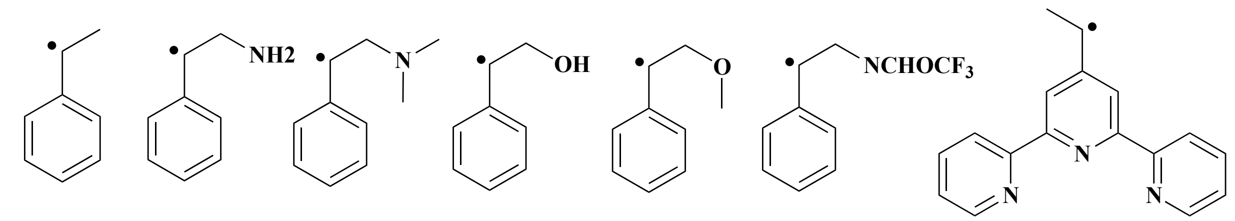

z **A** **B** **C** **D** **E** **F**

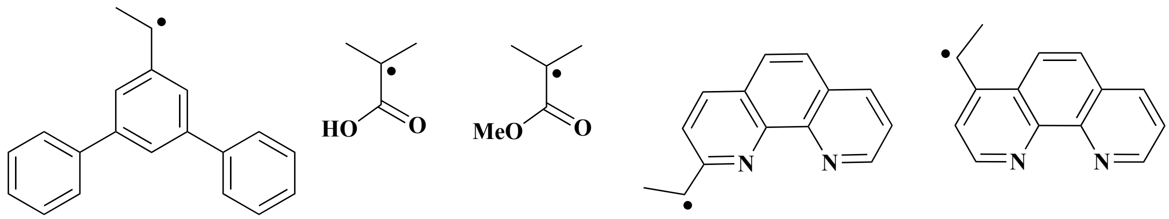

G **H** **I** **J** **K**

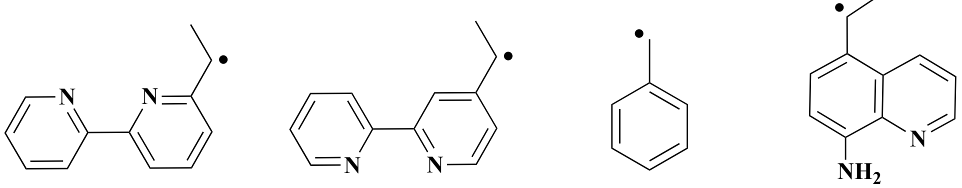

L **M** **N** **O**

Table S1. Antiplasmodial activities of alkoxyamines against the *P. falciparum* chloroquine-resistant strain, FcB1-Columbia, and antischistosomal activities on adults and worms of *S. mansoni*. The numbering of alkoxyamines is made of the number **1-14**, corresponding to the nitroxide moiety, and the letter **a-z** or **A-O**, corresponding to the alkyl moiety (for the structures, see Figure S1, panels A and B, respectively). The IC_{50} values of artemisinin and chloroquine are provided for antiplasmodial comparison; The value of praziquantel is provided for antischistosomal comparison. ND = not determined.

Compounds	IC_{50} (µM) on *P. falciparum*	Mean (±SD) survival time – Adult *S. mansoni* *(hours)*	Time for the death of 100% worms (hours)	Compounds	IC_{50} (µM) on *P. falciparum*	Mean (±SD) survival time – Adult *S. mansoni* *(hours)*	Time for the death of 100% worms
**1**	>> 10	> 8	>8	**(*RR/SS*)-1v**	>> 10	3.4 ± 0.2	4
**(*RS/SR*)-1a**	**3.8**	**1.2 ± 0.1**	2	**(*RS/SR*)-1v**	>> 10	> 8	>8
**(*RR/SS*)-1a**	>> 10	> 8	>8	**(*RR/SS*)-1w**	>> 10	2.1 ± 0.1	3
**(*RS/SR*)-1b**	>> 10	ND	ND	**(*RS/SR*)-1w**	>> 10	> 8	>8
**(*RR/SS*)-1b**	**22.5**	ND	ND	**(*RR/SS*)-1x**	>> 10	> 8	>8
**(*RR/SS*)-1c**	>> 10	ND	ND	**(*RS/SR*)-1x**	>> 10	2.4 ± 0.3	4
**(*RR/SS*)-1d**	>> 10	> 8	>8	**(*RR/SS*)-1y**	>> 10	> 8	>8
**(*RS/SR*)-1e**	>> 10	> 8	>8	**(*RS/SR*)-1y**	>> 10	> 8	>8
**(*RS/SR*)-1f**	>> 10	ND	ND	**(*RS/SR*)-1A**	>> 10	**1.0 ± 0.05**	1
**(*RS/SR*)-1g**	>> 10	2.6 ± 0.3	4	**(*RR/SS*)-1A**	>> 10	**1.0 ± 0.05**	1
**(*RR/SS*)-1g**	>> 10	1.9 ± 0.1	2	**(*RS/SR*)-1B**	>> 10	ND	ND
**(*RS/SR*)-1h**	>> 10	> 8	>8	**(*RS/SR*)-1C**	>> 10	1.4 ± 0.2	2
**(*RS/SR*)-1i**	>> 10	> 8	>8	**(*RR/SS*)-1C**	>> 10	ND	ND
**(*RR/SS*)-1i**	>> 10	> 8	>8	**(*RS/SR*)-1D**	>> 10	3.8 ± 0.5	6
**(*RS/SR*)-1j**	>> 10	> 8	>8	**(*RS/SR*)-1E**	>> 10	> 8	>8
**(*RR/SS*)-1j**	>> 10	> 8	>8	**(*RR/SS*)-1E**	>> 10	5.0 ± 0.5	6
**(*RR/SS*)-1k**	>> 10	**1.0 ± 0.05**	1	**1F**	>> 10	ND	ND
**(*RS/SR*)-1k**	>> 10	> 8	>8	**1H**	>> 10	> 8	>8
**(*RR/SS*)-1l**	>> 10	4.5 ± 0.5	6	**(*RS/SR*)-2c**	>> 10	ND	ND
**(*RS/SR*)-1l**	>> 10	> 8	>8	**(*RR/SS*)-2c**	>> 10	ND	ND
**(*RR/SS*)-1m**	>> 10	> 8	>8	**(*RS/SR*)-2z**	>> 10	> 8	>8

**(*RS/SR*)-1m**	>> 10	5.1 ± 0.4	6	**(*RS/SR*)-2F**	**0.3**	2.1 ± 0.1	**3**
**(*RR/SS*)-1o**	**25**	2.3 ± 0.2	3	**(*RR/SS*)-2F**	**0.2**	2.5 ± 0.2	**5**
**(*RS/SR*)-1o**	**25**	**1.1 ± 0.1**	2	**(*RS/SR*)-3z**	>> 10	ND	ND
**(*RR/SS*)-1n**	>> 10	> 8	>8	**(*RS/SR*)-4z**	>> 10	> 8	>8
**(*RS/SR*)-1n**	>> 10	> 8	>8	**(*RR/SS*)-4z**	>> 10	> 8	>8
**(*RR/SS*)-1p**	**20.5**	**1.0 ± 0.05**	1	**(*RS/SR*)-4F**	**2.9**	5.2 ± 0.2	**7**
**(*RS/SR*)-1p**	**20.5**	**1.4 ± 0.3**	3	*(RR/SS)*-**4F**	**1.3 ± 0.7**	> 8	>8
**(*RR/SS*)-1q**	>> 10	3.3 ± 0.9	6	**(*RS/SR*)-5z**	>> 10	> 8	>8
**(*RS/SR*)-1q**	>> 10	**1.3 ± 0.2**	2	**5I**	>> 10	ND	ND
**(*RR/SS*)-1r**	>> 10	3.0 ± 0.8	4	**6c**	ND	ND	ND
**(*RS/SR*)-1r**	>> 10	3.1 ± 0.1	6	**7z**	ND	ND	ND
**(*RR/SS*)-1s**	>> 10	> 8	>8	**10z**	>> 10	ND	ND
**(*RS/SR*)-1s**	>> 10	3.3 ± 0.1	4	**11z**	>> 10	ND	ND
**(*RR/SS*)-1t**	>> 10	ND	ND	**12z**	>> 10	ND	ND
**1u**	>> 10	ND	ND	**13z**	>> 10	ND	ND
(*RR/SS*)-**2G**	>> 10	6.4 ± 0.1	7	**8 F**	**0.14 ± 0.03**	5.0 ± 0.0	5
(*RS/SR*)-**2G**	>> 10	> 8	>8	**2**	>> 10	> 8	>8
**5N**	>> 10	ND	ND	**8**	>> 10	**>** 8	>8
**5a**	>> 10	ND	ND	**22**	**0.91 ± 0.06**	> 8	>8
**7z**	>> 10	ND	ND	**2b**	>> 10	>8	>8
(*RS/SR*)-**7F**	**2.1 ± 2.2**	2.7 ± 0.1	3	**9b**	>> 10	3.0 ± 0.0	3
(*RR/SS*)-**7F**	**1.5 ± 0.8**	2.5 ± 0.1	3	**8b**	>> 10	> 8	>8
**1J**	**3.1 ± 1.1**	1.1 ± 0.05	2	**9z**	>> 10	> 8	>8
**14a**	>> 10	2.5± 0.10	3	**9a**	**8 ± 3**	3.0 ± 0.0	3
**2a**	>> 10	> 8	>8	**8a**	>> 10	> 8	>8
**1h**	>> 10	ND	ND	**8z**	>> 10	1.9 ± 0.1	2
**1g**	>> 10	ND	ND	(*RS/SR*)-**1a**	>> 10	> 8	>8
**1K**	**1.3 ± 0.3**	1.1 ± 0.04	2	(*RS/SR*)-**1a**	>> 10	> 8	>8
**2J**	**2.5 ± 1.5**	1.0 ± 0.0	1	*(RR/SS)*-**1M**	**4.8**	ND	ND
(*RS/SR*)-**1O**	>> 10	ND	ND	**Artemisinin**	0.04 ± 0.007	ND	ND
(RS/SR)-**1L**	**12**	ND	ND	**Chloroquine**	0.16 ± 0.036	ND	ND
(RR/SS)-**1L**	**28**	ND	ND	**Praziquantel**	ND	1.0 ± 0.05	1
(RS/SR)-**1M**	**2.7**	ND	ND				

Figure S2. NMR of 4'-ethyl-2,2':6',2"-terpyridine.

^{1}H-NMR ($CDCl_3$)

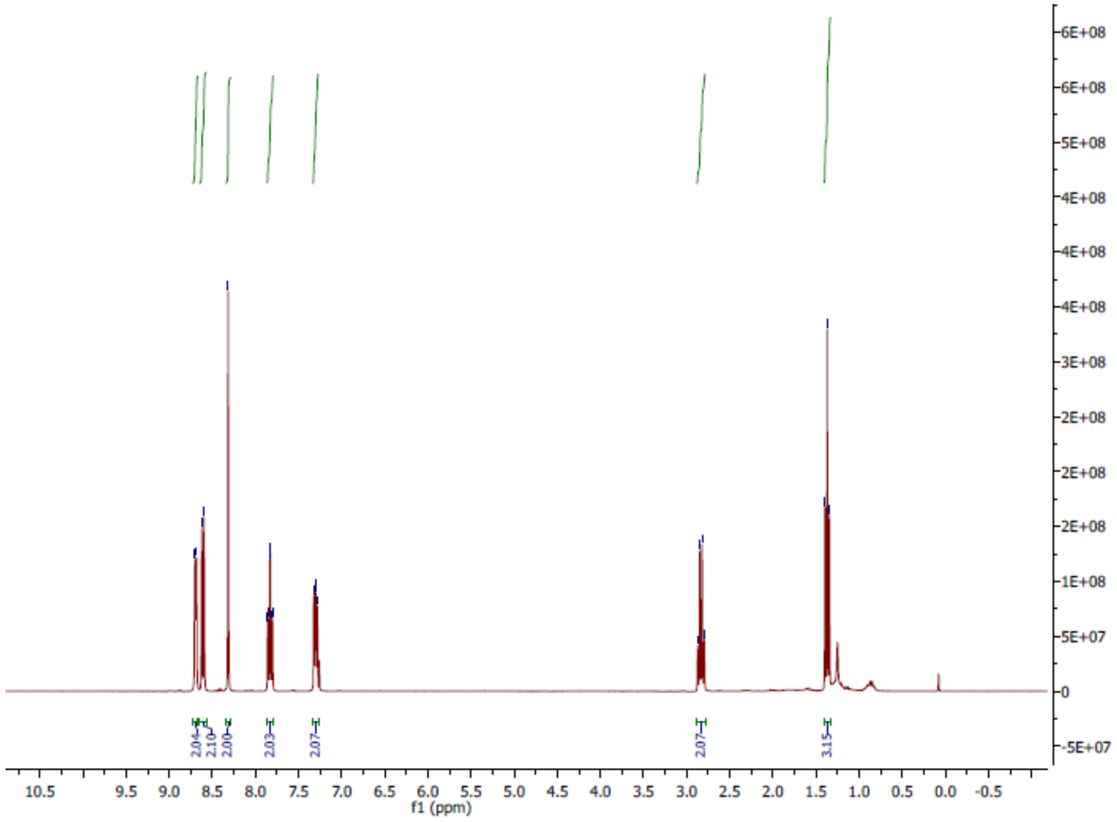

^{13}C-NMR ($CDCl_3$)

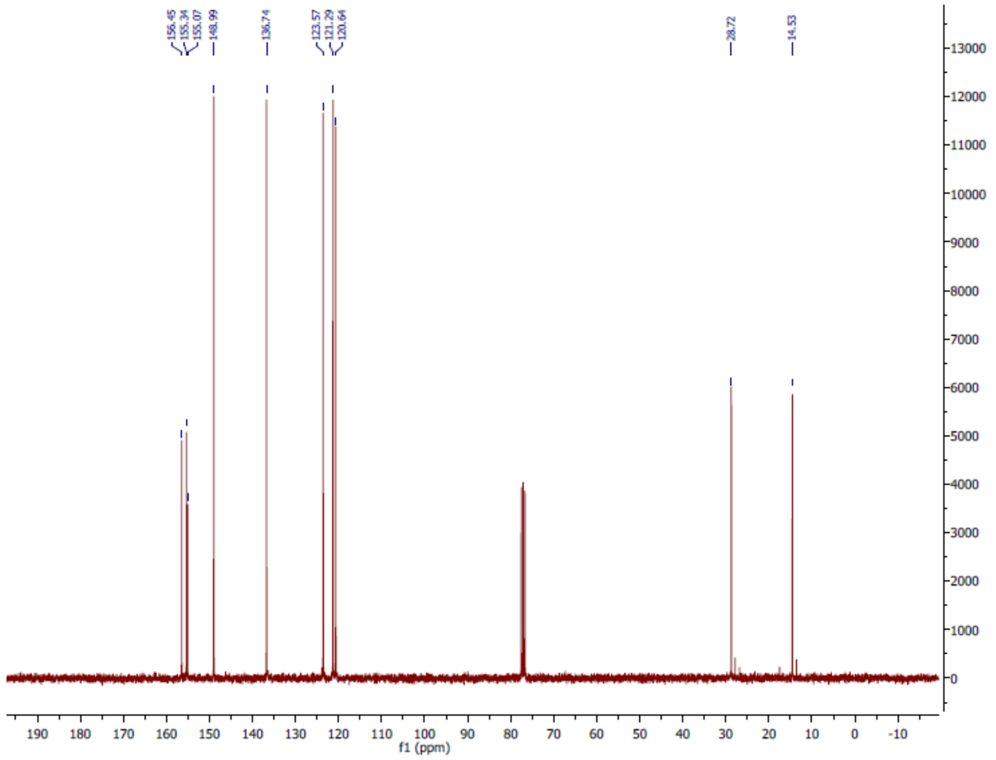

Figure S3. NMR of 4'-(1-bromoethyl)-2,2':6',2''-terpyridine **18**.

^{1}H-NMR ($CDCl_3$)

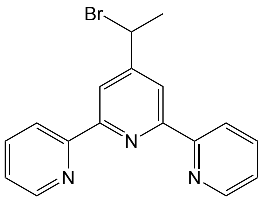

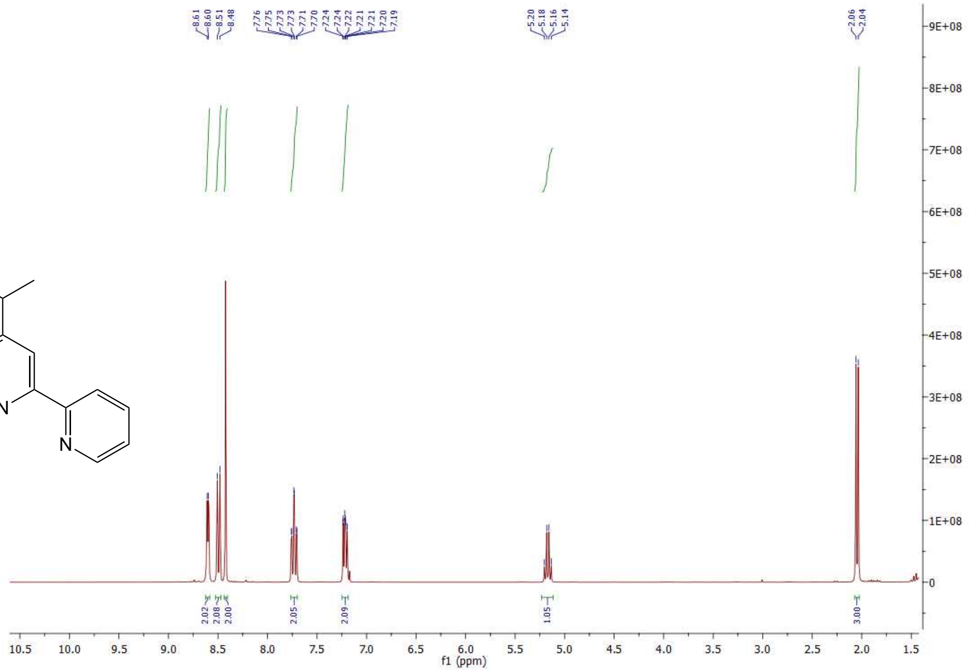

^{13}C-NMR ($CDCl_3$)

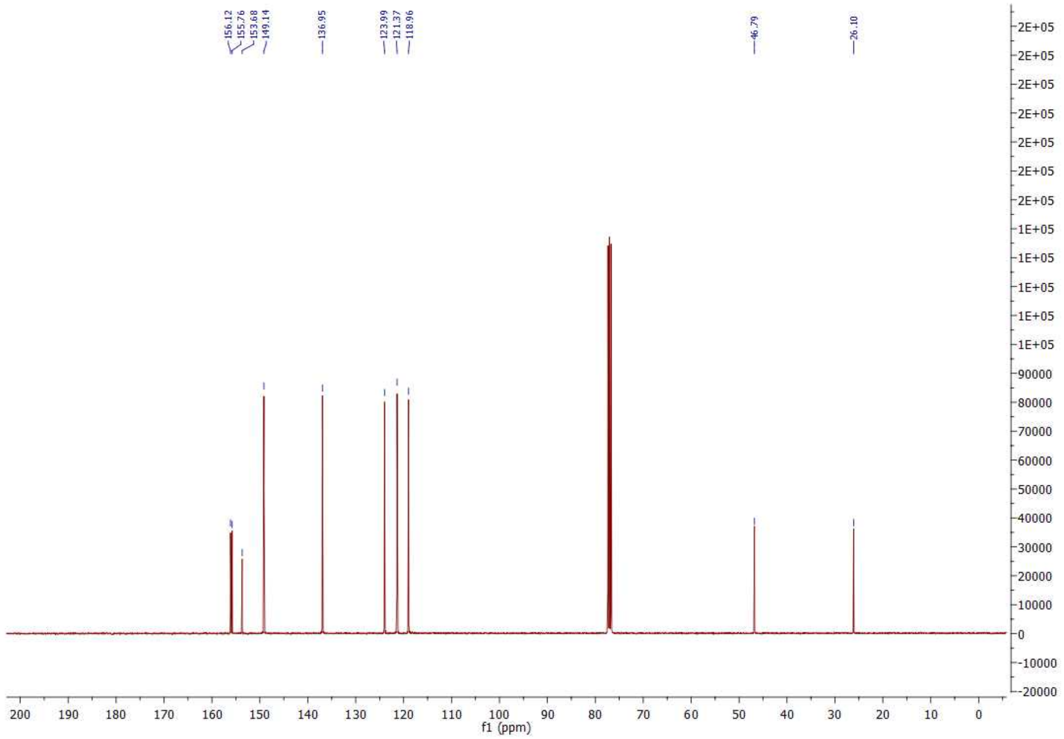

Figure S4. NMR of (*RS/SR)*-**6F**.

^{1}H-NMR ($CDCl_3$)

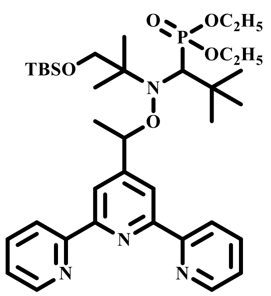

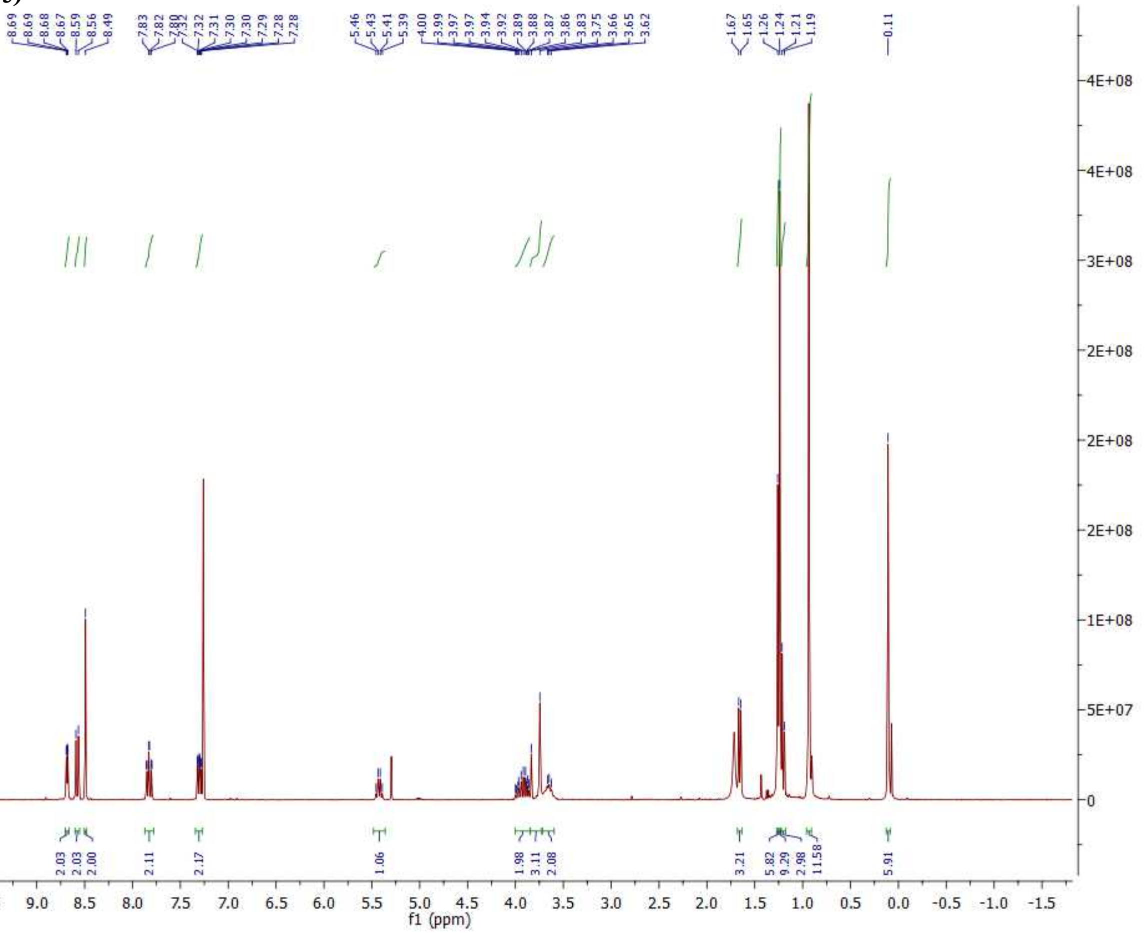

^{13}C-NMR ($CDCl_3$)

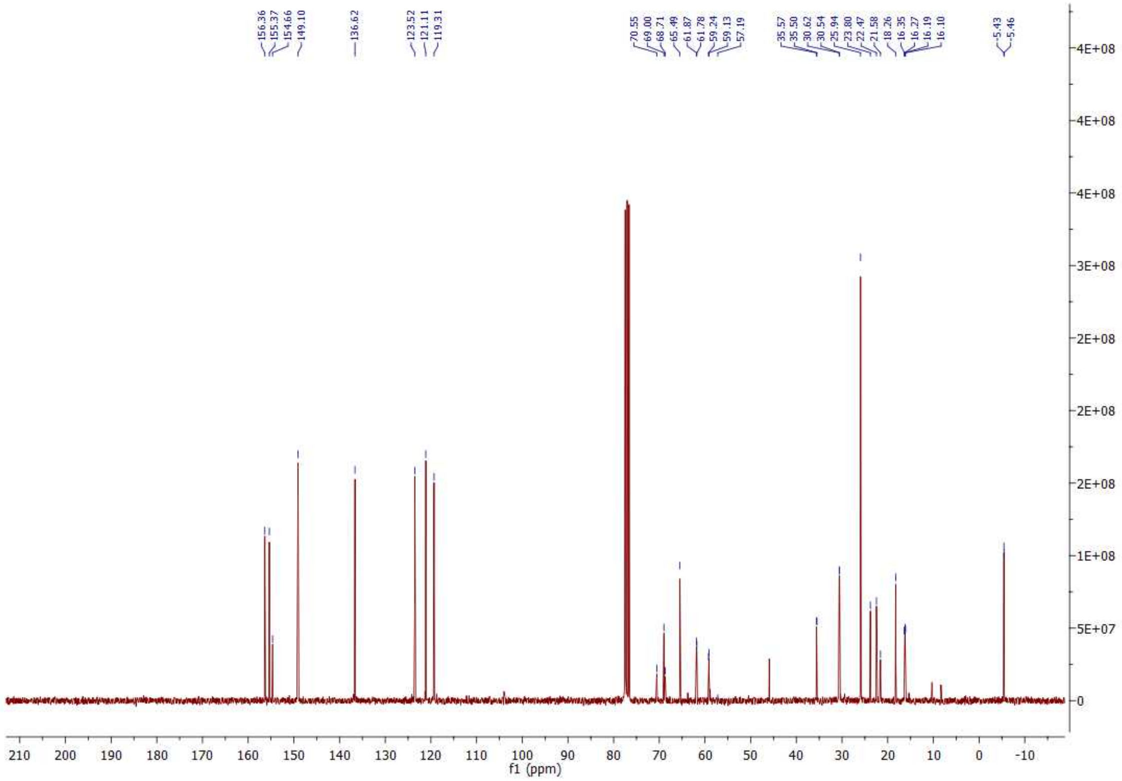

^{31}P NMR ($CDCl_3$)

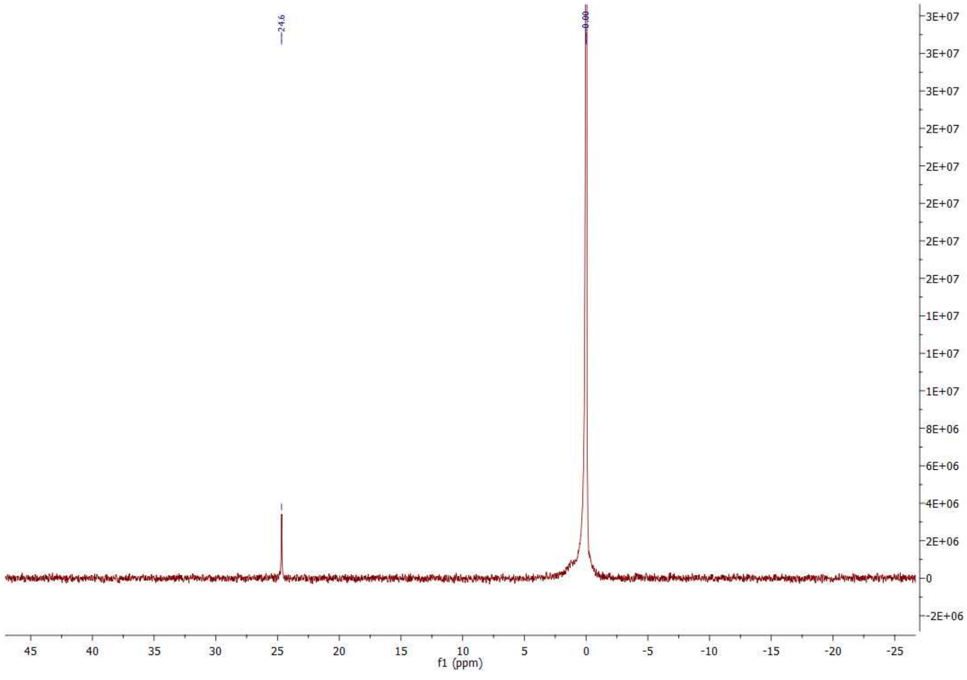

Figure S5. NMR of (*RR/SS)*-**6F**.

^{1}H-NMR (CDCl$_3$)

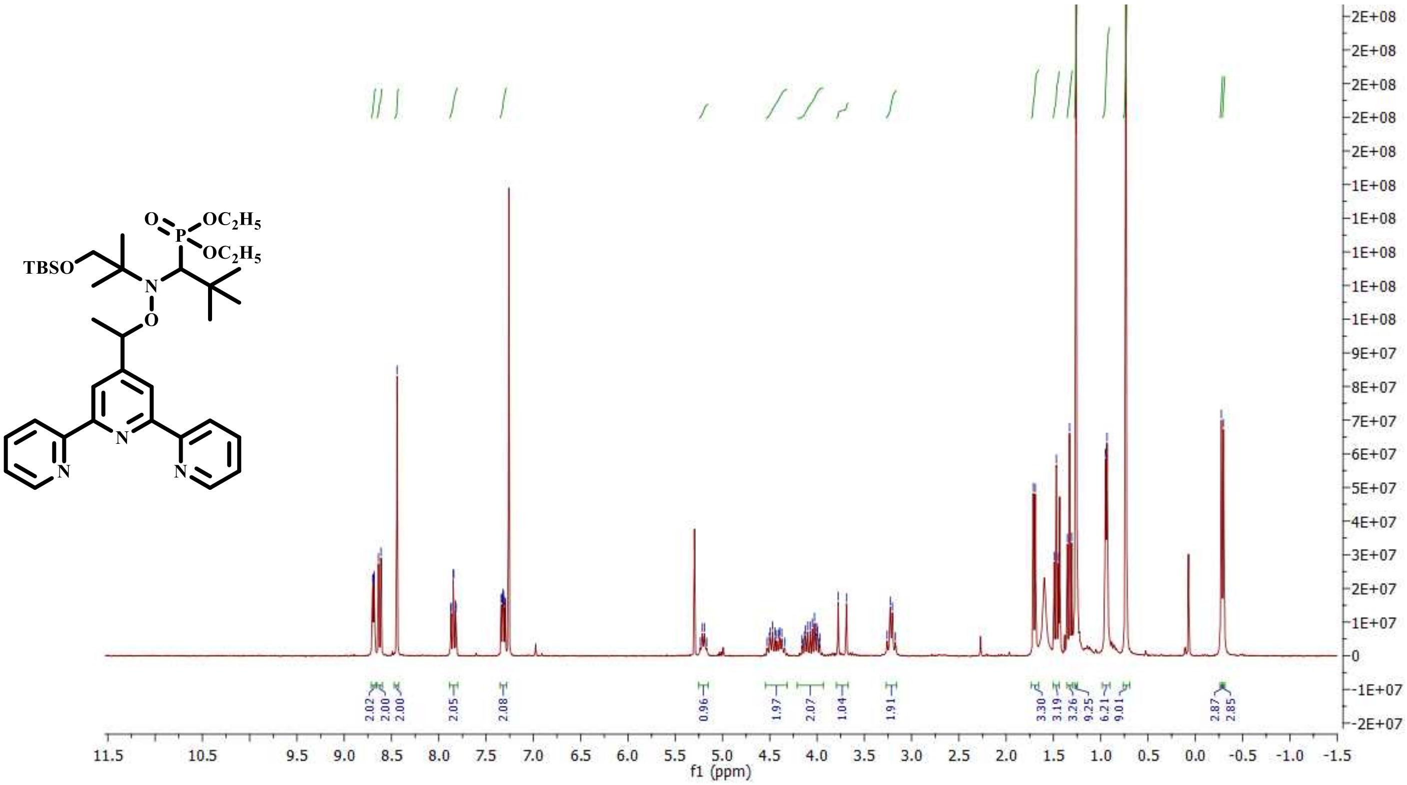

^{13}C-NMR (CDCl$_3$)

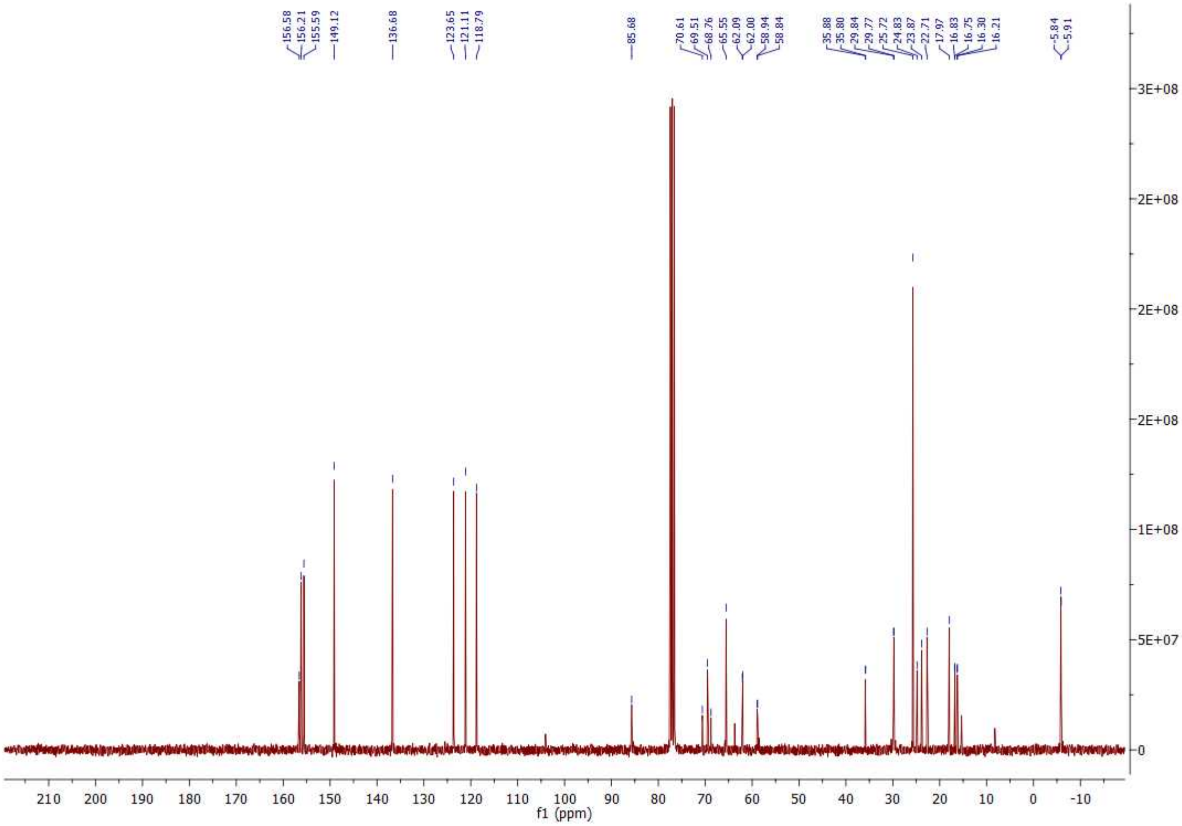

^{31}P-NMR ($CDCl_3$)

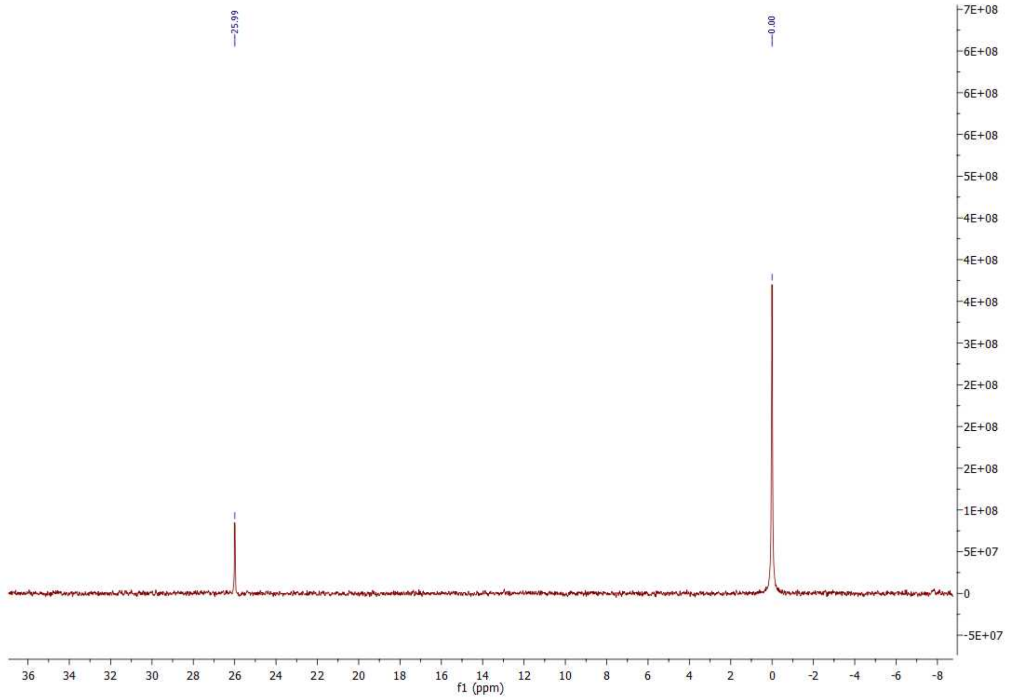

Figure S6. NMR of (*RS/SR*)-**2F**.

^{1}H-NMR ($CDCl_3$)

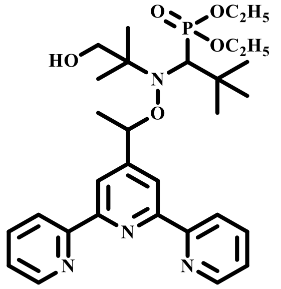

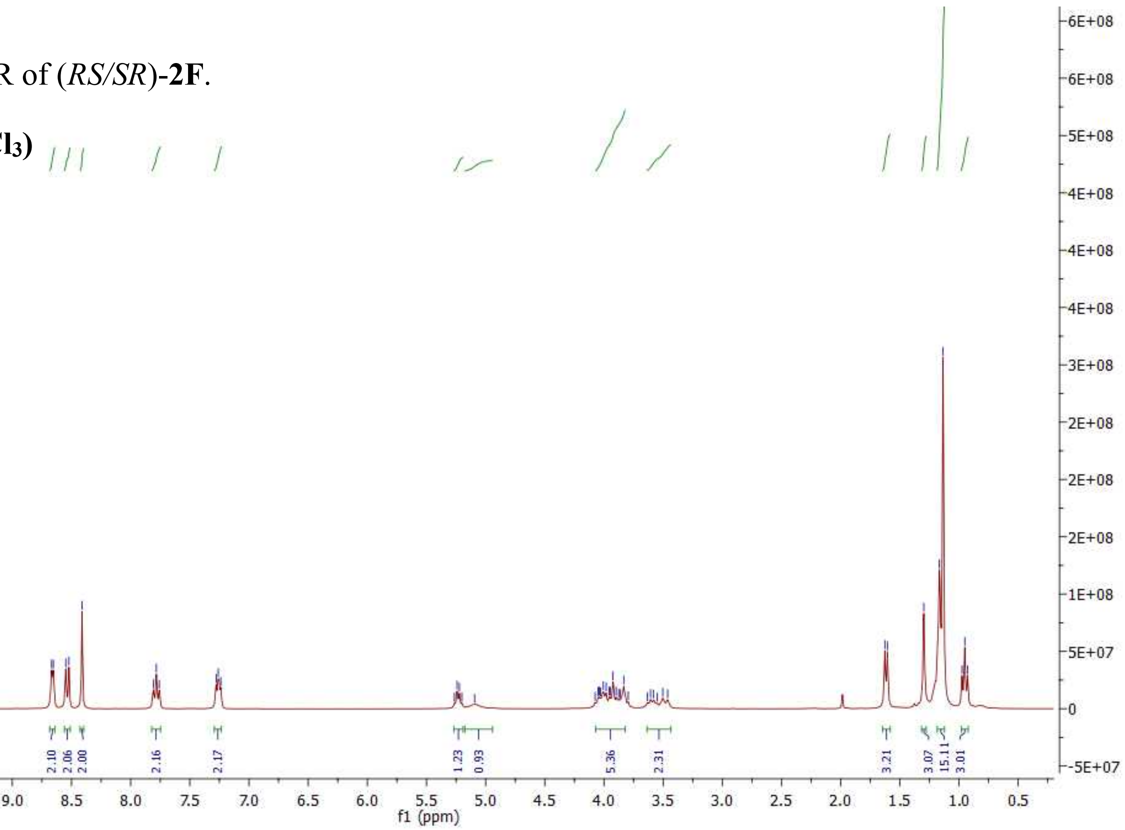

^{13}C-NMR ($CDCl_3$)

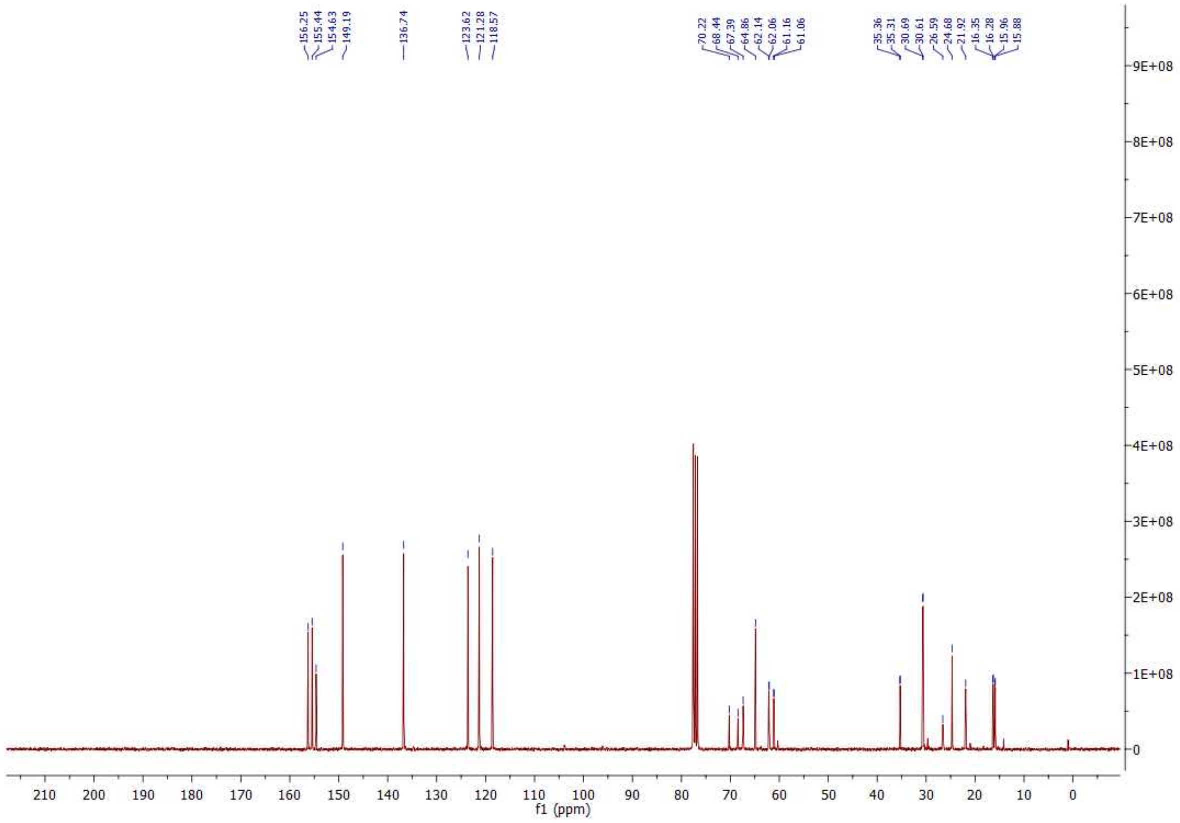

^{31}P-NMR ($CDCl_3$)

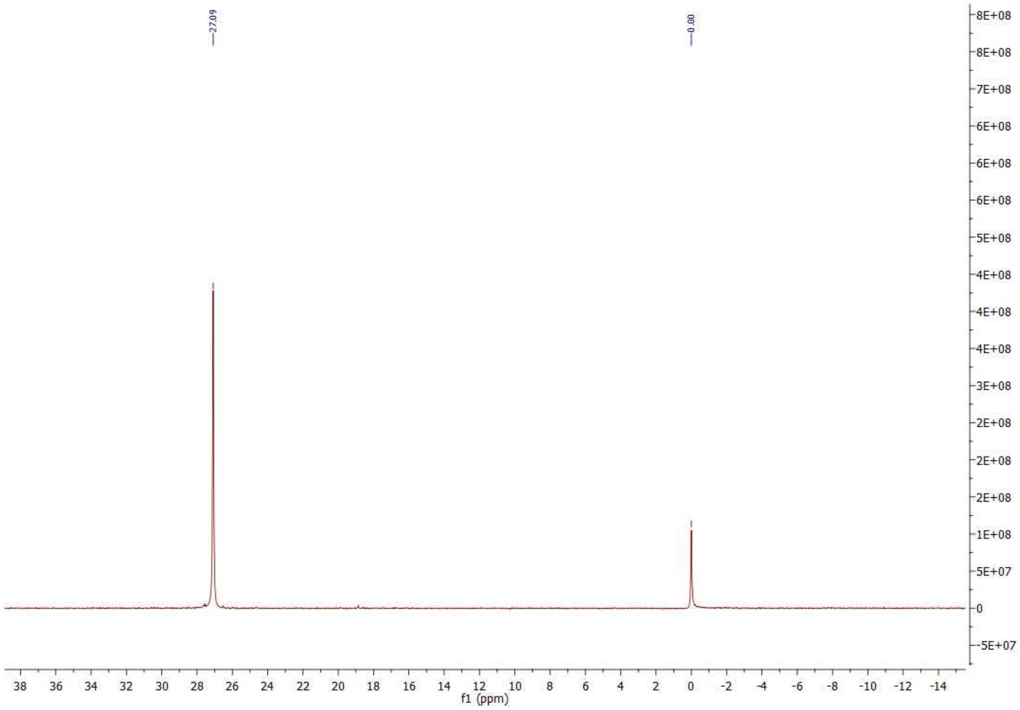

Figure S7. NMR of (*RR/SS*)-**2F**.

^{1}H-NMR ($CDCl_3$)

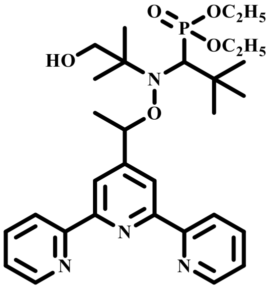

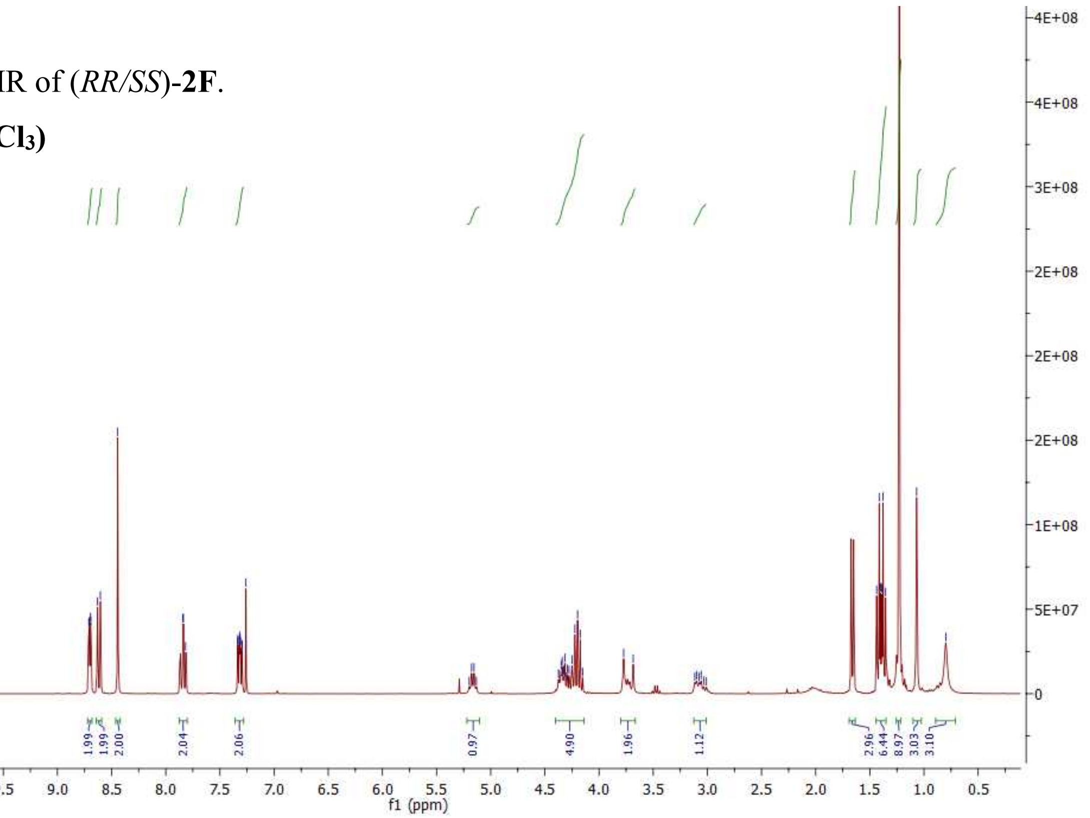

^{13}C-NMR ($CDCl_3$)

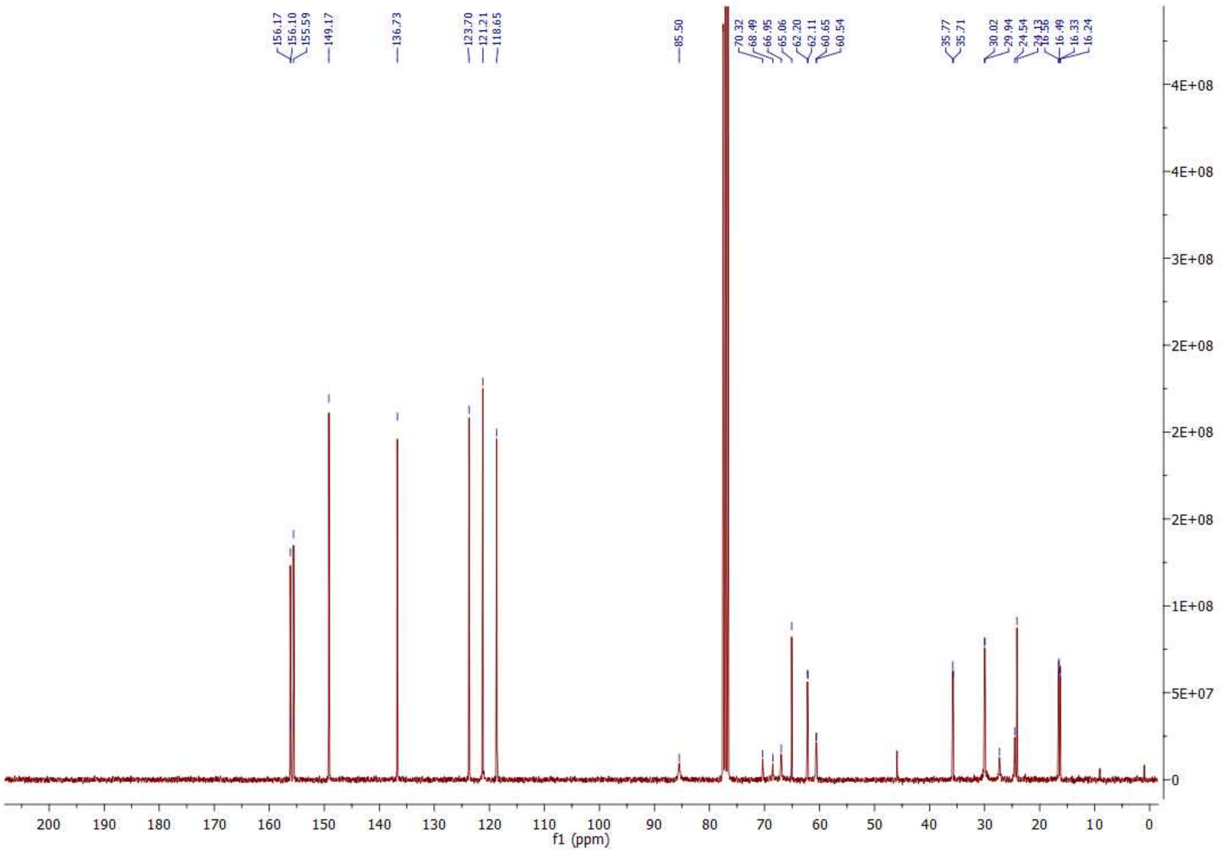

^{31}P-NMR ($CDCl_3$)

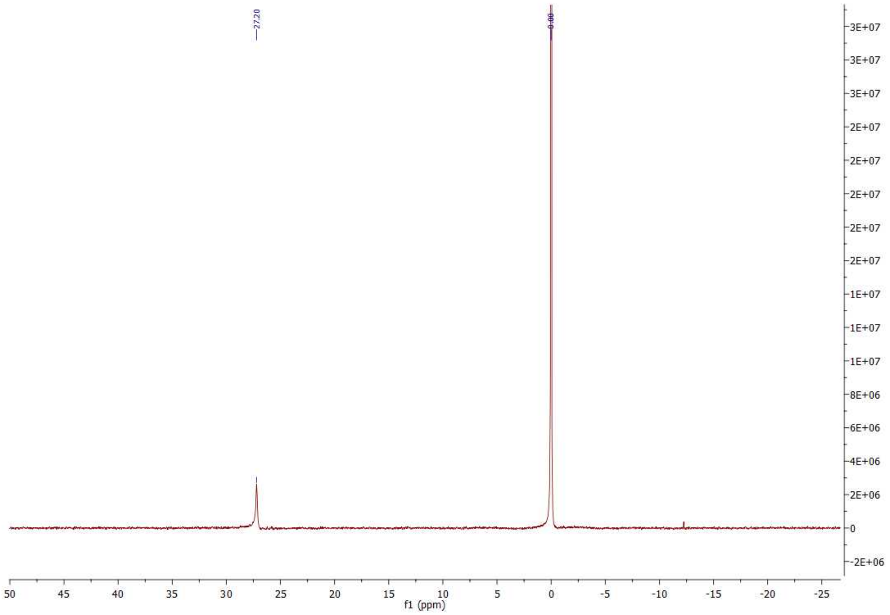

Figure S8. NMR of (*RS/SR*)-aldehyde derivative.

^{1}H-NMR ($CDCl_3$)

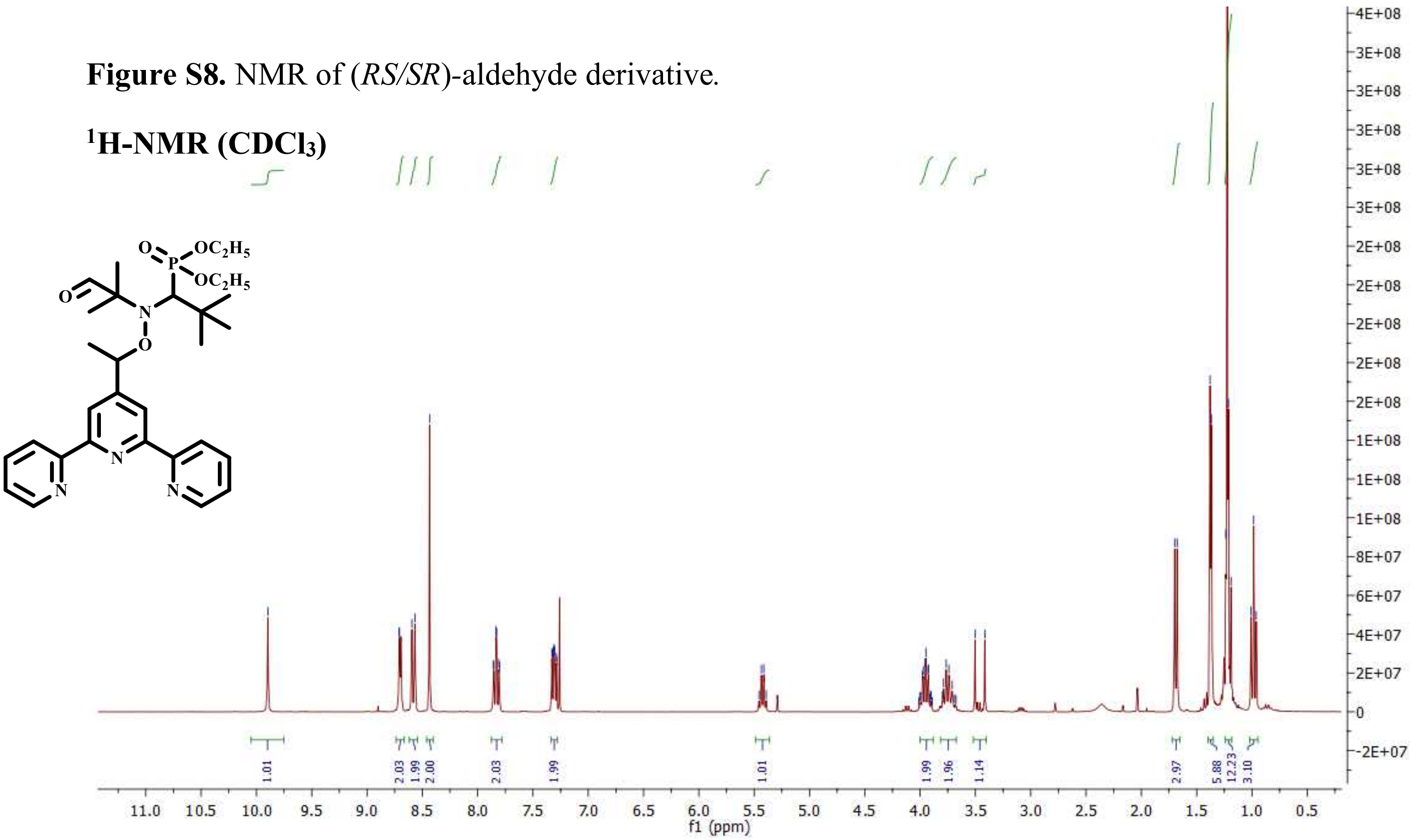

^{13}C-NMR ($CDCl_3$)

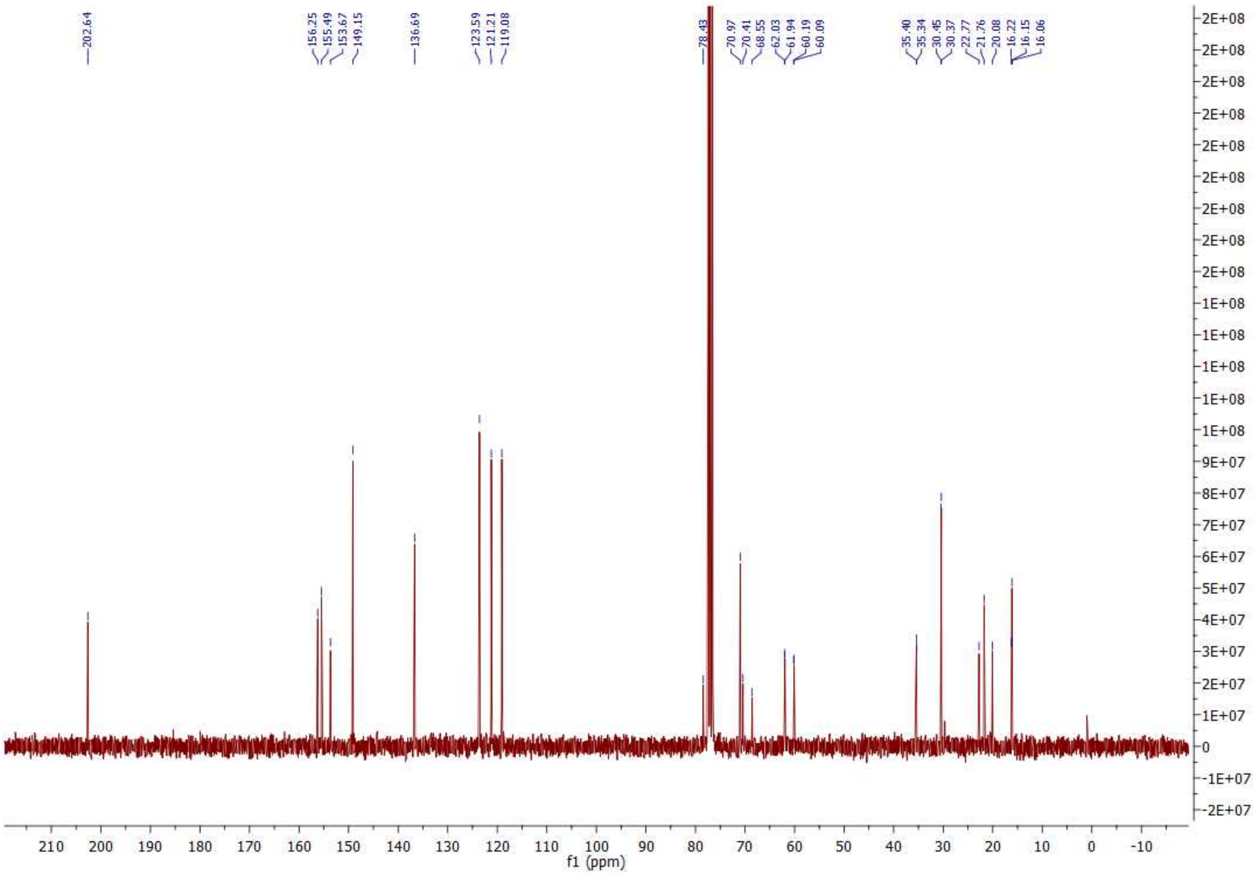

^{31}P-NMR ($CDCl_3$)

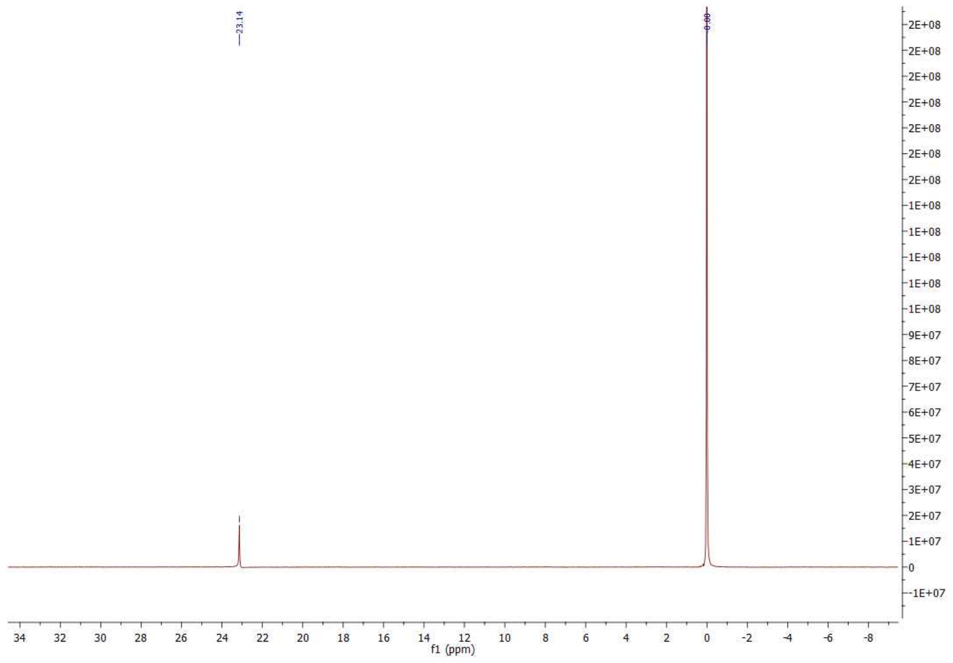

Figure S9. NMR of (*RR/SS*)-aldehyde derivative.

^{1}H-NMR ($CDCl_3$)

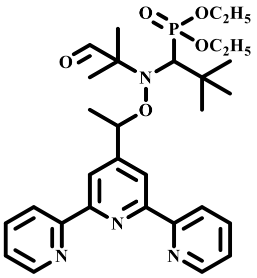

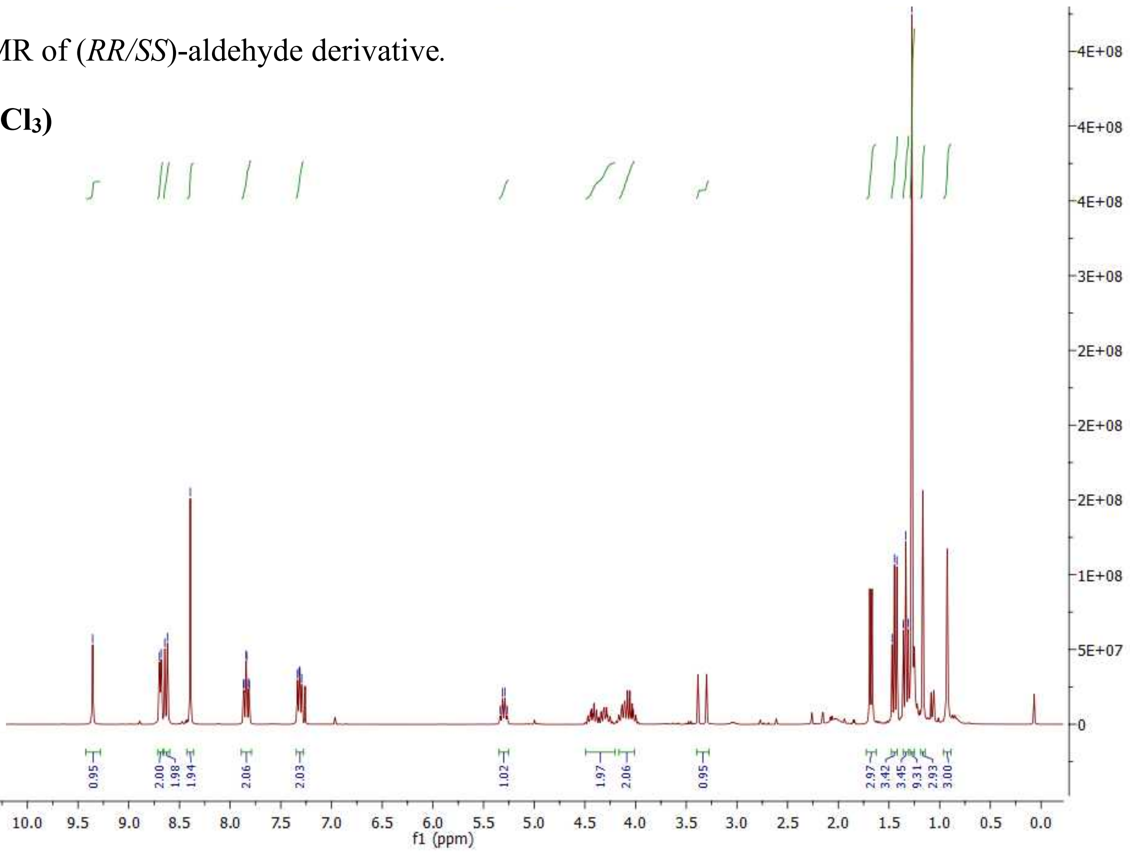

^{13}C-NMR ($CDCl_3$)

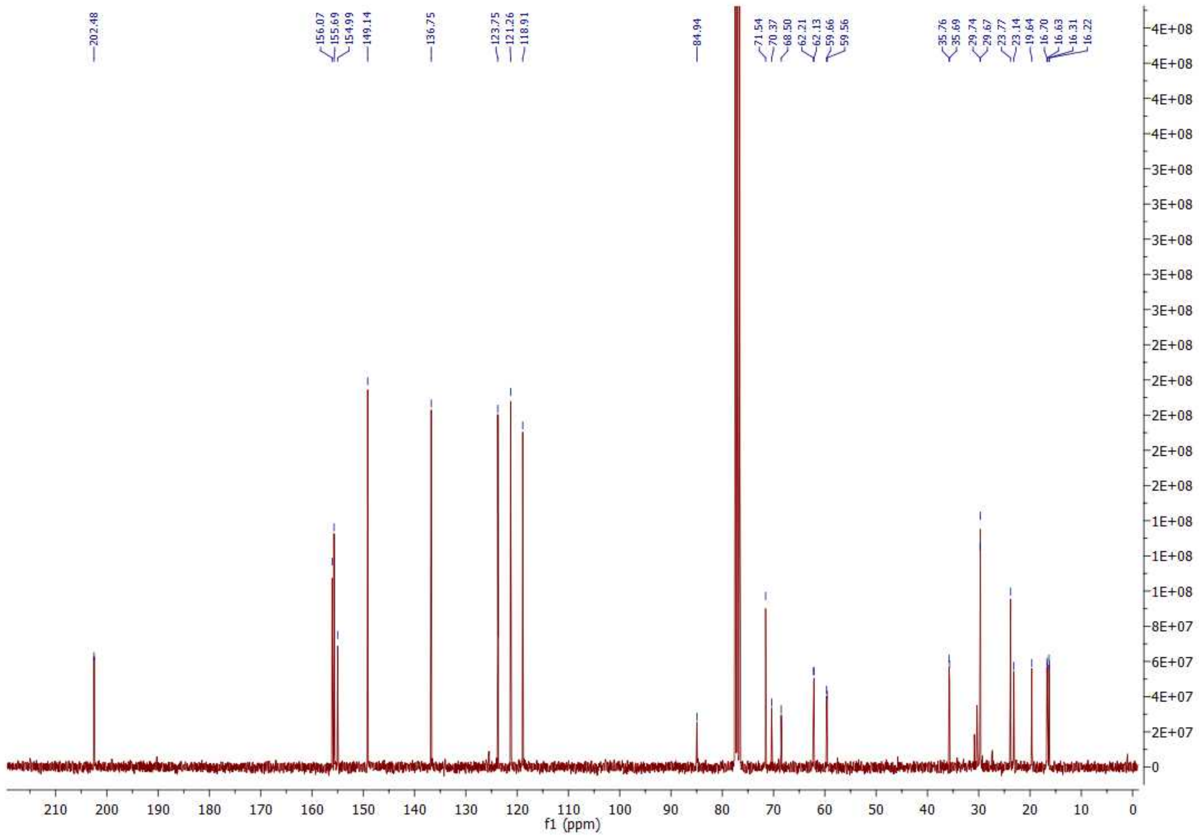

^{31}P-NMR ($CDCl_3$)

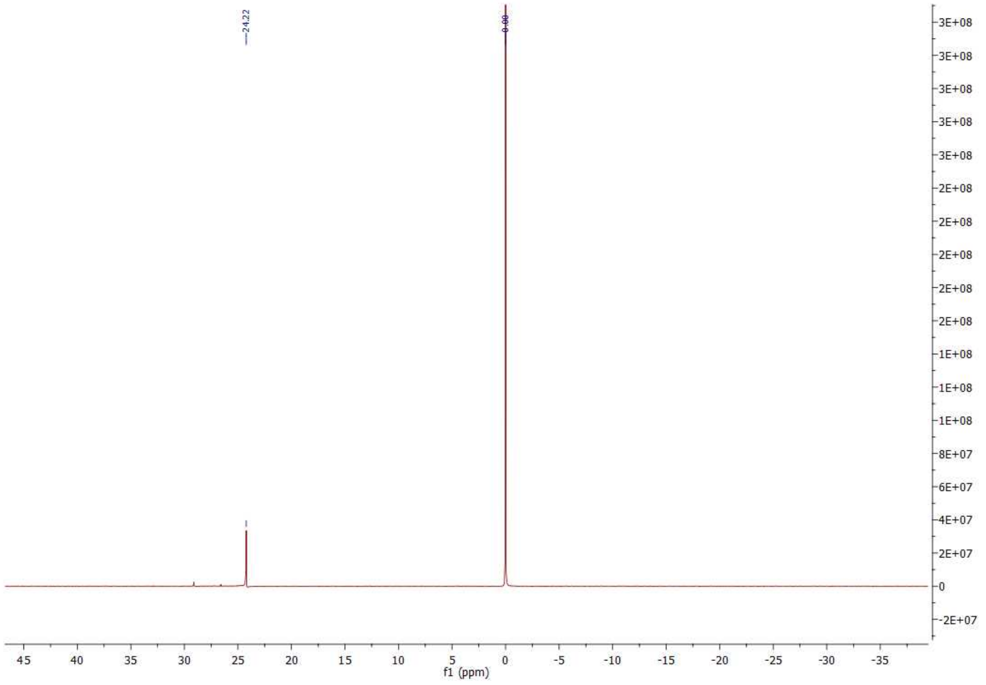

Figure S10. NMR of (*RS/SR*)-**4F**.

^{1}H-NMR ($CDCl_3$)

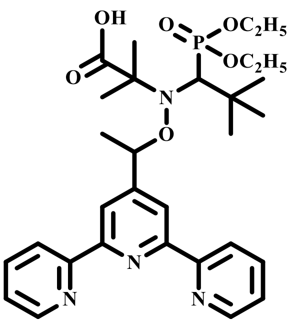

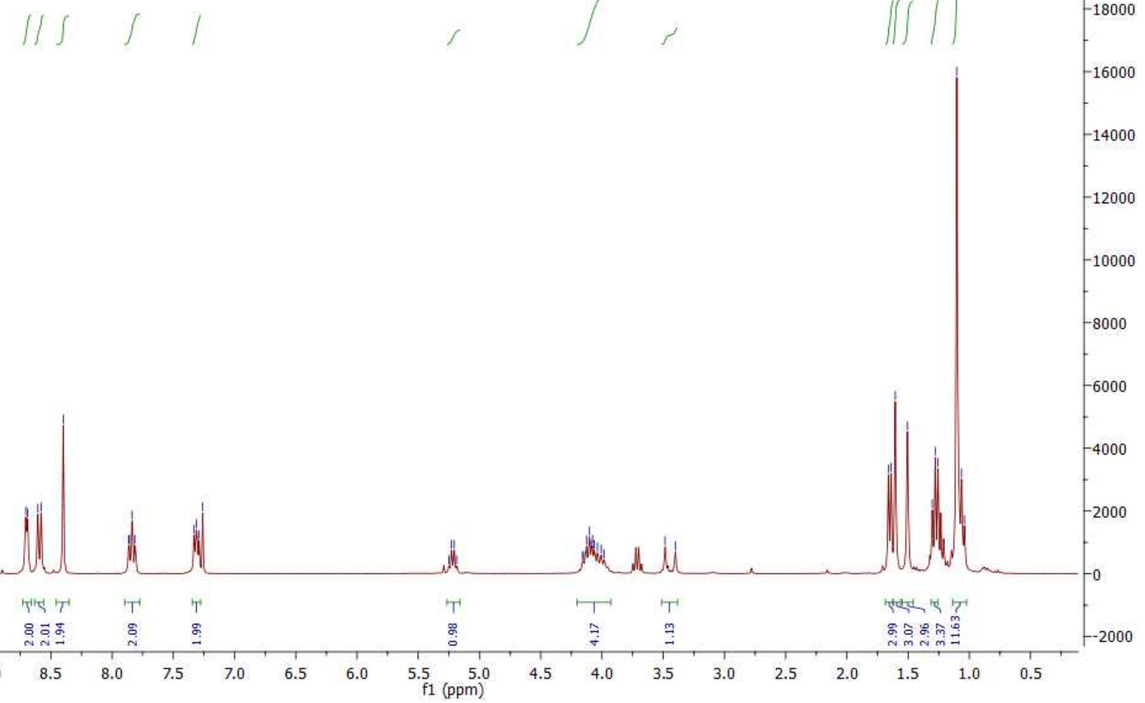

^{13}C-NMR ($CDCl_3$)

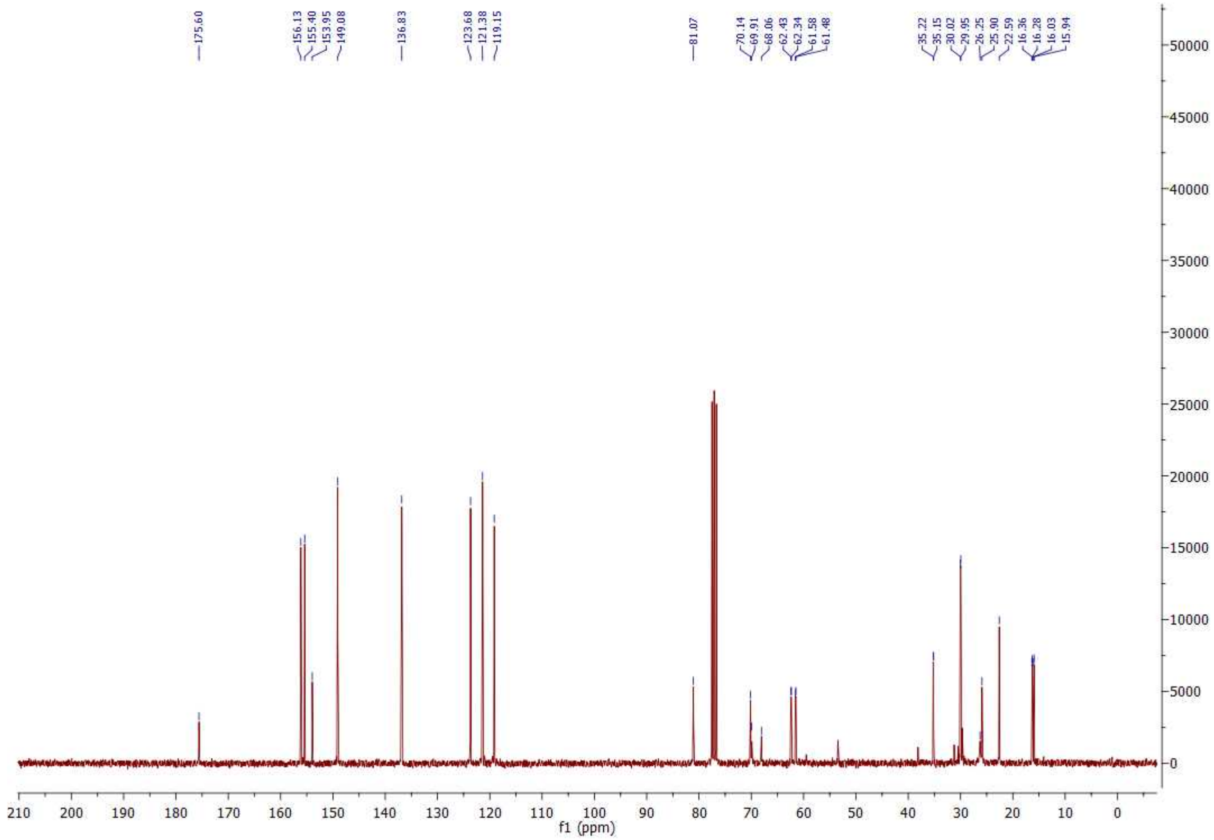

^{31}P-NMR ($CDCl_3$)

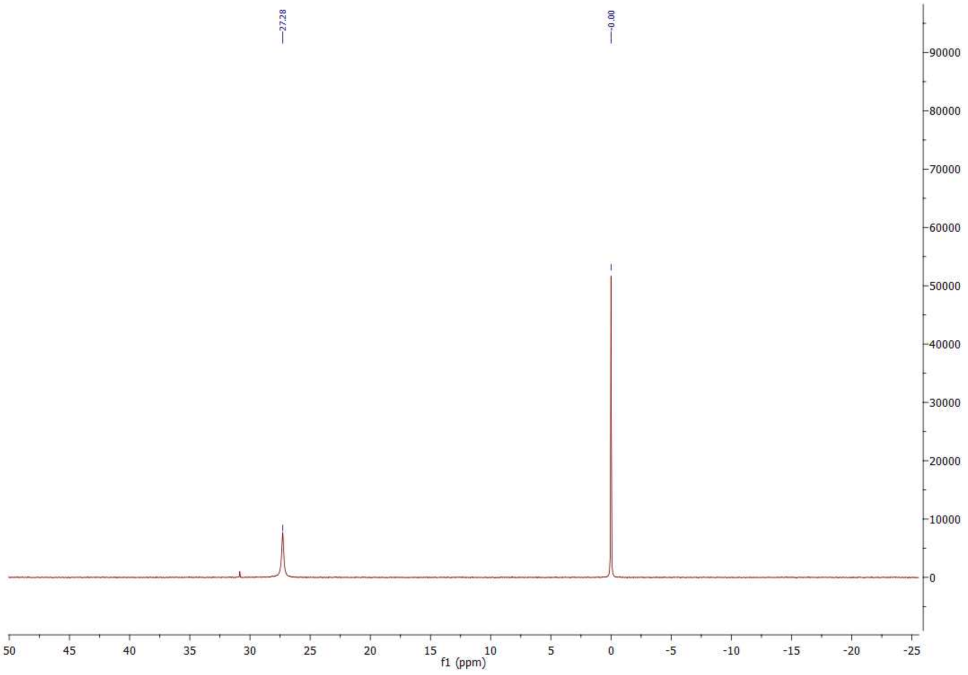

Figure S11. NMR of (*RR/SS*)-**4F**.

^{1}H-NMR (CDCl$_3$)

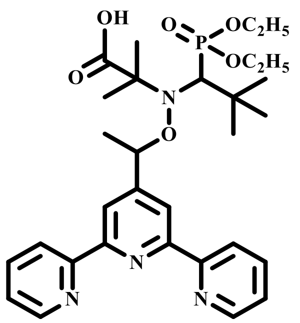

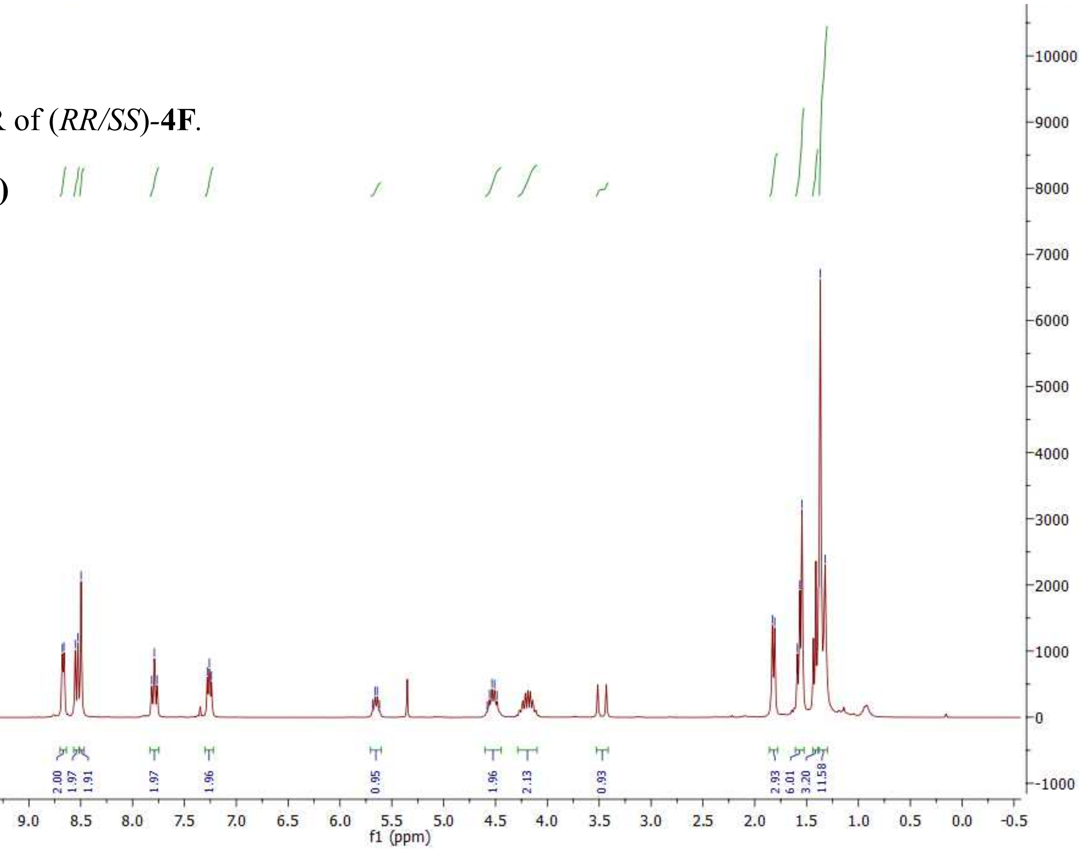

^{13}C-NMR (CDCl$_3$)

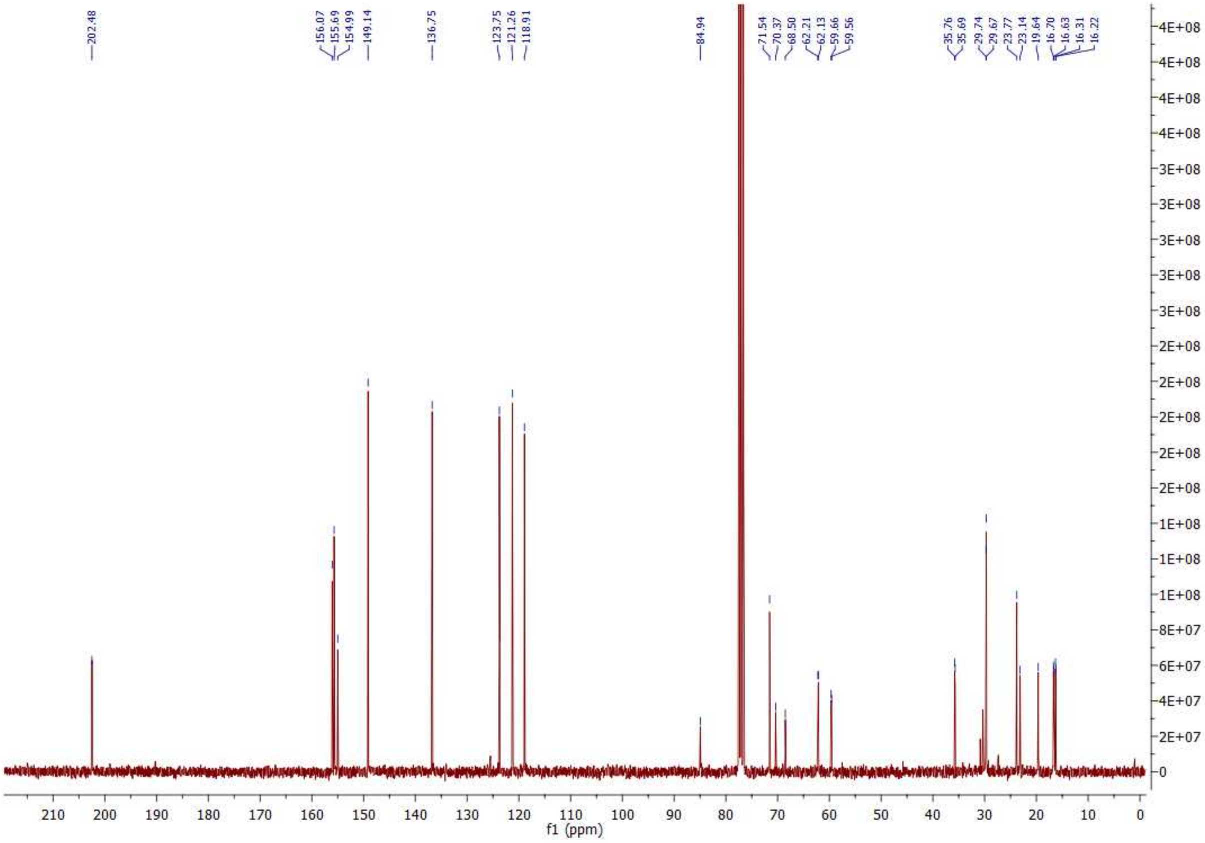

^{31}P-NMR ($CDCl_3$)

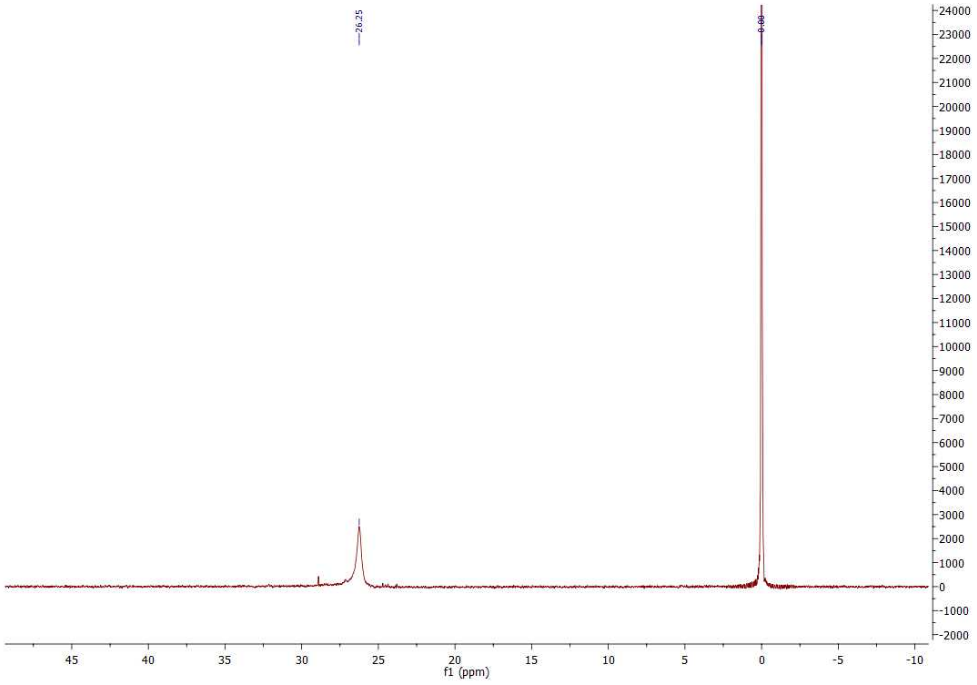

Figure S12. NMR of (*R/S*)-**8F**

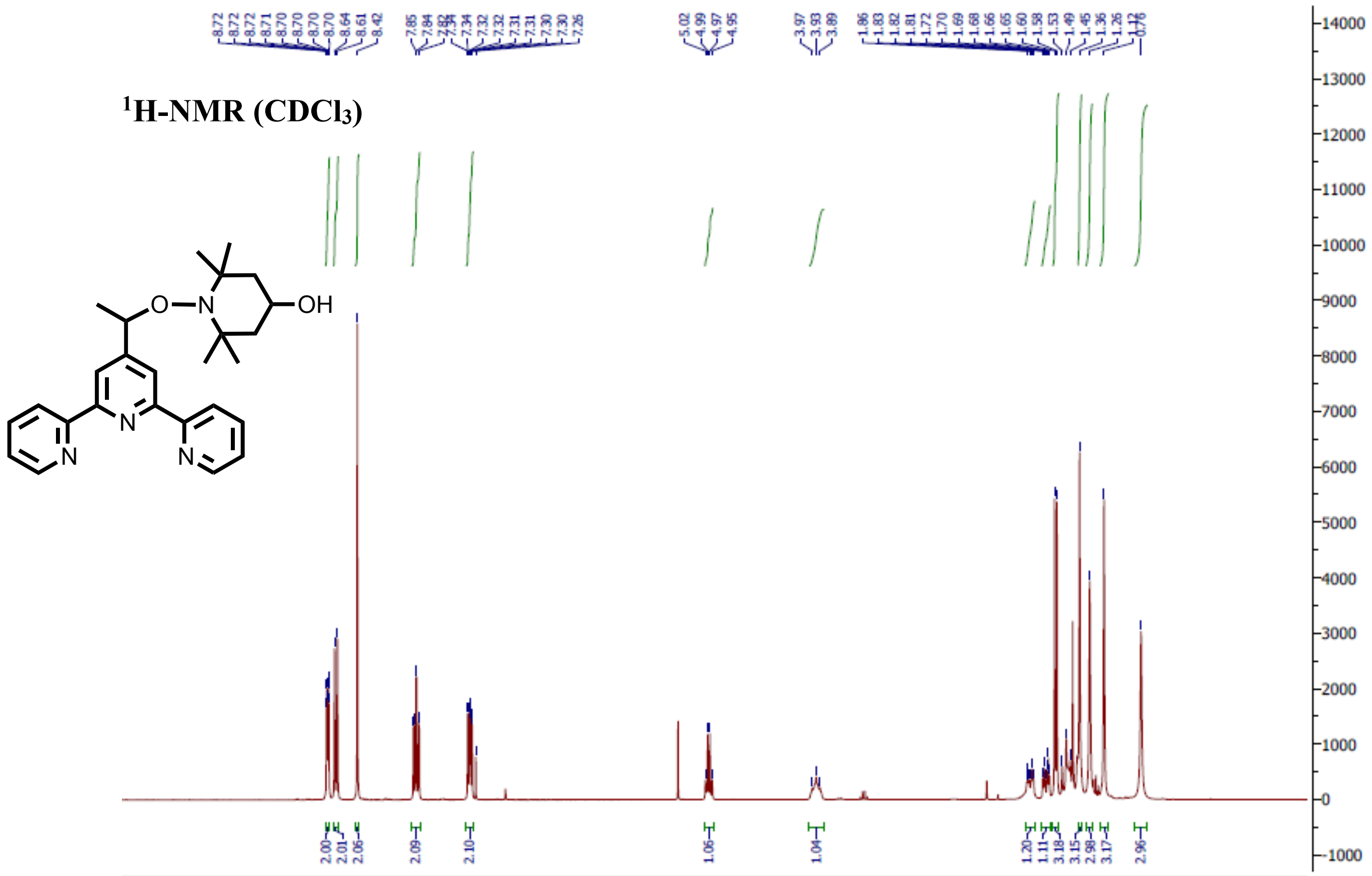

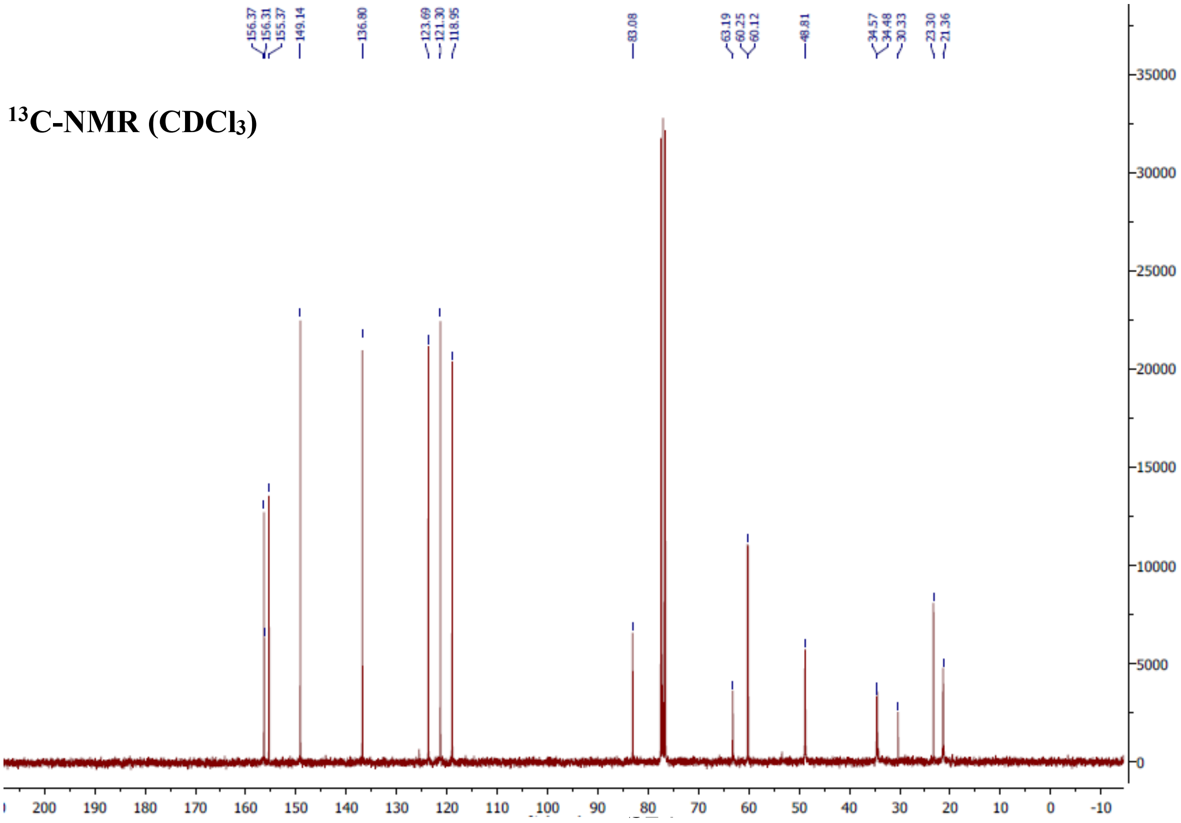

Figure S13. NMR of (*RS*/*SR*)-**6G**.

^{1}H-NMR (CDCl$_3$)

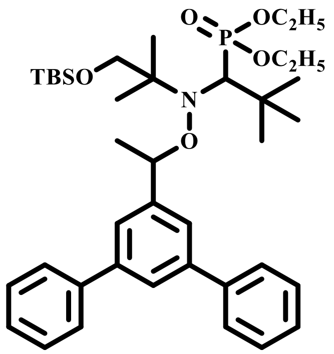

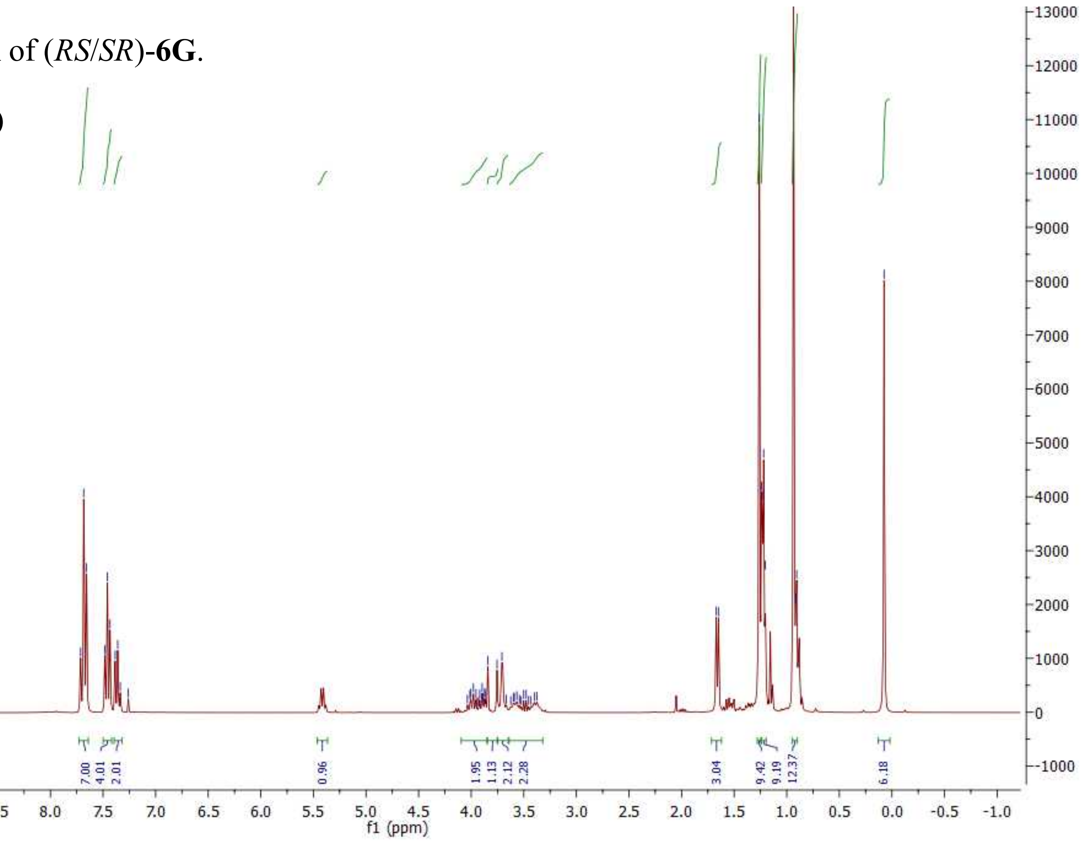

^{13}C-NMR (CDCl$_3$)

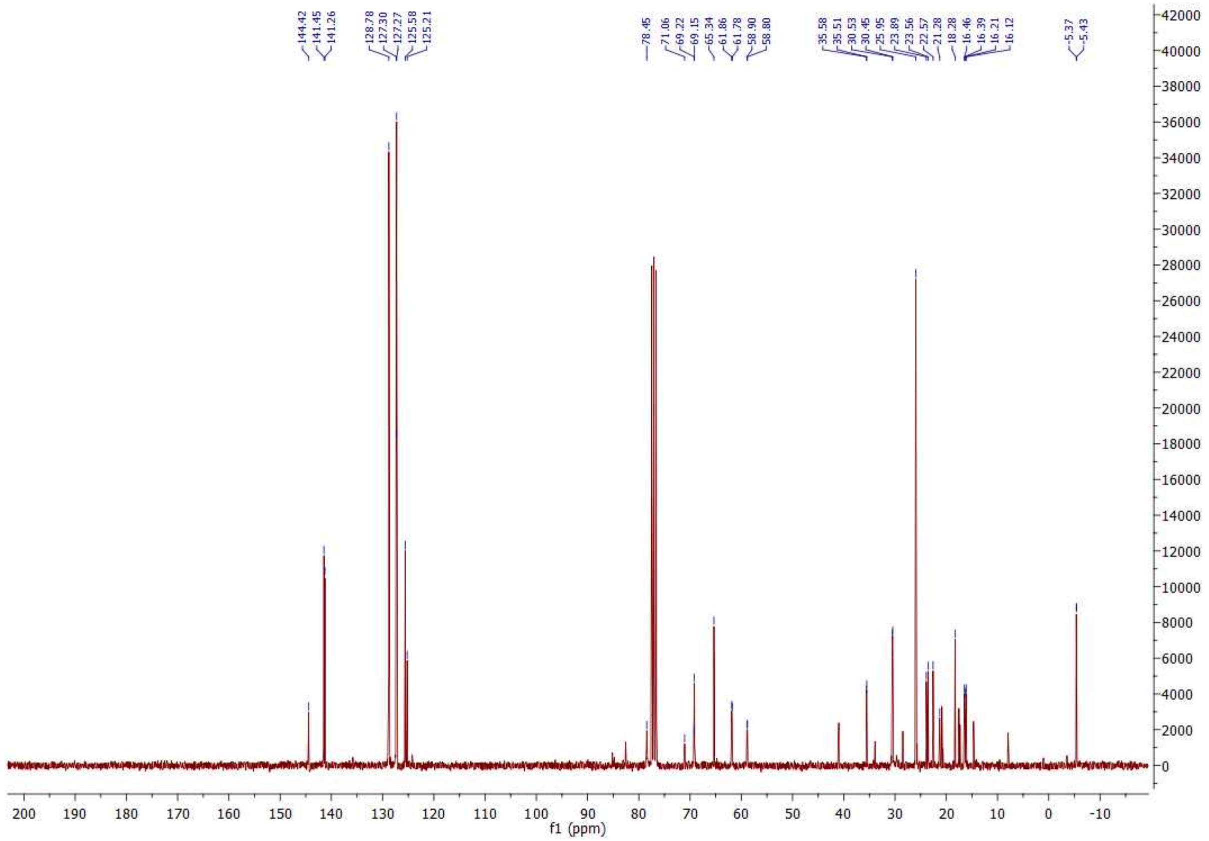

^{31}P-NMR ($CDCl_3$)

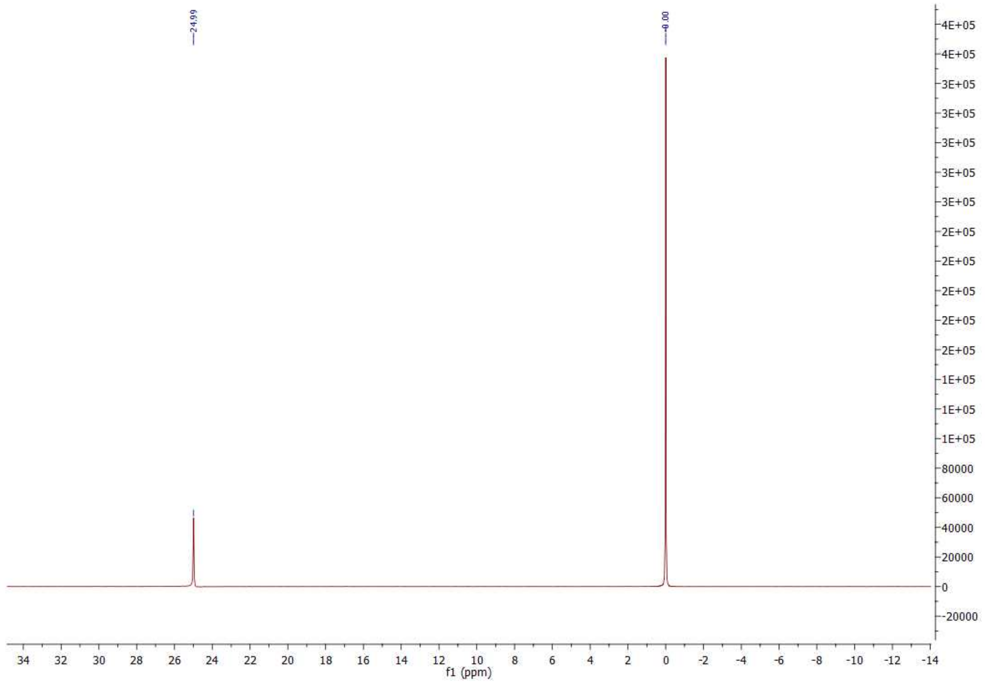

Figure S14. NMR of (*RR*/*SS*)-**6G**.

^{1}H-NMR (CDCl$_3$)

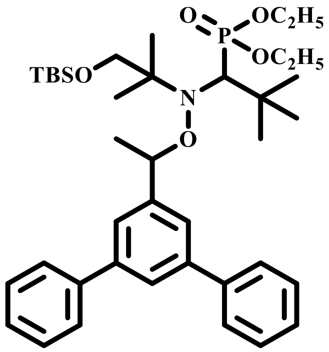

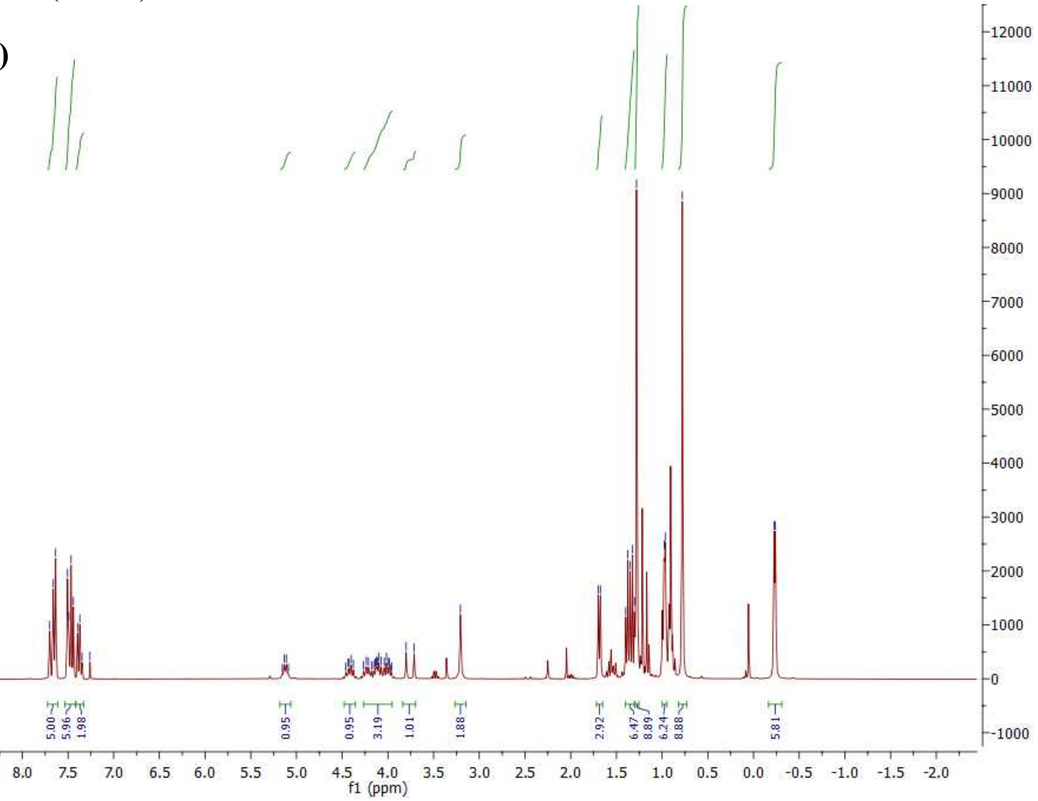

^{13}C-NMR (CDCl$_3$)

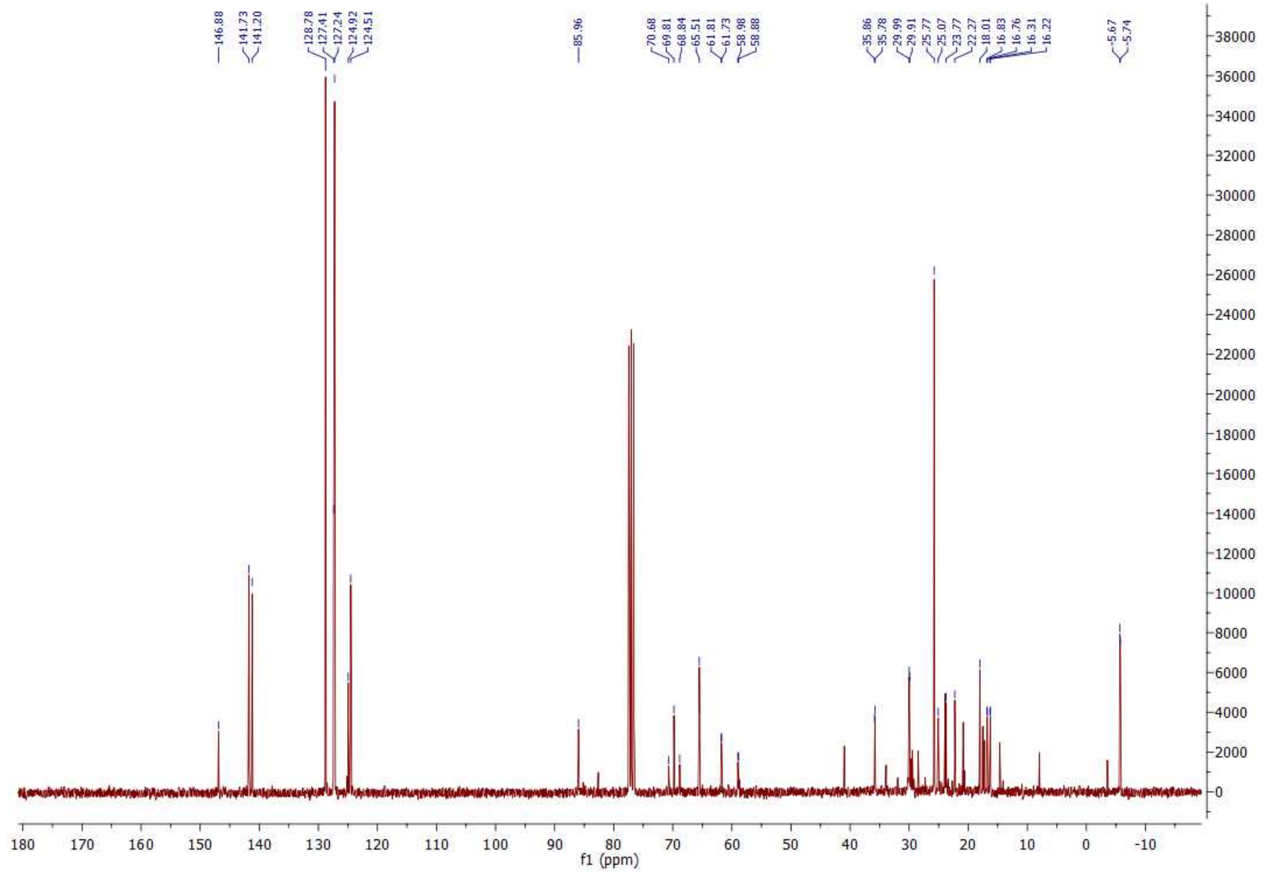

^{31}P-NMR ($CDCl_3$)

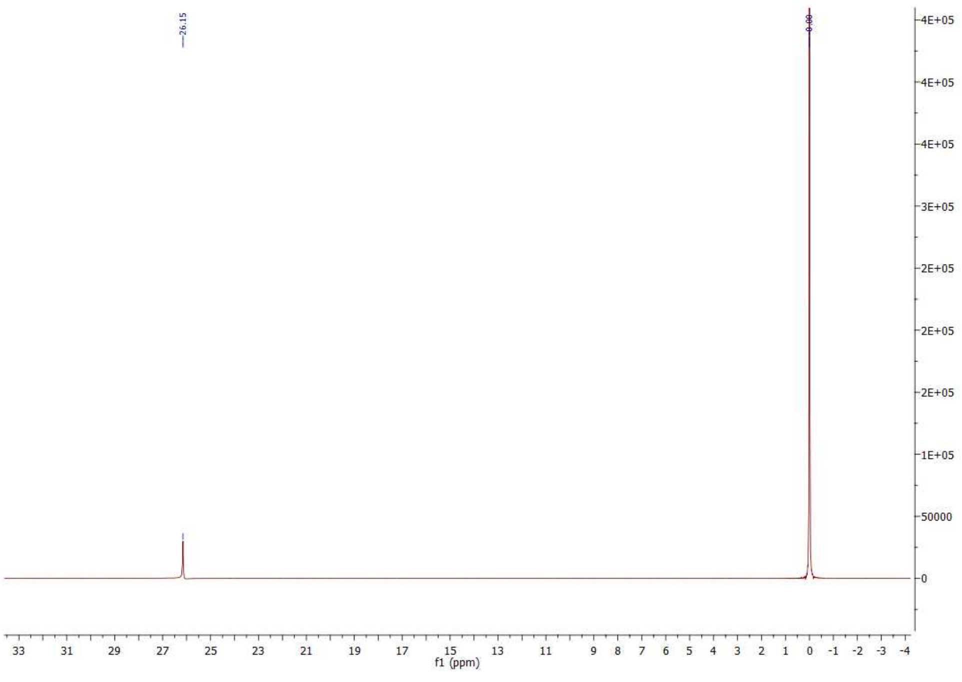

Figure S15. NMR of (*RS*/*SR*)-**2G**.

^{1}H-NMR ($CDCl_3$)

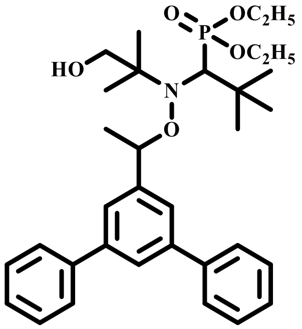

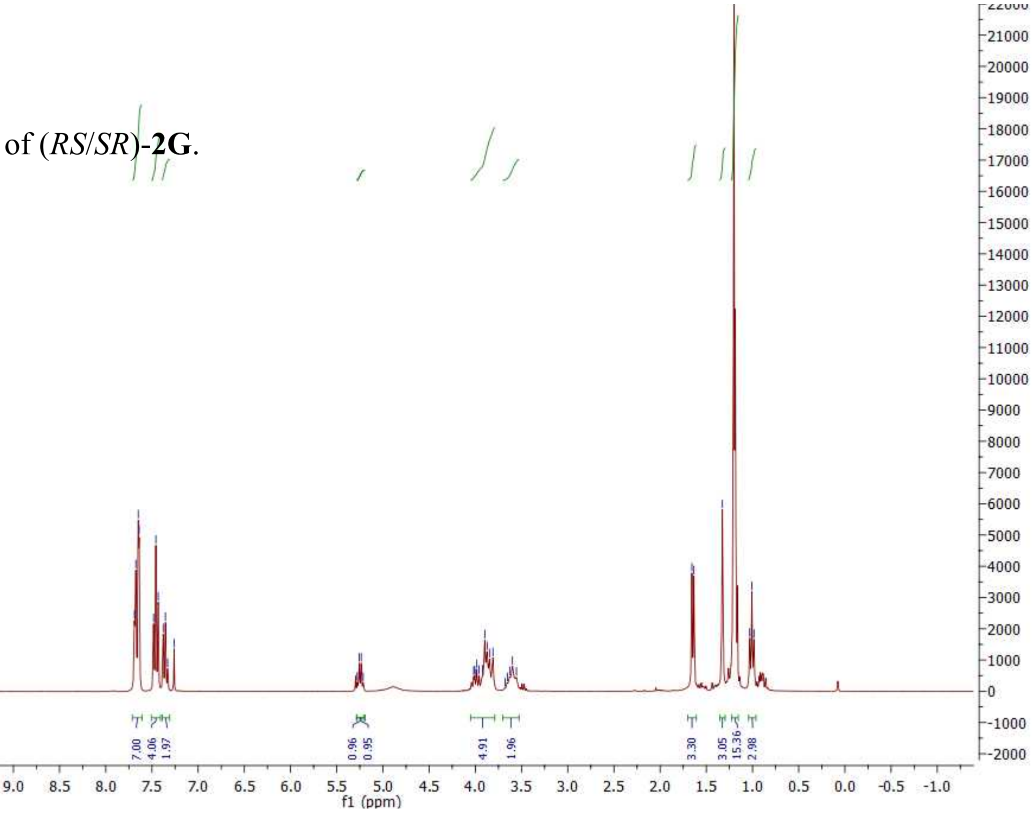

^{13}C-NMR ($CDCl_3$)

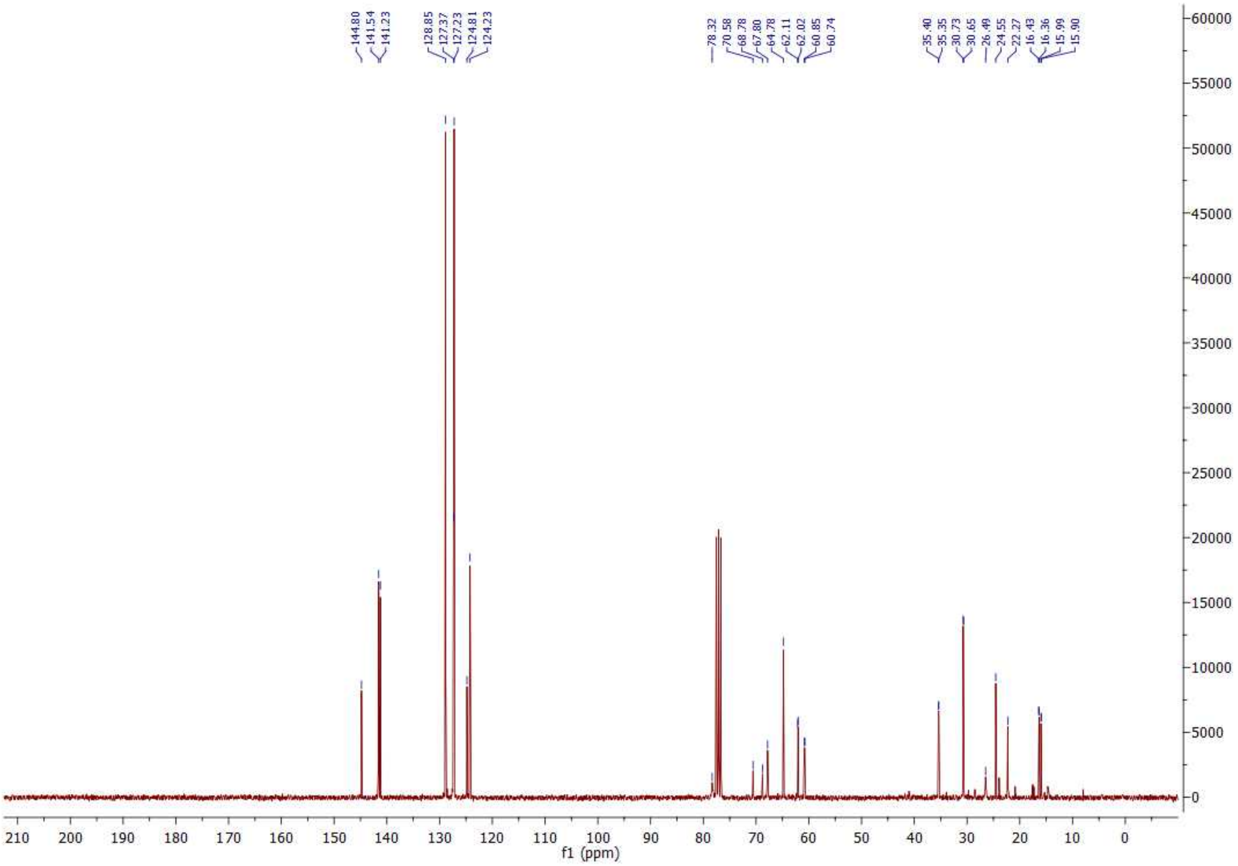

^{31}P-NMR ($CDCl_3$)

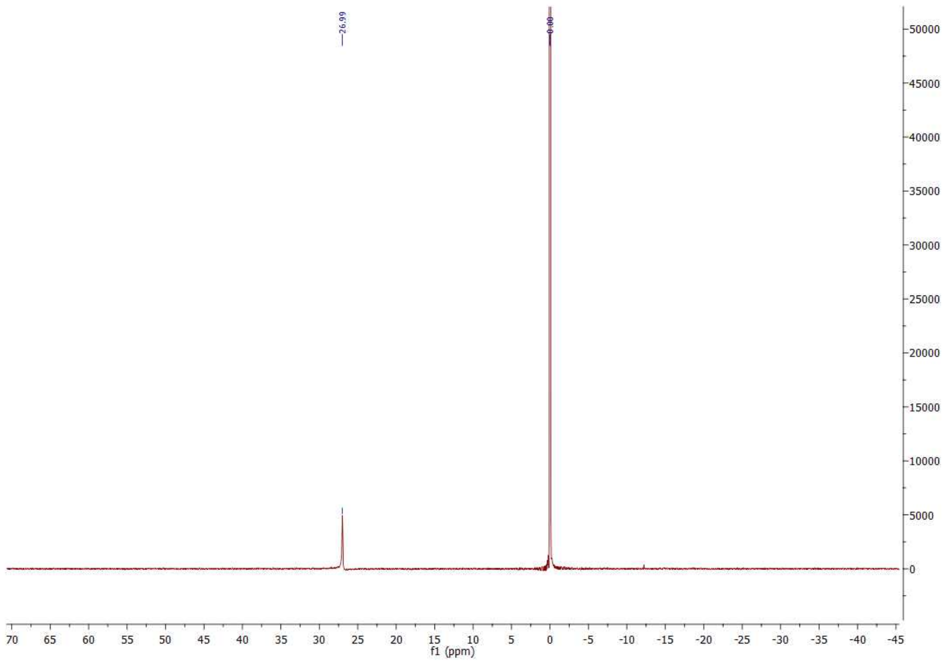

Figure S16. NMR of (*RR*/*SS*)-**2G**.

^{1}H-NMR (CDCl$_3$)

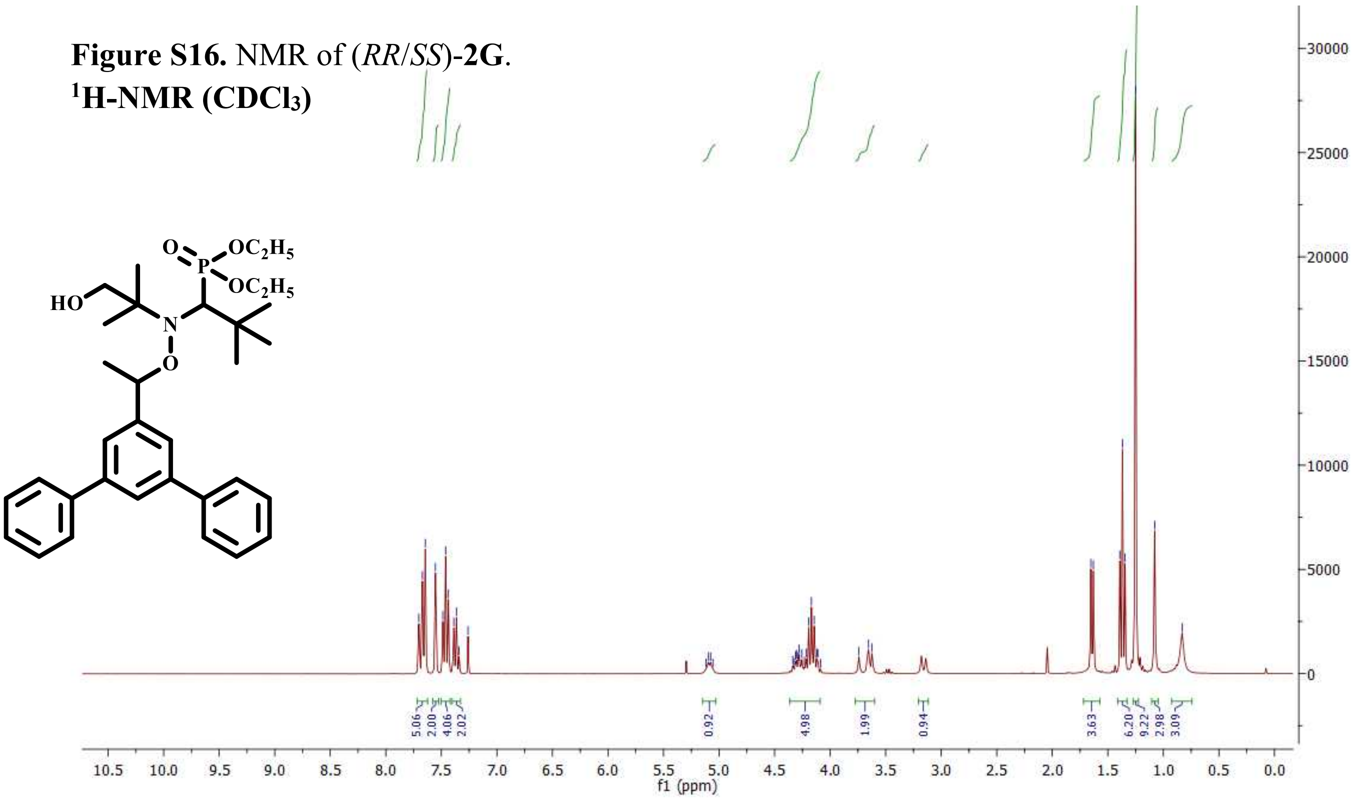

^{13}C-NMR (CDCl$_3$)

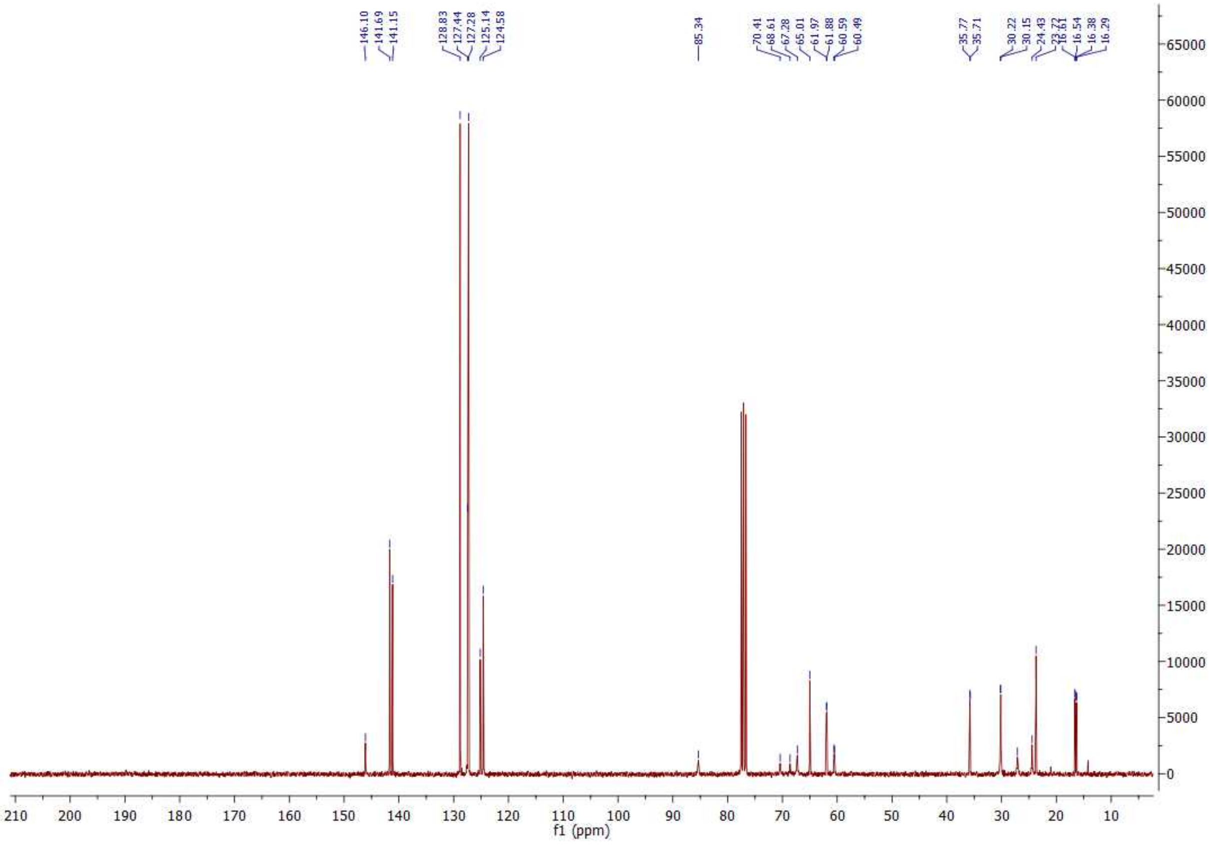

^{31}P-NMR ($CDCl_3$)

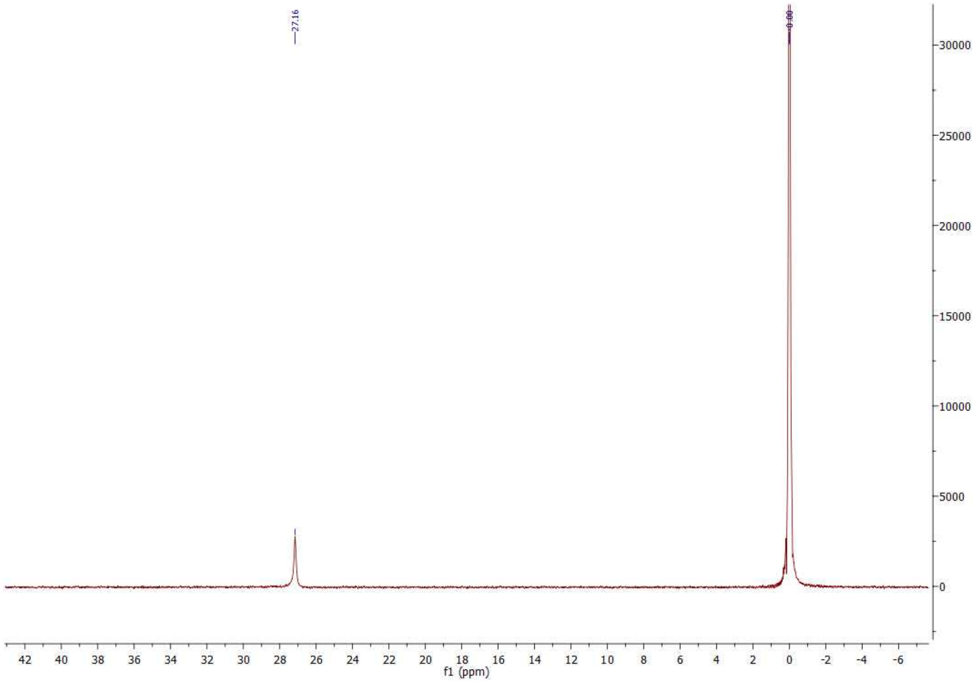

Table S2. XRD data for (*RR*/*SS)*-**6F**

Compound	**(*RR*/*SS*)-6F**
Empirical formula	$C_{36}H_{57}N_4O_5PSi$
Formula weight	684.91
Temperature/K	293
Crystal system	monoclinic
Space group	$P2_1/c$
a/Å	20.9011(7)
b/Å	14.8947(5)
c/Å	13.4391(4)
α/°	90
β/°	107.315(4)
γ/°	90
Volume/Å^3	3994.2(2)
Z	4
ρ_{calc}g/cm^3	1.139
μ/mm^{-1}	0.141
F(000)	1480.0
Crystal size/mm^3	0.5 × 0.28 × 0.28
Radiation	MoKα (λ = 0.71073)
2Θ range for data collection/°	5.838 to 52.744
Index ranges	$-26 \le h \le 25$, $-18 \le k \le 18$, $-16 \le l \le 16$
Reflections collected	52925
Independent reflections	8087 [R_{int} = 0.1388, R_{sigma} = 0.0555]
Data/restraints/parameters	8087/15/432
Goodness-of-fit on F^2	1.084
Final R indexes [I>=2σ (I)]	R_1 = 0.0910, wR_2 = 0.2628
Final R indexes [all data]	R_1 = 0.1166, wR_2 = 0.3005
Largest diff. peak/hole / e Å^{-3}	0.81/-0.77

Table S3. XRD data for (*RR*/*SS)*-**6G**.

Compound	**(*RR*/*SS*)-6G**
Empirical formula	$C_{33}H_{46}NO_5P$
Formula weight	567.68
Temperature/K	295
Crystal system	monoclinic
Space group	Pc
a/Å	11.7459(5)
b/Å	18.2946(7)
c/Å	16.2012(6)
α/°	90
β/°	110.150(4)
γ/°	90
Volume/Å^3	3268.3(2)
Z	4
ρ_{calc}g/cm^3	1.154
μ/mm^{-1}	1.049
F(000)	1224.0
Crystal size/mm^3	0.26 × 0.2 × 0.12
Radiation	CuKα (λ = 1.54184)
2Θ range for data collection/°	7.558 to 142.748
Index ranges	-14 ≤ h ≤ 14, -22 ≤ k ≤ 22, -19 ≤ l ≤ 19
Reflections collected	27391
Independent reflections	9765 [R_{int} = 0.0417, R_{sigma} = 0.0321]
Data/restraints/parameters	9765/45/884
Goodness-of-fit on F^2	1.023
Final R indexes [I>=2σ (I)]	R_1 = 0.0773, wR_2 = 0.2160
Final R indexes [all data]	R_1 = 0.0829, wR_2 = 0.2293
Largest diff. peak/hole / e Å^{-3}	0.91/-0.32
Flack parameter	0.01(3)

Figure S17. Proposed mechanism for the formation of compounds **24** and **25** (see Oxygen Radicals, J. K. Kochi, in *Free Radicals*, J. K. Kochi, ed., John Wiley & Sons (1973), Vol. II, p. 686ss.

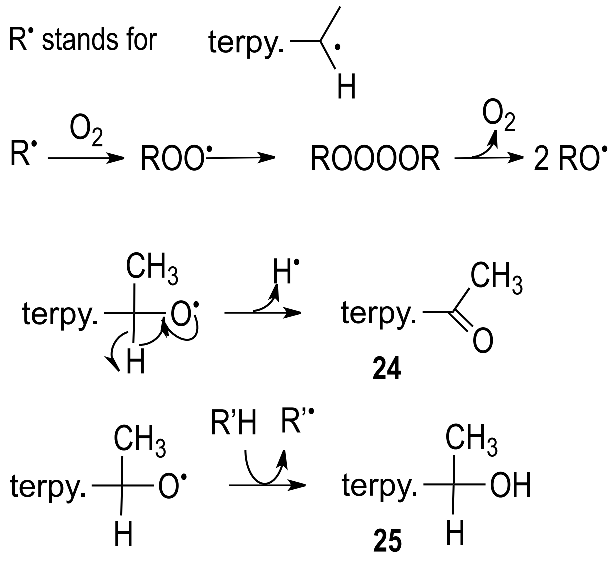

Figure S18. ^{1}H NMR spectrum of (*RS/SR*)-**2F** and (*RS/SR*)-**2FH**$^+$ in *t*-BuPh.

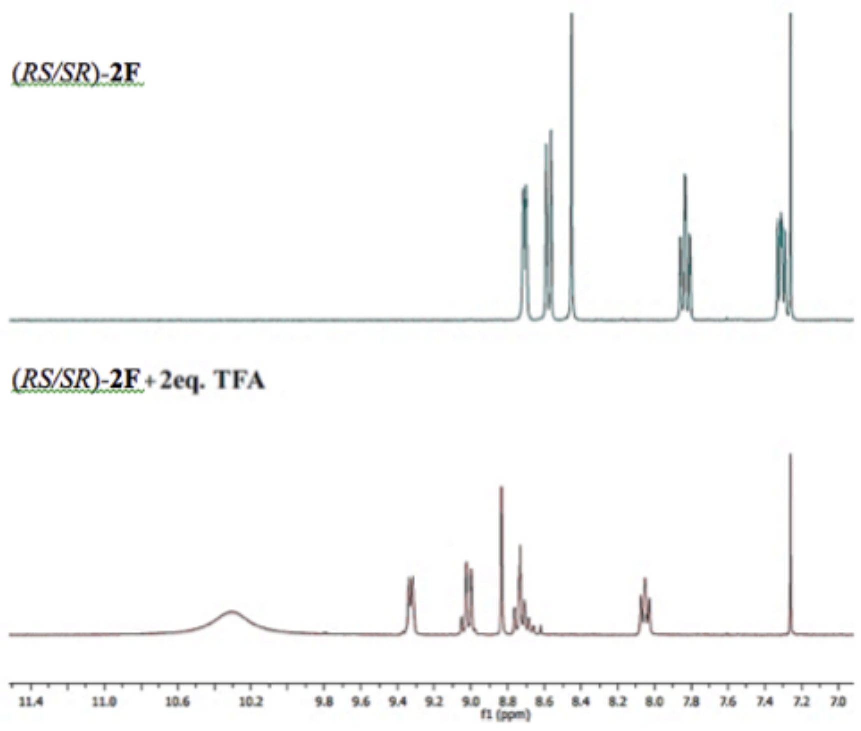

Figure S19. ^{1}H NMR spectrum of (*RR/SS*)-**2F** and (*SS/RR*)-**2FH**$^{+}$ in *t*-BuPh.

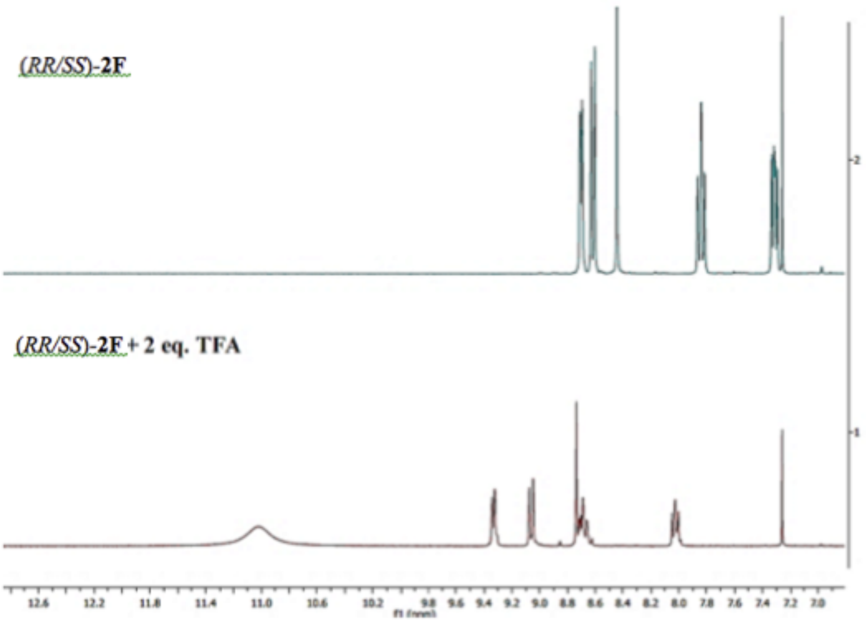

Supplement: Supplementary file 1 [file molecules-25-03838-s001.pdf]
